# Allenylphosphine oxides as simple scaffolds for phosphinoylindoles and phosphinoylisocoumarins

**DOI:** 10.3762/bjoc.10.99

**Published:** 2014-05-02

**Authors:** G Gangadhararao, Ramesh Kotikalapudi, M Nagarjuna Reddy, K C Kumara Swamy

**Affiliations:** 1 School of Chemistry, University of Hyderabad, Hyderabad 500 046, A. P., India. Fax: (+91)-40-23012460

**Keywords:** allenes, indoles, isocoumarins, organophosphorus, phosphinoyl-heterocycles, propargyl alcohols

## Abstract

A range of phosphinoylindoles was prepared in one-pot from functionalized propargyl alcohols and a suitable P(III) precursor via a base-mediated reaction. The reaction proceeds via the intermediacy of allenylphosphine oxides. Similarly, phosphinoylisocoumarins were prepared from allenylphosphine oxides in a trifluoroacetic acid-mediated reaction; the latter also acts as a solvent. Interestingly, in the presence of wet trifluoroacetic acid, in addition to phosphinoylisocoumarins, phosphorus-free isocoumarins were also obtained. Key products were characterized by single crystal X-ray crystallography.

## Introduction

**Scheme 1:**
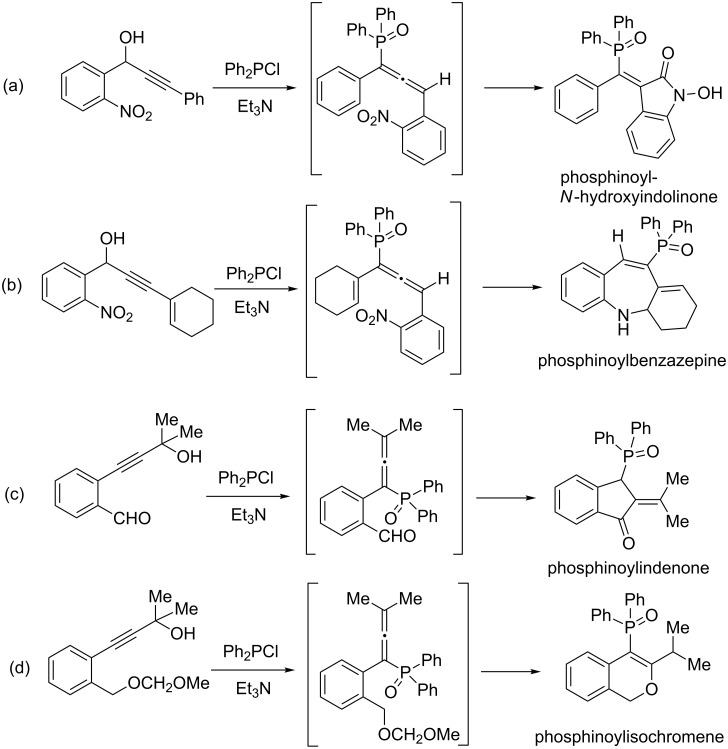
Reaction of P(III)-Cl precursors with propargyl alcohols leading to phosphorus based (a) *N*-hydroxyindolinone, (b) benzazepine, (c) indenone and (d) isochromene via allenic intermediates.

Allenes, by virtue of cumulative double bonds that facilitate reactions with diverse classes of substrates, are versatile building blocks from a synthetic perspective [1–2]. They are also found in many natural products, pharmaceuticals [3] and molecular materials [4]. Thus, over the last decade, allenes have attained a prominent position in organic transformations like cycloaddition, cycloisomerization, base or metal-catalyzed reactions [5–7]. In particular, cyclization reaction of allenes has emerged as a valuable tool in developing different methods leading to various carbo-/heterocycles [8–14]. Allenylphosphonates and allenylphosphine oxides, as a subclass of allenes, have also been utlized in several novel transformations [15–17]. It may also be noted that organophosphonates in addition have wide applications in medicinal chemistry [3,18–19] and as reagents in organic synthesis [20]. In our previous reports, we described the utility of phosphorus-based allenes in various cyclization reactions involving heteroatoms that could lead to phosphono/phosphinoyl hetero-/carbocycles [21–30]. The reported series include phosphonobenzofurans/indenones [21–22], -pyrazoles [23], -chromenes/thiochromenes [24–25], -pyrroles [26], multiply substituted furans [27], indolopyran-1-ones [28], *N*-hydroxyindolinones [29], and oxindoles [30]. In the reaction shown in Scheme 1a, for the formation of the phosphinoylindolinone, one of the oxygen atoms of the nitro group has been moved to a carbon [29]. The reaction shown in Scheme 1b led to rather previously unsuspected and unexpected benzazepines as products. After the elimination of a CO_2_ molecule, this reaction also features an unprecedented rearrangement involving the interemdiate allene [29]. Many other unusual transformations have also been reported recently [31]. In another reaction leading to phosphinoylindenone depicted in Scheme 1c, an intramolecular *ene*-reaction is possibly involved and in Scheme 1d the reaction led to phosphinoylisochromenes via deprotection of an allene intermediate under Lewis acid mediation [22]. In this context it was of interest to see, in a reaction like that shown in Scheme 1c, whether the introduction of an amide or a carboxylate ester in place of the –CHO group could lead to phosphinoyl-subtstituted indoles/isocoumarins via allenic intermediates or not. It is pertinent to note that indoles and isocoumarins are core structures found in many natural and pharmacological products [32–34]. Thus in this paper, we wish to report simple synthetic routes to phosphinoylindoles, and -isocoumarins utilizing functionalized allenylphosphine oxides/allenylphosphonates.

## Results and Discussion

**Figure 1:**
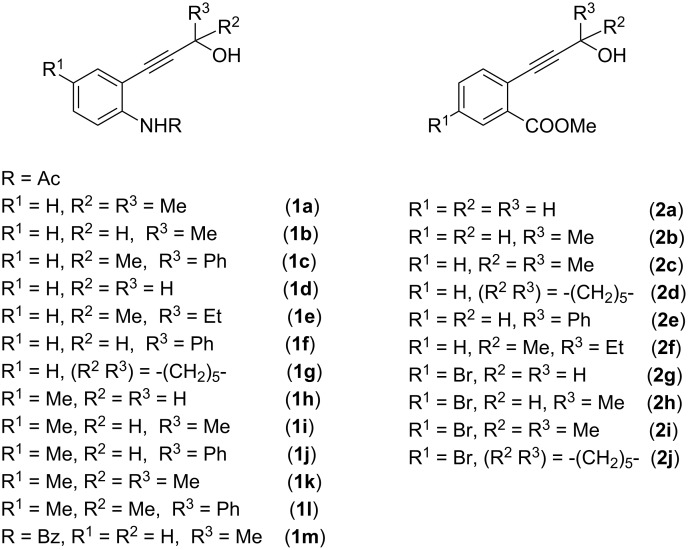
Functionalized propargyl alcohols **1a–m** and **2a–j** used in the present study.

**Scheme 2:**
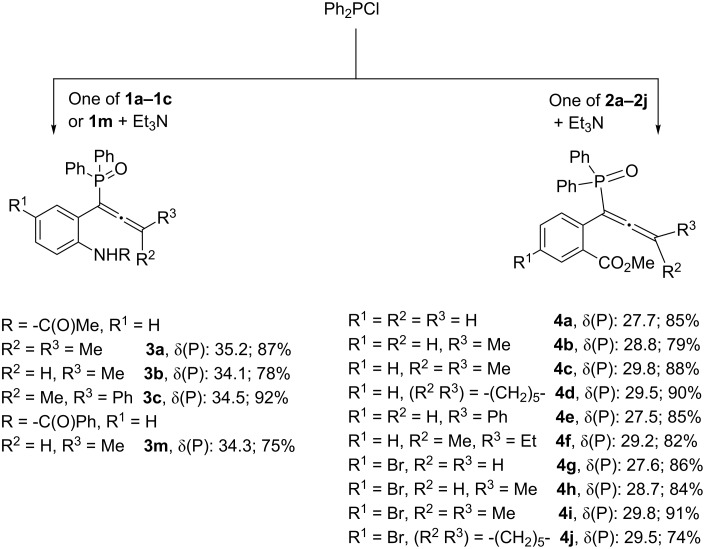
Synthesis of functionalized allenes **3a–c**, **3m** and **4a–j**.

In order to achieve the anticipated phosphinoylindoles/isocoumarins, we prepared a variety of functionalized propargyl alcohols **1a–m** and **2a–j** containing an acetamide, benzamide or an ester group at the *ortho* position (Figure 1) [35–37]. Some of the propargyl alcohols **1a–c**, **1m** and **2a–j** were transformed to allenylphosphine oxides **3a–c**, **3m** and **4a–j** (Scheme 2) by following known methods [38–39].

**Scheme 3:**
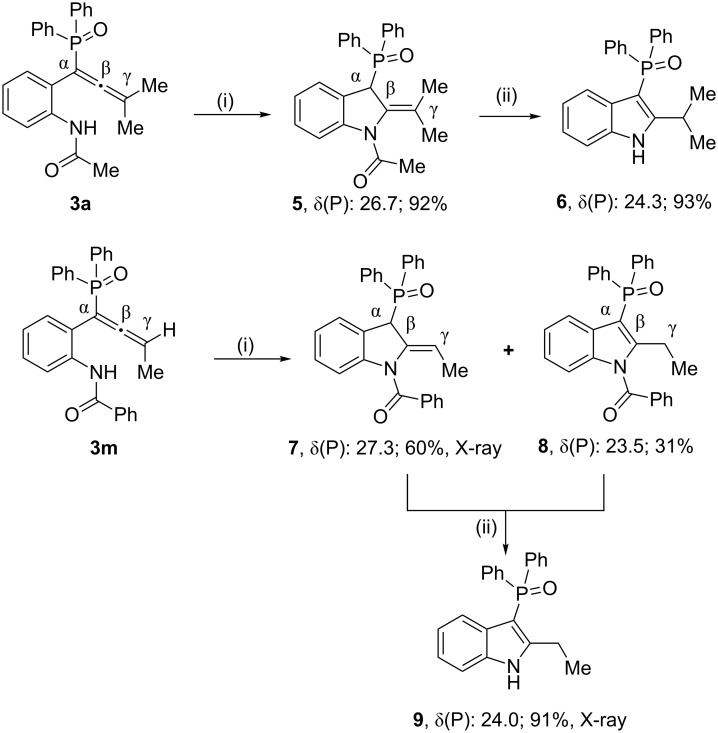
Reaction of functionalized allenes **3a** and **3m** leading to phosphinoylindoles. Conditions: (i) K_3_PO_4_ (0.5 equiv), THF, 80 °C, 12 h, (ii) NaOH (2 equiv), EtOH/H_2_O (4:1), 80 °C, 8 h.

**Figure 2:**
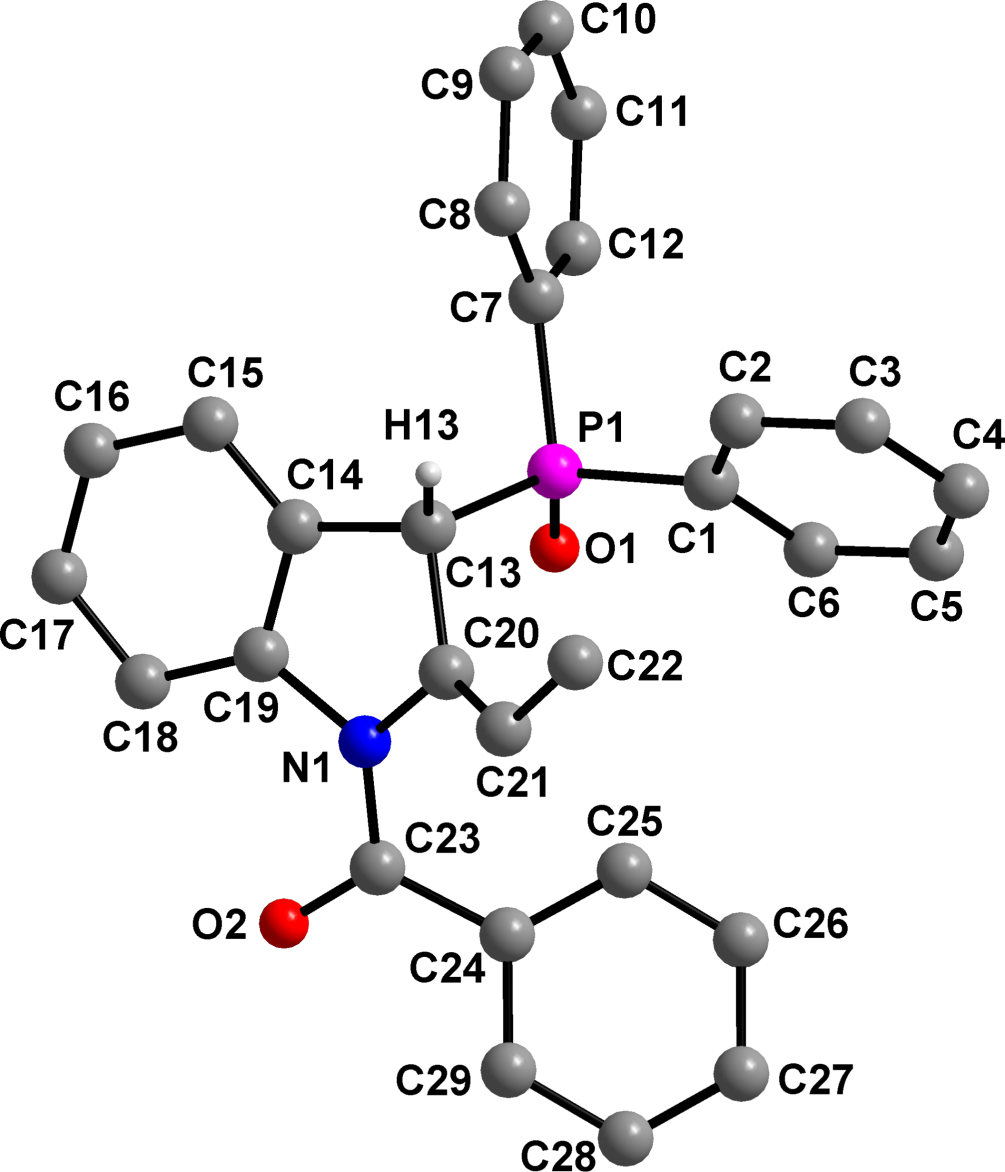
Molecular structure of compound **7**. Hydrogen atoms (except PCH) are omitted for clarity. Selected bond distances (Å): P1–C13 1.8402(15), C13–C20 1.5161(19), C13–C14 1.512(2), C20–N1 1.4508(18), C20–C21 1.317(2).

**Figure 3:**
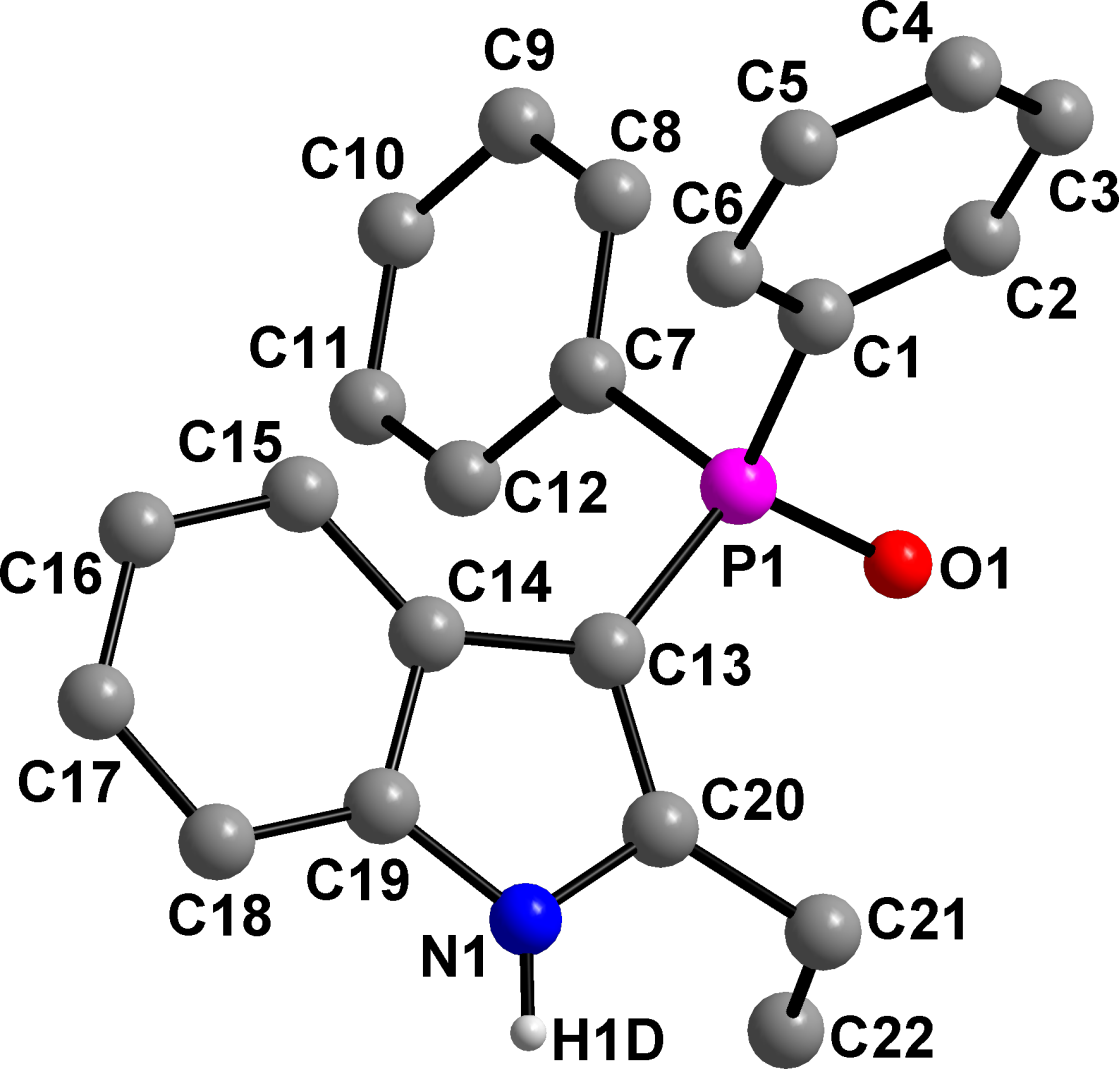
Molecular structure of compound **9**. Hydrogen atoms (except NH) are omitted for clarity. Selected bond distances (Å): P1–C13 1.771(2), C13–C20 1.385(3), C13–C14 1.450(3), C20–N1 1.360(3), C20–C21 1.489(3).

After having several functionalized allenes in hand, initially we chose allenes **3a** and **3m** to achieve intramolecular cyclization. These were treated with 0.5 mol equivalents of base (K_3_PO_4_) since the substrates contain active hydrogen. This reaction afforded the *N*-substituted phosphinoylindoles **5** and **7**, **8**. Essentially a single isomer **5** (a dihydroindole), in which the N–H proton moves only to the α-carbon resulting in an exocyclic double bond, was formed (Scheme 3). The presence of a doublet for P*C*H carbon at δ 48.3 with a ^1^*J*(P–C) value of 62.0 Hz reveals that the phosphorus moiety is attached to an sp^3^-hybridized carbon. On the other hand, in the reaction using the =CHMe allene **3m**, two isomers in which the N–H proton moves to either the α-carbon (**7**) or the γ-carbon (**8**), are obtained. These two isomers can be readily distinguished by the corresponding δ and ^1^*J* values for the P–C carbon (for **7**, δ 47.3 and *J* = 62.0 Hz; for **8**, δ 106.5 and *J* = 120.0 Hz). Overall, the yields of the isolated products were excellent in both cases. The structure of compound **7** was further confirmed by X-ray crystallography (Figure 2). The *C*=*C*HMe distance of 1.317(2) Å clearly indicates a double bond between these two carbon atoms. The other stereoisomer in which the methyl group is *trans* to the nitrogen was not observed. Interestingly though, the removal of the acyl/benzoyl group on the nitrogen in compounds **5** or **7**, **8** in aq NaOH afforded the 2,3-disubstituted N*H*-indoles **6** or **9**, respectively, in excellent yields. The NH band (3156 cm^−1^) in the IR spectrum and a doublet for PC carbon at δ 98.4 (^1^*J*(PC) = 128.0 Hz) reveal the identity of compound **9**. Its structure was further confirmed by X-ray crystallography (Figure 3).

**Scheme 4:**
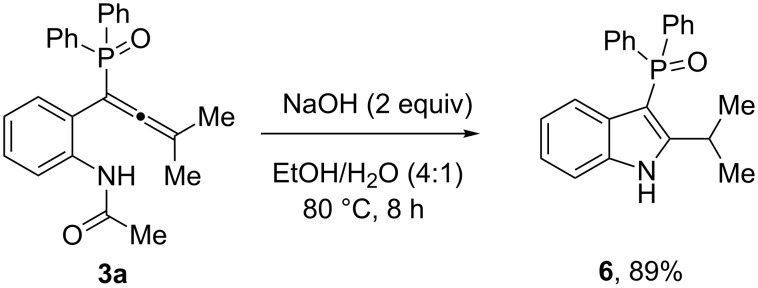
Synthesis of phosphinoylindole from allene **3a** in a single step.

Subsequently, we used aq sodium hydroxide as the base instead of K_3_PO_4_ (cf. conditions (ii) in Scheme 3) to perform the reaction on allene **3a**. To our delight, only phosphinoyl-N*H*-indole **6** was the sole product with not even traces of **5** (Scheme 4). This shows that a strong base like sodium hydroxide effectively performs both deprotection and cyclization in a single step.

**Scheme 5:**
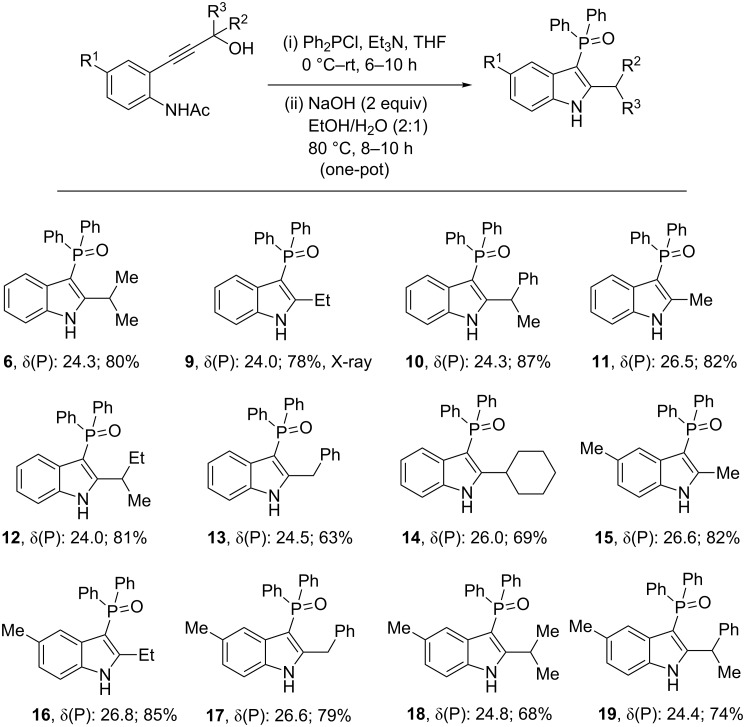
One-pot preparation of substituted phosphinoylindoles **6** and **9–19** from functionalized alcohols.

With the above conditions in hand, we then performed the reaction in one pot starting from propargyl alcohol **1a** without isolating the intermediate allenylphosphine oxide **3a**. Gratifyingly, the method furnished the desired product **6** in 80% yield. Inspired by this, functionalized propargyl alcohols **1b–l** were also subjected to the same one-pot conditions (Scheme 5). This one-pot strategy furnished the desired phosphinoylindoles **9–19** in good to excellent yields without any difficulty in isolation. Analogous products could also be isolated using the P(III) precursor (OCH_2_CMe_2_CH_2_O)PCl (see Supporting Information File 1 for details). In our attmept to obtain phosphorus-free 2-alkylindole from **17** in the presence of triflic acid (as a solvent; 100 °C) led to a mixture of products in which the benzyl group also was cleaved (NMR evidence). Such a reductive cleavage of the P–C bond from phosphinoyl indoles is a reaction that we are still exploring.

**Scheme 6:**
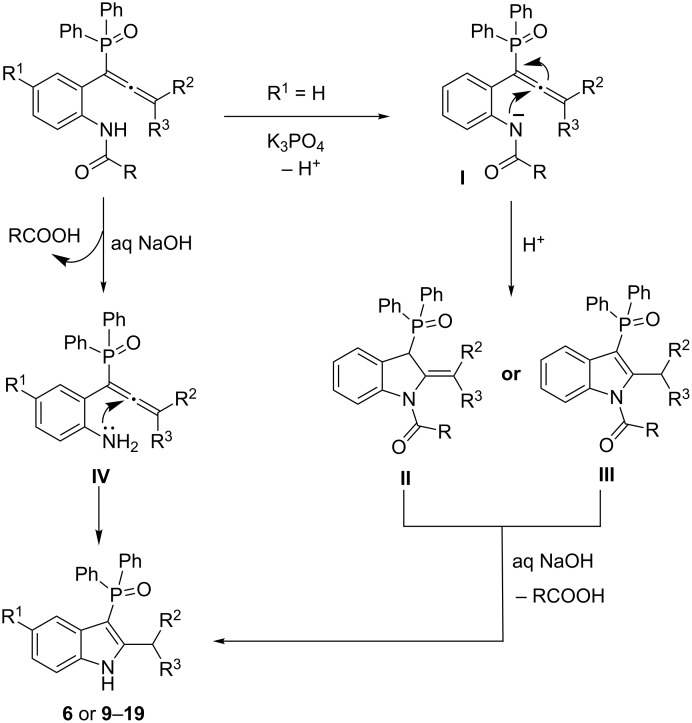
Possible pathway for the formation of phosphinoyl indoles **6** and **9–19**.

A plausible pathway for the formation of phosphinoylindoles **6** and **9–19** is shown in Scheme 6. As depicted above in Scheme 2, the normal reaction of propargyl alcohol with chlorodiphenylphosphine is expected to lead to the allenylphosphine oxide. We believe that there is a subtle difference between the use of K_3_PO_4_ and aq NaOH. K_3_PO_4_ abstracts the NH proton from allenylphosphine oxide leading to intermediate **I** which is followed by attack of the nitrogen lone pair on the β-carbon [24] of the allene forming addition product **II** or **III**. This upon treating with aq NaOH leads to the deacylated/debenzoylated phosphinoylindoles. In the one-pot reaction, though, the in situ generated allenylphosphine oxide first undergoes deacylation/debenzoylation with aq NaOH resulting in –NH_2_ functionalized allene **IV**; the lone pair on nitrogen will then attack the β-carbon of the allene intramolecularly leading to phosphinoylindoles **6** or **9–19**.

**Scheme 7:**
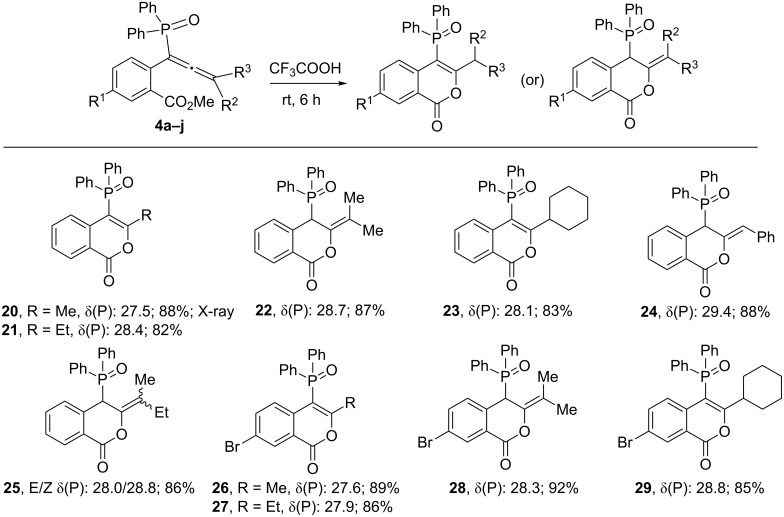
Synthesis of phosphinoylisocoumarins from functionalized allenes.

**Figure 4:**
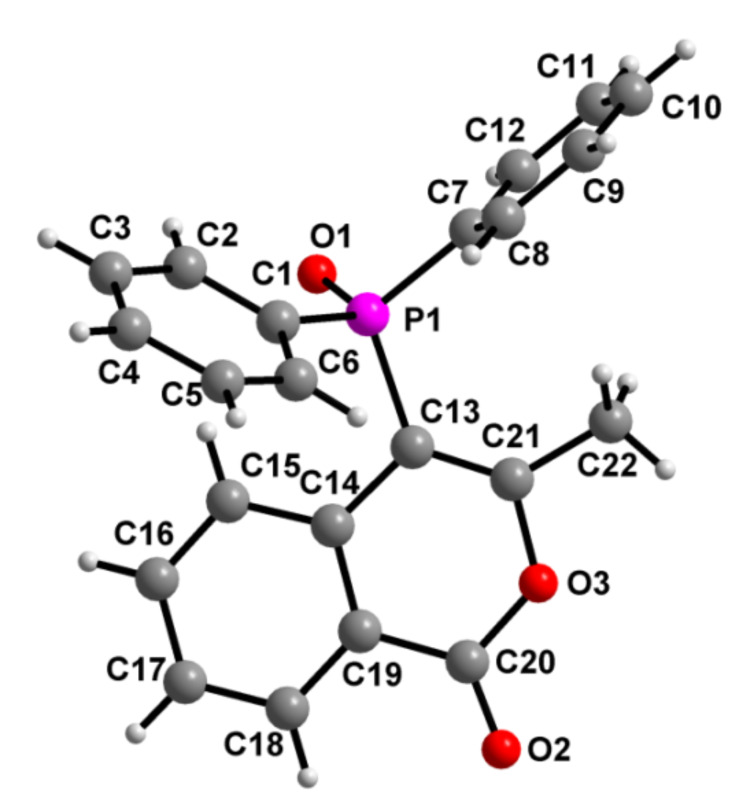
Molecular structure of **20**. Selected bond lengths [Å] with estimated standard deviations are given in parentheses: O3–C21 1.386(2), C21–C22 1.486(3).

After succeeding in generating phosphinoylindoles, we then concentrated on synthesizing phosphinoylisocoumarins. To achieve this, we treated the functionalized allene precursors **4a–j** that are tethered with a methyl ester group, with an excess of trifluoroacetic acid at room temperature for 6 h. Gratifyingly, this readily leds to the phosphinoylisocoumarins **20–29** (Scheme 7) in good yields. In the case of compound **25**, as expected, both the *E* and *Z* isomers are present in a ratio of 1:0.65 (close *R*_f_ values). Very subtle energy differences seem to be prevalent between the dihydroisocoumains **22**, **24, 25**, **28**, **29** and the normal isocoumarins **20**, **21**, **23**, **26, 27**. The former set shows a doublet in the ^1^H NMR spectra at δ ~ 4.78 (^2^*J*(P–H) = 18.0 Hz, PC*H*) which is absent in the latter set; the difference in the value of ^1^*J*(P–C) in the two sets is also consistent with the hybridization at the corresponding α-carbon (to phosphorus). Finally, the X-ray structure was determined for **20** (Figure 4).

**Scheme 8:**
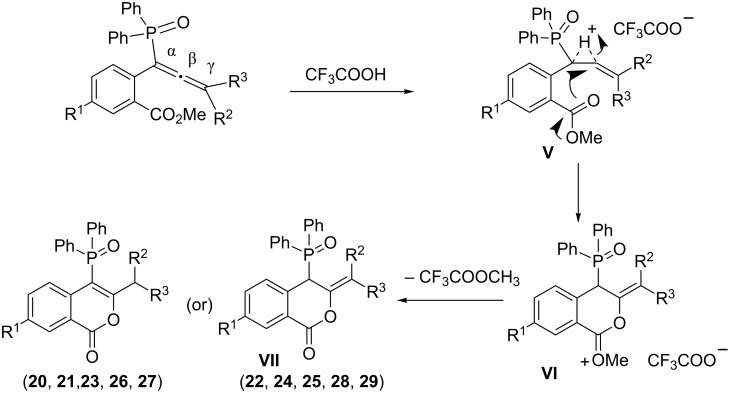
Possible pathway for the formation of phosphinoylisocoumarins.

The above reaction is believed to proceed by the initial interaction of H^+^ with the α,β-allenic double bond to lead to **V** (Scheme 8) which on subsequent attack of oxygen of the ester group onto the β-position of allene forms **VI**. Intermediate **VI** on demethylation leads to phosphinoylisocoumarin **VII**. This product **VII** further involves the double bond isomerization to lead to phosphinoyl isocoumarins **20**, **21**, **23**, **26** and **27**. The isomerization is not observed in the case of **22**, **24**, **25**, **28** and **29**. Alternatively, the cyclization may also proceed after the hydrolysis of ester group to –COOH due to the presence of adventitious moisture in trifluoroacetic acid.

**Scheme 9:**
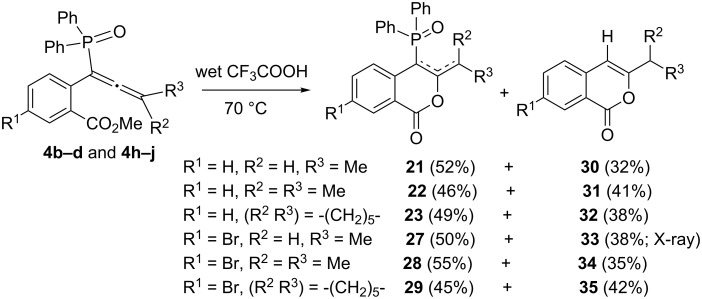
Reaction of allenes in wet trifluoroacetic acid.

**Figure 5:**
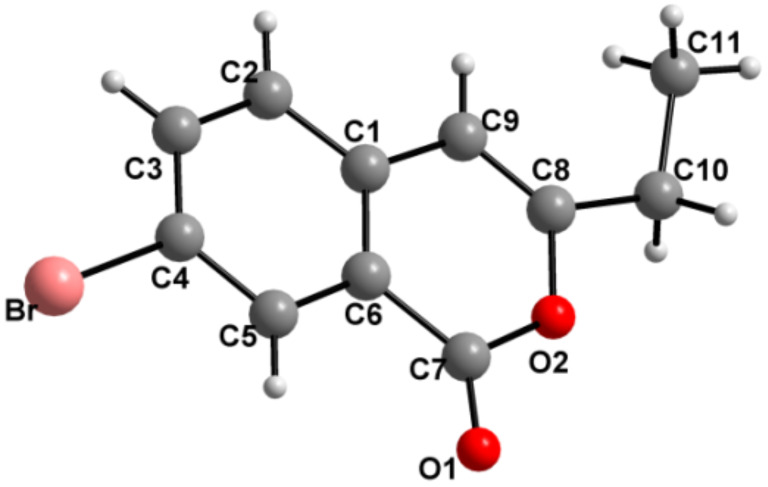
Molecular structure of **33**. Selected bond lengths [Å] with estimated standard deviations are given in parentheses: O2–C8 1.377(6), C8–C10 1.492(6).

**Scheme 10:**
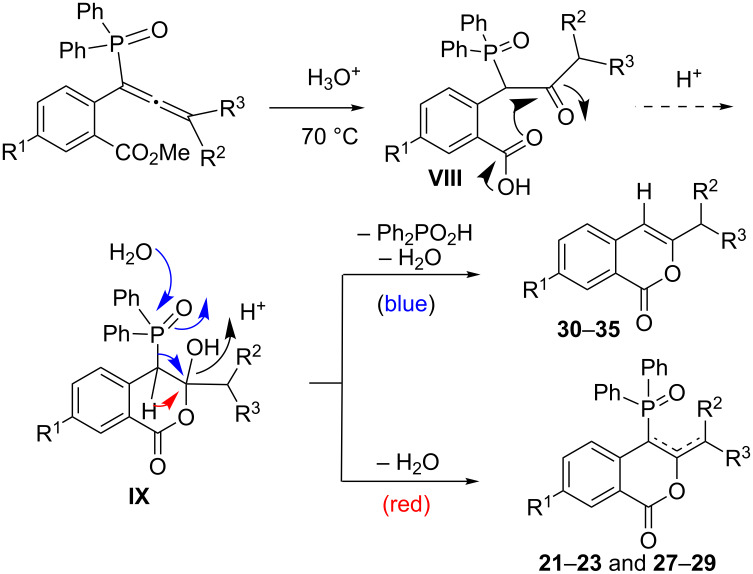
Possible pathway for the formation of isocoumarins **30–35** (along with **21–23** and **27–29**).

When the above reaction was performed in wet trifluoroacetic acid (TFA/H_2_O = 20:1) at 70 °C, phosphinoylisocoumarins were formed in all cases, but additionally, phosphorus-free isocoumarins **30–35** (Scheme 9) [37] are also formed in the reaction using terminally substituted allenes **4b–d** and **4h–j**. We have also determined the X-ray structure of compound **33** (Figure 5) for final confirmation. It is possible that isocoumarins **30–35** are formed via the intermediates **VIII–IX** (Scheme 10) [40]. The phosphorus moiety of **IX** may then be cleaved as Ph_2_POOH to form the phosphorus-free isocoumarins. Since this was not the interest in the present study, we did not proceed further.

## Conclusion

A fairly simple route to phosphinoylindoles and phosphinoylisocoumarins starting from functionalized propargyl alcohols via allenyl phosphine oxide is developed. The first reaction involves base-mediated deprotection and cyclization while the latter methodology involves acid mediation in which trifluoroacetic acid acts as the reagent as well as the solvent.

## Experimental

Details on the synthesis of the compounds **1a**–**1m**, **2a**–**2j**, **3a**–**3c**, **3m**, **4a**–**4j** and **5**–**35** are given in Supporing Information File 1.

Crystallographic data for the structures of **7**, **9**, **20** and **33** have been deposited with the Cambridge Crystallographic Data Centre as supplementary publication number CCDC 981067-981070. Copies of the data can be obtained free of charge, on application to CCDC, 12 Union Road, Cambridge CB2 1EZ, UK [fax: +44(1223)336033 or e-mail: deposit@ccdc.cam.ac.uk]. The structures were solved and refined by standard methods [41–43].

**7**: Colorless block, C_29_H_24_NO_2_P, *M* = 449.46, monoclinic, space group *P*2_1_/c, *a* = 9.9427(15), *b* = 16.894(3), *c* = 14.819(2) Å, α = 90.00, β = 109.195(2), γ = 90.00^o^, *V* = 2350.8(6) Å^3^_,_ *Z* = 4, µ = 0.143 mm^−1^, data/restrains/parameters: 4141/0/299, R indices (I> 2σ(I)): R1 = 0.0408, *w*R2 (all data) = 0.1101. CCDC no. 981067.

**9**: Colorless block, C_22_H_20_NOP, *M* = 345.36, orthorhombic, space group *Pccn*, *a* = 11.2497(6), *b* = 21.1287(9), *c* = 15.2880(6) Å, α = 90, β = 90, γ = 90^o^, *V* = 3633.8(3) Å^3^_,_ *Z* = 8, µ = 0.160 mm^−1^, data/restrains/parameters: 3205/0/231, R indices (I> 2σ(I)): R1 = 0.0430, *w*R2 (all data) = 0.1076. CCDC no. 981068.

**20**: Colorless block, C_22_H_17_O_3_P, *M* = 360.33, triclinic, space group 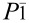, *a* = 9.7440(19), *b* = 9.9918(17), *c* = 10.2864(18) Å, α = 84.229(14), β = 76.556(16), γ = 66.323(18)^o^, *V* = 892.0(3) Å^3^_,_ *Z* = 2, µ = 0.173 mm^−1^, data/restrains/parameters: 3647/0/236, R indices (I> 2σ(I)): R1 = 0.0455, *w*R2 (all data) = 0.1114. CCDC no. 981069.

**33**: Colorless needles, C_11_H_9_BrO_2_, *M* = 253.09, triclinic, space group 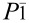, *a* = 7.9413(19), *b* = 7.9674(19), *c* = 9.746(2) Å, α = 66.05(2), β = 79.379(19), γ = 62.93(2)^o^, *V* = 501.8(2) Å^3^_,_ *Z* = 2, µ = 4.064 mm^−1^, data/restrains/parameters: 1354/0/128, R indices (I> 2σ(I)): R1 = 0.0425, *w*R2 (all data) = 0.1021. CCDC no. 981070.

## Supporting Information

File 1 Details on the synthesis and characterization of the compounds **1a**–**1m**, **2a**–**2j**, **3a**–**3c**, **3m**, **4a**–**4j** and **5**–**35** and ^1^H/^13^C NMR spectra of new compounds (including **A**–**B**).

File 2 CIF file for the compounds **7**, **9**, **20** and **33**.
