# Supplementary material for: Allenylphosphine oxides as simple scaffolds for phosphinoylindoles and phosphinoylisocoumarins
Source: Beilstein J Org Chem. 2014 May 2;10:996–1005. doi: 10.3762/bjoc.10.99 (PMC4077435; doi:10.3762/bjoc.10.99)
Supplement: File 1 — Details on the synthesis and characterization of the compounds 1a–1m, 2a–2j, 3a–3c, 3m, 4a–4j and 5–35 and 1H/13C NMR spectra of new compounds (including A–B). [file Beilstein_J_Org_Chem-10-996-s001.pdf]

Supporting Information

for

Allenylphosphine oxides as simple scaffolds for phosphinoylindoles and phosphinoylisocoumarins

G. Gangadhararao, Ramesh Kotikalapudi, M. Nagarjuna Reddy and K. C. Kumara Swamy*

Address: School of Chemistry, University of Hyderabad, Hyderabad 500 046, A. P., India. Fax: (+91)-40-23012460

Email: K. C. Kumara Swamy - kckssc@uohyd.ac.in

* Corresponding author

Details on the synthesis and characterization of the compounds 1a–1m, 2a–2j, 3a–3c, 3m, 4a–4j and 5–35 and $^{1}H/^{13}C$ NMR spectra of new compounds (including A–B)

Contents

General information	S2
Preparation and characterization of compounds	S2–S33
References	S33–S34
^{1}H and ^{13}C NMR spectra for all compounds	S34–S98

General experimental details

Chemicals were purified when required according to standard procedures [1]. All reactions, unless stated otherwise, were performed in a dry nitrogen atmosphere. ^{1}H, ^{13}C and ^{31}P NMR spectra were recorded using a 400 MHz spectrometer in $CDCl_3$ (unless stated otherwise) with shifts referenced to $SiMe_4$ (δ = 0) or 85% H_3PO_4 (δ = 0). Infrared spectra were recorded neat or by using KBr pellets on an FTIR spectrometer. Melting points were determined by using a local hot-stage melting point apparatus and are uncorrected. Microanalyses were performed using a CHNS analyzer. For TLC, glass microslides were coated with silica gel GF_{254} (mesh size 75 μ) and spots were identified using iodine or UV chamber as appropriate. For column chromatography, silica gel of 100–200 mesh size was used. LCMS and HRMS equipment was used to record mass spectra for isolated compounds where appropriate. LCMS data were obtained using electrospray ionization (positive mode) on a C-18 column at a flow rate 0.2 mL/ min using MeOH/water (90:10) as eluent. Chlorodiphenylphosphine (Ph_2PCl), procured from Aldrich, was distilled prior to use. 4-methyl-2-iodo aniline [2], *N*-(2-iodophenyl)acetamide [3] and *N*-(2-iodophenyl)benzamide [4] were prepared according to the literature methods. -Methyl 2-iodobenzoate [5] and methyl 5-bromo-2-iodobenzoate [6] were prepared by esterification of the corresponding carboxylic acid with methanol.

(a) Experimental procedure for the synthesis of *N*-acetylated propargyl alcohols

In a round-bottomed flask (50 mL) equipped with *N*-(2-iodophenyl)acetamide [3] (0.8 g, 3.06 mmol), $Pd(PPh_3)_2Cl_2$ (0.05 g, 0.08 mmol), CuI (0.03 g, 0.15 mmol) and acetonitrile (20 mL), was added 2-methyl-3-butyne-2-ol (0.33 mL, 3.37 mmol) and Et_3N (1.71 mL, 12.25 mmol). The mixture was stirred at room temperature for 6–

8 h. Upon completion of the reaction (TLC), the mixture was filtered, the solid residue washed with EtOAc and washings added to the filtrate. The combined solution was concentrated under reduced pressure. The oily material obtained was subjected to column chromatography (hexane/ethyl acetate 4:1) to afford the desired product **1a**. Propargyl alcohols **1b–1m** were prepared by using the same experimental procedure. Among these compounds, **1a** and **1d** are known.

Compound 1a: Light yellow solid; yield: 0.60 g, (90%). The spectral data are in accordance with the literature report [7].

Compound 1b: Brown gummy liquid; yield: 0.94 g (94%; using 1.0 g (3.80 mmol) of *N*-(2-iodophenyl)acetamide; ^{1}H NMR (400 MHz, $CDCl_3$) δ 1.47 (d, *J* = 6.4 Hz, 3H, CHC*H*$_3$), 2.06 (s, 3H, COC*H*$_3$), 4.71 (qrt, 1H, C*H*CH_3), 5.15 (br s, 1H, O*H*), 6.88 (t, 1H, Ar-*H*), 7.11 (t, 1H, Ar-*H*), 7.22 (d, *J* = 7.6 Hz, 1H, Ar-*H*), 8.04 (d, *J* = 8.0 Hz, 1H, Ar-*H*), 8.21 (s, 1H, N*H*). ^{13}C NMR (100 MHz, $CDCl_3$) δ 24.3 (CH*C*H$_3$), 58.2 (CO*C*H$_3$), 77.6 (*C*HCH_3), 78.9 (C≡*C*), 98.6 (*C*≡C), 112.7, 120.4, 123.8, 129.2, 131.6, 138.6 (Ar-*C*), 169.5 (*C*O). IR (neat, cm^{-1}) 3320, 2981, 2926, 2871, 2224, 1671, 1583, 1523, 1446, 1364, 1299, 1118, 1096, 1030, 931, 860. LC-MS: *m/z* 204 [M+1]$^+$. Anal. Calcd. for $C_{12}H_{13}NO_2$: C, 70.92; H, 6.45; N, 6.89. Found: C, 70.85; H, 6.53; N, 6.76.

Compound 1c: Brown solid; yield: 1.91 g, (89%; using 2.0 g (7.66 mmol) of *N*-(2-iodophenyl)acetamide; mp 94-96 ^{o}C; ^{1}H NMR (400 MHz, $CDCl_3$) δ 1.91 and 1.95 (2 s, 6H, C*H*$_3$ + COC*H*$_3$), 3.78 (br s, 1H, O*H*), 7.02 (t, 1H, Ar-*H*), 7.25-7.41 (m, 5H, Ar-*H*), 7.71 (d, *J* = 7.2 Hz, 2H, Ar-*H*), 7.88 (br s, 1H, N*H*), 8.26 (d, *J* = 8.4 Hz, 1H, Ar-*H*). ^{13}C NMR (100 MHz, $CDCl_3$) δ 24.6 and 33.1 (2 s, *C*H$_3$ + CO*C*H$_3$), 70.2 (*C*OH), 79.8 (C≡*C*), 100.6 (*C*≡C), 119.6, 123.5, 124.8, 124.9, 127.9, 128.4, 128.6, 129.9, 131.6, 139.2, 145.6 (Ar-*C*), 168.8 (*C*O). IR (KBr, cm^{-1}) 3342, 3255, 2986, 2926, 2175, 1633,

1540, 1423, 1375, 1304, 1096, 1052, 899, 762. HRMS (ESI) Calcd. for $C_{18}H_{17}NO_2$ $(M + Na)^+$: 302.1157. Found: 302.1156.

Compound 1d: Light yellow solid; yield: 0.54 g, (92%; using 0.8 g (3.06 mmol) of *N*-(2-iodophenyl)acetamide; The spectral data are in accordance with the literature report [8].

Compound 1e: Brown gummy liquid; yield: 0.63 g, (89%; using 0.8 g (3.06 mmol) of *N*-(2-iodophenyl)acetamide; ^{1}H NMR (400 MHz, $CDCl_3$) δ 1.12 (t, 3H, CH_2CH_3), 1.60 (s, 3H, CCH_3), 1.78-1.85 (m, 2H, CH_2CH_3), 2.16 (s, 3H, $COCH_3$), 3.16 (br s, 1H, O*H*), 6.99 (t, 1H, Ar-*H*), 7.24-7.28 (m, 1H, Ar-H), 7.34 (d, *J* = 6.8 Hz, 1H, Ar-*H*), 7.99 (br s, 1H, N*H*), 8.28 (d, *J* = 7.6 Hz, 1H, Ar-*H*). ^{13}C NMR (100 MHz, $CDCl_3$) δ 9.3 (CH_2CH_3), 24.7 ($COCH_3$), 29.5 (CH_2CH_3), 36.6 (CH_3), 69.2 (*C*OH), 78.6 (C≡*C*), 100.4 (*C*≡C), 111.8, 119.6, 123.5, 129.6, 131.6, 138.9 (Ar-*C*), 168.6 (*C*O). IR (neat, cm^{-1}) 3381, 3293, 2975, 2932, 2225, 1677, 1578, 1518, 1447, 1381, 1310, 1156, 1003, 762. LC-MS: *m/z* 232 $[M+1]^+$. Anal. Calcd. for $C_{14}H_{17}NO_2$: C, 72.70; H, 7.41; N, 6.06. Found: C, 72.83; H,7.36; N, 6.15.

Compound 1f: Brown solid; yield: 0.46 g, (57%; using 0.8 g (3.06 mmol) of *N*-(2-iodophenyl)acetamide; mp 92-94 ^oC; ^{1}H NMR (400 MHz, $CDCl_3$) δ 1.98 (s, 3H, $COCH_3$), 3.69 (br s, 1H, O*H*), 5.73 (s, 1H, C*H*OH), 7.01 (t, 1H, Ar-*H*), 7.30 (d, *J* = 7.6 Hz, 1H, Ar-*H*), 7.33-7.42 (m, 4H, Ar-*H*), 7.59 (d, *J* = 7.6 Hz, 2H, Ar-*H*), 7.85 (s, 1H, N*H*), 8.27 (d, *J* = 8.4 Hz, 1H, Ar-*H*). ^{13}C NMR (100 MHz, $CDCl_3$) δ 24.6 ($COCH_3$), 64.9 (*C*HOH), 81.7 (C≡*C*), 96.8 (*C*≡C), 111.4, 119.7, 123.5, 126.4, 128.6, 128.9, 130.0, 131.7, 139.2, 140.9 (Ar-*C*), 168.8 (*C*O). IR (KBr, cm^{-1}) 3337, 3254, 3052, 3030, 2203, 1633, 1573, 1529, 1447, 1299, 1184, 1025, 970, 767. LC-MS: *m/z* 266

[M+1]$^+$. Anal. Calcd. for $C_{17}H_{15}NO_2$: C, 76.96; H, 5.70; N, 5.28. Found: C, 76.85; H, 5.65; N, 5.32.

Compound 1g: Brown solid; yield: 0.67 g, (85%; using 0.8 g (3.06 mmol) of *N*-(2-iodophenyl)acetamide; mp 110-112 ^oC; ^1H NMR (400 MHz, $CDCl_3$) δ 1.30 (br s, 1H, cyclohexyl-*H*), 1.58-1.77 (m, 7H, cyclohexyl-*H*), 2.03 (d, *J* = 9.6 Hz, 2H, cyclohexyl-*H*), 2.15 (s, 3H, $COCH_3$), 3.49 (br s, 1H, O*H*), 6.98 (t, 1H, Ar-*H*), 7.23-7.27 (m, 1H, Ar-H), 7.33 (d, *J* = 7.6 Hz, 1H, Ar-*H*), 8.02 (br s, 1H, N*H*), 8.27 (d, *J* = 8.0 Hz, 1H, Ar-*H*). ^{13}C NMR (100 MHz, $CDCl_3$) δ 23.5, 24.7, 25.1 and 40.0 (4 s, CH_2+$COCH_3$), 69.1 (*C*OH), 79.4 (C≡*C*), 100.6 (*C*≡C), 111.9, 119.6, 123.5, 129.5, 131.5, 138.8 (Ar-*C*), 168.6 (*C*O). IR (KBr, cm^{-1}) 3408, 3381, 2948, 2926, 2860, 2214, 1676, 1578, 1528, 1451, 1309, 1265, 1161, 1068, 964, 755. LC-MS: *m/z* 258 [M+1]$^+$. Anal. Calcd. for $C_{16}H_{19}NO_2$: C, 74.68; H, 7.44; N, 5.44. Found: C, 74.57; H,7.51; N, 5.36.

Compound 1h: Brown solid; yield: 0.28 g, (75%; using 0.5 g (1.82 mmol) of *N*-(2-iodo-4-methylphenyl)acetamide; mp 104-106 ^oC; ^1H NMR (400 MHz, $CDCl_3$) δ 2.14 and 2.20 (2 s, 6H, Ar-CH_3 + $COCH_3$), 4.04 (br s, 1H, O*H*), 4.50 (d, *J* = 3.2 Hz, 2H, CH_2OH), 7.04 (t, 1H, Ar-*H*), 7.12 (s, 1H, Ar-*H*), 8.00-8.07 (m, 2H, N*H*+Ar-*H*). ^{13}C NMR (100 MHz, $CDCl_3$) δ 20.6 (Ar-*C*H3), 24.5 ($COCH_3$), 51.1 (CH_2), 80.8 (C≡*C*), 94.3 (*C*≡C), 112.3, 120.2, 130.3, 132.1, 133.4, 136.3 (Ar-*C*), 169.2 (*C*O). IR (KBr, cm^{-1}) 3332, 2921, 2855, 2247, 1671, 1589, 1518, 1458, 1414, 1315, 1129, 1030, 893, 827. LC-MS: *m/z* 204 [M+1]$^+$. Anal. Calcd. for $C_{12}H_{13}NO_2$: C, 70.92; H, 6.45; N, 6.89. Found: C, 70.85; H,6.52; N, 6.81.

Compound 1i: Brown gummy liquid; yield: 0.44 g, (92%; using 0.6 g (2.18 mmol) of *N*-(2-iodo-4-methylphenyl)acetamide; ^1H NMR (400 MHz, $CDCl_3$) δ 1.54 (d, *J* = 6.8 Hz, 3H, $CHCH_3$), 2.13 and 2.20 (2 s, 6H, Ar-CH_3 + $COCH_3$), 4.20 (br s, 1H, O*H*), 4.76

(qrt, 1H, C*H*CH_3), 7.02 (d, *J* = 8.4 Hz, 1H, Ar-*H*), 7.11 (s, 1H, Ar-*H*), 8.02 (d, *J* = 5.6 Hz, 1H, Ar-*H*), 8.05 (s, 1H, N*H*). ^{13}C NMR (100 MHz, $CDCl_3$) δ 20.6, 24.4 and 24.5 (3 s, *C*H_3), 58.4 (*C*HOH), 79.2 (C≡*C*), 98.2 (*C*≡C), 112.2, 120.1, 130.2, 131.9, 133.3, 136.3 (Ar-*C*), 169.0 (*C*O). IR (neat, cm^{-1}) 3359, 3271, 2975, 2926, 2855, 2219, 1633, 1578, 1529, 1403, 1364, 1310, 1107, 1041, 970, 811. LC-MS: *m/z* 218 $[M+1]^+$. Anal. Calcd. for $C_{13}H_{15}NO_2$: C, 71.87; H, 6.96; N, 6.45. Found: C, 71.96; H, 7.02; N, 6.38.

Compound 1j: Brown gummy liquid; yield: 0.52 g, (85%; using 0.6 g (2.18 mmol) of *N*-(2-iodo-4-methylphenyl)acetamide; ^{1}H NMR (400 MHz, $CDCl_3$) δ 1.95 and 2.24 (2 s, 6H, Ar-CH_3 + COCH_3), 4.22 (br s, 1H, O*H*), 5.71 (s, 1H, C*H*OH), 7.05 (d, *J* = 8.4 Hz, 1H, Ar-*H*), 7.17 (s, 1H, Ar-*H*), 7.33-7.40 (m, 3H, Ar-*H*), 7.58 (d, *J* = 7.2 Hz, 1H, Ar-*H*), 7.84 (s, 1H, N*H*), 8.08 (d, *J* = 8.4 Hz, 1H, Ar-*H*). ^{13}C NMR (100 MHz, $CDCl_3$) δ 20.6 (Ar-*C*H_3), 24.4 (CO*C*H_3), 64.8 (*C*HOH), 81.8 (C≡*C*), 96.4 (*C*≡C), 111.7, 119.9, 126.4, 128.5, 128.8, 130.6, 131.9, 133.2, 136.7, 141.0 (Ar-*C*), 168.9 (*C*O); IR (neat, cm^{-1}) 3386, 3293, 3030, 2921, 2230, 1666, 1595, 1518, 1452, 1370, 1304, 1134, 1030, 822, 701. LC-MS: *m/z* 280 $[M+1]^+$. Anal. Calcd. for $C_{18}H_{17}NO_2$: C, 77.40; H, 6.13; N, 5.01. Found: C, 77.32; H, 6.07; N, 5.12.

Compound 1k: Brown solid; yield: 0.37 g, (90%; using 0.49 g (1.53 mmol) of *N*-(2-iodo-4-methylphenyl)acetamide; mp 100-102 ^{o}C; ^{1}H NMR (400 MHz, $CDCl_3$) δ 1.62 (s, 6H, C(CH_3)$_2$), 2.19 and 2.20 (2 s, 6H, Ar-CH_3 + COCH_3), 3.96 (br s, 1H, O*H*), 7.01 (d, *J* = 8.4 Hz, 1H, Ar-*H*), 7.10 (s, 1H, Ar-*H*), 7.96 (br s, 1H, N*H*), 8.06 (d, *J* = 8.4 Hz, 1H, Ar-*H*). ^{13}C NMR (100 MHz, $CDCl_3$) δ 20.6 and 24.5 (2 s, *C*H_3), 31.5 (C*C*H_3), 65.4 (*C*OH), 77.4 (C≡*C*), 101.2 (*C*≡C), 112.1, 119.9, 130.1, 131.7, 133.1, 136.4 (Ar-*C*), 168.7 (*C*O). IR (KBr, cm^{-1}) 3386, 3364, 2981, 2926, 2194, 1682, 1600, 1518, 1458, 1403, 1310, 1173, 1140, 964, 877, 827. LC-MS: *m/z* 232 $[M+1]^+$. Anal. Calcd. for $C_{14}H_{17}NO_2$: C, 72.70; H, 7.41; N, 6.06. Found: C, 72.61; H,7.36; N, 6.13.

Compound 1l: Brown gummy liquid; yield: 0.40 g, (93%; using 0.4 g (1.45 mmol) of *N*-(2-iodo-4-methylphenyl)acetamide; ^{1}H NMR (400 MHz, $CDCl_3$) δ 1.89 (s, 3H, CCH_3), 1.93 and 2.25 (2 s, 6H, Ar-CH_3 + $COCH_3$), 4.06 (br s, 1H, O*H*), 7.05 (d, *J* = 8.0 Hz, 1H, Ar-*H*), 7.19 (s, 1H, Ar-*H*), 7.29-7.32 (m, 1H, Ar-*H*), 7.37 (dd→t, 1H, Ar-*H*), 7.70 (d, *J* = 7.6 Hz, 2H, Ar-*H*), 7.83 (br s, 1H, N*H*), 8.09 (d, *J* = 8.4 Hz, 1H, Ar-*H*). ^{13}C NMR (100 MHz, $CDCl_3$) δ 20.6, 24.4 and 33.2 (3 s, CH_3), 70.1 (*C*HOH), 79.9 (C≡*C*), 100.2 (*C*≡C), 111.7, 119.8, 124.8, 127.8, 128.5, 130.5, 131.8, 133.1, 136.7, 145.8 (Ar-*C*), 168.7 (*C*O). IR (neat, cm^{-1}) 3375, 3299, 3030, 2975, 2926, 2219, 1677, 1595, 1447, 1370, 1304, 1184, 1140, 822, 762. LC-MS: *m/z* 294 $[M+1]^+$. Anal. Calcd. for $C_{19}H_{19}NO_2$: C, 77.79; H, 6.53; N, 4.77. Found: C, 77.82; H, 6.61; N, 4.68.

Compound 1m: Brown solid; yield: 0.9 g, (90%; using 1.0 g (3.10 mmol) of *N*-(2-iodophenyl)benzamide [4]; mp 84-86 ^{o}C; ^{1}H NMR (400 MHz, $CDCl_3$) δ 1.54 (d, *J* = 6.8 Hz, 3H, $CHCH_3$), 4.03 (br s, 1H, O*H*), 4.80 (qrt, 1H, C*H*CH_3), 6.96 (t, 1H, Ar-*H*), 7.23-7.42 (m, 5H, Ar-*H*), 7.84 (d, *J* = 7.6 Hz, 2H, Ar-*H*), 8.46 (d, *J* = 8.4 Hz, 1H, Ar-*H*), 8.75 (s, 1H, N*H*). ^{13}C NMR (100 MHz, $CDCl_3$) δ 24.3 (CH_3), 58.3 (*C*HCH_3), 78.7 (C≡*C*), 99.5 (*C*≡C), 111.9, 119.1, 123.5, 127.0, 128.7, 129.6, 131.4, 132.0, 134.1, 138.8 (Ar-*C*), 165.3 (*C*O). IR (KBr, cm^{-1}) 3386, 3063, 2975, 2926, 2860, 2218, 1666, 1578, 1523, 1446, 1304, 1260, 1107, 1030, 937, 794. LC-MS: *m/z* 266 $[M+1]^+$. Anal. Calcd. for $C_{17}H_{15}NO_2$: C, 76.96; H, 5.70; N, 5.28. Found: C, 76.85; H, 5.62; N, 5.37.

(b) Experimental procedure for the preparation of propargylic precursors 2a–2j

Propargylic precursors **2a**–**2j** were prepared by Sonogashira reaction of aryl halides with appropriate terminal propargyl alcohols by following a literature

procedure [9]. Among these propargyl alcohols **2d**–**2f** are new. The general procedure for the synthesis of these compounds is given below.

Compound 2a: To a stirred solution of aryl iodide (2.62 g, 10 mmol), $PdCl_2$ (0.018 g, 0.1 mmol), PPh_3 (0.053 g, 0.2 mmol) and CuI (0.039 g, 0.2 mmol) in triethylamine (15 mL) was added propargyl alcohol (1.07 g, 20 mmol) at rt (25 ^{o}C). The contents were stirred at reflux for 10 h. After all the starting material was consumed (TLC), the reaction mixture was filtered and the solvent removed by vacuum. The product **2a** was purified by column chromatography using silica gel with ethyl acetate/hexane mixture (1:4) as the eluent. yield: 1.67 g (88%). The spectral data are in accordance with the literature report [10]. Propargyl alcohols **2b–2f**were prepared using same molar quantities. Among these compounds **2a–2c** are known and **2d–2f** are new.

Compound 2b: Yield: 1.65 g (81%). The spectral data are in accordance with the literature report [11].

Compound 2c: Yield: 2.00 g (92%). The spectral data are in accordance with the literature report [11].

Compound 2d: Gummy liquid; yield: 2.17 g, 84%; ^{1}H NMR (400 MHz, $CDCl_3$) δ1.55-2.06 (m, 10H, cyclohexyl-*H*), 3.42 (br s, 1H, C-O*H*), 3.87 (s, 3H, COOCH_3), 7.31-7.89 (m, 4H, Ar*H*); ^{13}C NMR (100 MHz, $CDCl_3$) δ 23.4, 25.3 and 39.9 (cyclohexyl-*C*), 52.2 (COO*C*H_3), 69.1 (*C*-OH), 82.8 and 98.4 (*C*≡*C*), 123.5, 127.8, 130.3, 131.6, 131.8 and 134.2 (Ar-*C*), 166.8 (*C*=O); IR (neat, cm^{-1}) 2932, 2857, 1730, 1717, 1447, 1254, 1134, 1082, 965, 758; LC/MS *m/z* 259 $[M+1]^{+}$; Anal.Calcd. for $C_{16}H_{18}O_3$: C, 74.39; H, 7.02. Found: C, 74.61; H, 6.93.

Compound 2e: Gummy liquid; yield: 2.23 g, 84%; ^{1}H NMR (400 MHz, $CDCl_3$) δ 3.39 (br s, 1H, O*H*), 3.82 (s, 3H, COOCH_3), 5.75 (s, 1H, C*H*OH), 7.32-7.57 (m, 8H, Ar*H*), 7.66 (d, *J* = 7.2 Hz, 1H, Ar*H*); ^{13}C NMR (100 MHz, $CDCl_3$) δ 52.2 (COO*C*H_3), 65.1 (*C*H-OH), 85.1 and 94.2 (*C*≡*C*), 123.1, 126.9, 128.2, 128.3, 128.6, 130.4, 131.8, 131.9, 134.2 and 140.7 (Ar-*C*), 166.7 (*C*=O); IR (neat, cm^{-1}) 3419, 2954, 2203, 1715, 1490, 1447, 1293, 1260, 1189, 1079, 964, 762; LC/MS *m/z* 265 $[M-1]^+$; Anal.Calcd. for $C_{17}H_{14}O_3$: C, 76.68; H, 5.30.Found: C, 76.58; H, 5. 38.

Compound 2f: Gummy liquid; yield: 1.76 g, 76%; ^{1}H NMR (400MHz, $CDCl_3$) δ 1.12 (t, *J* ~ 7.2 Hz, 3H, $CH_2C$$H_3$), 1.58 (s, 3H, C$H_3$), 1.79-1.83 (m, 2H, C$H_2$$CH_3$), 3.89 (s, 3H, COOC$H_3$), 7.33-7.52 (m, 3H, Ar*H*), 7.90 (d, *J* = 8.0 Hz, 1H, Ar*H*); ^{13}C NMR (100 MHz, $CDCl_3$) δ 9.1, 29.1, 36.5, 52.2 (COO*C*H_3), 69.2 (*C*H-OH), 82.1 and 98.0 (*C*≡*C*), 123.3, 127.9, 130.1, 131.6, 132.0 and 134.0 (Ar-*C*), 166.7 (*C*=O); IR (neat, cm^{-1}) 3419, 2954, 2203, 1715, 1490, 1447, 1293, 1260, 1189, 1079, 964, 762; LC/MS *m/z* 233 $[M+1]^+$; Anal.Calcd. for $C_{14}H_{16}O_3$: C, 72.39; H, 6.94. Found: C, 72.29; H, 7.06.

Compound 2g: Gummy solid; yield: 1.05 g, 78%; ^{1}H NMR (400 MHz, $CDCl_3$) δ 3.91 (s, 3H, COOCH_3), 4.53 (s, 2H, CH_2OH), 7.37-8.06 (m, 3H, Ar*H*); ^{13}C NMR (100 MHz, $CDCl_3$)δ51.7 (*C*H_2OH), 52.6 (COO*C*H_3), 83.3 and 94.1 (*C*≡*C*), 122.1, 122.3, 133.1, 133.4, 135.0 and 135.4 (Ar-*C*), 165.2 (*C*=O); IR (neat, cm^{-1}) 3472, 3013, 1721, 1582, 1472, 1431, 1358, 1296, 1248, 1092, 1033, 965, 841, 785, 754, 542; HRMS (ESI-TOF): Calcd. for $C_{11}H_9NaBrO_3$ $[M^++Na]$: *m/z* 290.9629. Found: 290.9629 and 292.9602.

Compound 2h: Gummy liquid; yield: 1.20 g, 85%; ^{1}H NMR (400 MHz, $CDCl_3$) δ 1.58 (d, 3H, ^{3}J(H-H) = 4.0 Hz, $CH_2C$$H_3$), 2.05 (s, 1H, CHO*H*), 3.93 (s, 3H, COOCH_3), 4.79

(qrt, 1H, CH_3CHOH), 7.38-8.08 (m, 3H, Ar*H*); ^{13}C NMR (100 MHz, $CDCl_3$) δ 24.0 (s, CH_3CH), 52.5 (s, $COOCH_3$), 58.8 (s, CH_3CHOH), 81.7 and 97.6 (2 s, Ar*CC*CH), 122.0, 122.3, 133.2, 133.4, 134.8, 135.3 (Ar-*C*), 165.3 (s, *C*=O); IR (neat, cm^{-1}) 3445, 2982, 2951, 1728, 1717, 1584, 1551, 1478, 1435, 1395, 1291, 1242, 1096, 1034, 968, 864, 830, 787, 745; HRMS (ESI-TOF): Calcd. for $C_{12}H_{11}NaBrO_3$ [M^++Na]: *m/z* 304.9790. Found: 304.9793 and 306.9790.

Compound 2i: White solid; yield: 1.16 g, 78%; mp 116−118 °C; ^{1}H NMR (400 MHz, $CDCl_3$) δ 1.63 (s, 6H, $C(CH_3)_2$), 2.65 (s, 1H, CO*H*), 3.93 (s, 3H, $COOCH_3$), 7.36-8.07 (m, 3H, Ar*H*); ^{13}C NMR (100 MHz, $CDCl_3$) δ 31.0 and 31.1 (2 s, $C(CH_3)_2$), 52.5 (s, $COOCH_3$), 65.5 (s, *C*HOH), 79.9 and 100.4 (2 s, Ar*CC*C), 121.8, 122.4, 133.3, 134.8, 135.3, 138.1 (Ar-*C*), 165.4 (s, *C*=O); IR (KBr, cm^{-1}) 3436, 3074, 2975, 2953, 2931, 2225, 1726, 1649, 1474, 1430, 1282, 1244, 1173, 1090, 970, 745; HRMS (ESI-TOF): Calcd. for $C_{13}H_{13}NaBrO_3$ [M^++Na]: *m/z* 318.9946. Found: 318.9948 and 320.9921.

Compound 2j: Gummy liquid; yield: 1.35 g, 80%; ^{1}H NMR (400 MHz, $CDCl_3$) δ 1.28-2.03 (m, 10H, cyclohexyl-*H*), 3.05 (br s, 1H, C-O*H*), 3.90 (s, 3H, $COOCH_3$), 7.34-8.05 (m, 3H, Ar-*H*); ^{13}C NMR (100 MHz, $CDCl_3$) δ 23.3, 25.5 and 39.8 (cyclohexyl-*C*), 52.5 ($COOCH_3$), 69.2 (*C*-OH), 82.0 and 99.6 (*C*≡*C*), 121.7, 122.4, 133.3, 134.7, 135.4 (Ar-*C*), 165.4 (*C*=O); IR (neat, cm^{-1}) 3446, 3063, 2931, 2855, 2214, 1726, 1485, 1436, 1288, 1238, 1074, 970, 784; LC/MS *m/z* 338 $[M+1]^+$; Anal.Calcd. for $C_{16}H_{17}BrO_3$: C, 56.99; H, 5.08. Found: C, 56.85; H, 5.19.

(c) Experimental procedure for the synthesis of allenylphosphine oxides 3a-3c and 3m

To an oven-dried round-bottomed flask (50 mL) was added *N*-acetylated propargyl alcohol **1a** (0.52 g, 2.41 mmol), Et_3N (0.40 mL, 2.89 mmol) and tetrahydrofuran (10 mL) under N_2 atmosphere. To this was added chlorodiphenylphosphine (0.52 mL, 2.89 mmol) in tetrahydrofuran (5 mL) drop-wise at 0 ^{o}C during 15 min; the contents brought to rt and stirred for 6–10 h. After the formation of the allene (TLC), the reaction mixture was filtered and the solvent removed from the filtrate under reduced pressure. Purification by column chromatography (hexane/ethyl acetate 2:1) afforded the desired product **3a**.

Compound 3a: White solid; yield: 0.85 g, (87%); mp 120-122 ^{o}C. ^{1}H NMR (400 MHz, $CDCl_3$) δ 1.42 (d, *J* = 6.0 Hz, 6H, C*H*$_3$), 2.24 (s, 3H, COC*H*$_3$), 6.95 (t, 1H, Ar-*H*), 7.10 (d, *J* = 7.6 Hz, 1H, Ar-*H*), 7.17 (t, 1H, Ar-*H*), 7.39-7.73 (m, 10H, Ar-*H*), 7.92 (d, *J* = 8.0 Hz, 1H, Ar-*H*), 10.87 (s, 1H, N*H*). ^{13}C NMR (100 MHz, $CDCl_3$) δ 18.9 (d, *J* = 4.0 Hz *C*H$_3$), 24.2 (CO*C*H$_3$), 97.6 (d, *J* = 100.0 Hz, P*C*), 97.8 (d, *J* = 13.0 Hz PCC*C*), 124.0, 124.3, 125.7, 128.2, 128.4, 128.6, 130.3, 131.3, 131.4, 131.8, 132.0, 136.9 (Ar-*C*), 169.1 (*C*O), 211.7 (PC*C*C). ^{31}P NMR (162 MHz, $CDCl_3$) δ 35.19. IR (KBr, cm^{-1}) 3315, 3255, 3184, 2992, 2921, 1956, 1688, 1616, 1589, 1556, 1479, 1447, 1381, 1310, 1162, 1112, 767. HRMS (ESI) Calcd. for $C_{25}H_{24}NO_2P$ $(M + H)^+$: 402.1624. Found: 402.1625.

Compound 3b: Light yellow solid; yield: 0.59 g (78%; using 0.40 g (1.97 mmol) of propargyl alcohol **1b**); mp 114-116 ^{o}C. ^{1}H NMR (400 MHz, $CDCl_3$) δ 1.47 (dd, *J* = 7.6 Hz, *J* = 6.4 Hz, 3H, CHC*H*$_3$), 2.27 (s, 3H, COC*H*$_3$), 5.14 (qrt, 1H, C*H*CH$_3$), 6.98 (t, 1H, Ar-*H*), 7.11 (d, *J* = 7.6 Hz, 1H, Ar-*H*), 7.23 (t, 1H, Ar-*H*), 7.43-7.79 (m, 10H, Ar-

H), 7.95 (d, *J* = 8.0 Hz, 1H, Ar-*H*), 10.84 (s, 1H, N*H*). ^{13}C NMR (100 MHz, $CDCl_3$) δ 12.8 (d, *J* = 5.6 Hz CH*C*H_3), 24.4 (CO*C*H_3), 87.6 (d, *J* = 12.5 Hz, PCC*C*H), 99.4 (d, *J* = 99.5 Hz P*C*), 124.3, 124.5, 125.1, 128.2, 128.4, 128.6, 129.0, 129.9, 130.4, 131.0, 131.5, 131.6, 131.7, 131.7_9, 131.8_3, 132.3 (d, *J* = 5.6 Hz), 137.1 (Ar-*C*), 169.3 (*C*O), 213.0 (d, *J* = 7.9 Hz, PC*C*C). ^{31}P NMR (162 MHz, $CDCl_3$) δ 34.07. IR (KBr, cm^{-1}) 3441, 3238, 3052, 1945, 1688, 1611, 1584, 1540, 1479, 1436, 1370, 1304, 1271, 1162, 1118, 1101, 1068, 762. LC-MS: *m/z* 388 $[M+1]^+$. Anal. Calcd. for $C_{24}H_{22}NO_2P$: C, 74.41; H, 5.72; N, 3.62. Found: C, 74.32; H, 5.62; N, 3.75.

Compound 3c: Brown gummy liquid; yield: 1.07 g, (92%; using 0.70 g (2.51 mmol) of propargyl alcohol **1c**); ^{1}H NMR (400 MHz, $CDCl_3$) δ 1.85 (d, *J* = 6.0 Hz, 3H, C*H*$_3$), 2.28 (s, 3H, COC*H*$_3$), 7.01 (t, 1H, Ar-*H*), 7.12 (d, *J* = 7.6 Hz, 1H, Ar-*H*), 7.23-7.76 (m, 15H, Ar-*H*), 7.97 (d, *J* = 8.0 Hz, 1H, Ar-*H*), 10.80 (s, 1H, N*H*). ^{13}C NMR (100 MHz, $CDCl_3$) δ 16.2 (d, *J* = 5.0 Hz *C*H_3), 24.3 (CO*C*H_3), 101.5 (d, *J* = 98.0 Hz, P*C*), 103.4 (d, *J* = 13.0 Hz PCC*C*), 124.6 (d, *J* = 8.0 Hz), 125.1 (d, *J* = 4.0 Hz), 125.8, 127.9, 128.3, 128.4, 128.5, 128.8, 129.0, 129.9, 130.7, 131.0, 131.3, 131.4, 131.5, 132.6 (d, *J* = 3.0 Hz), 134.2 (d, *J* = 6.0 Hz), 137.0 (Ar-*C*), 169.2 (*C*O), 213.5 (d, *J* = 6.0 Hz, PC*C*C). ^{31}P NMR (162 MHz, $CDCl_3$) δ 34.45. IR (neat, cm^{-1}) 3457, 3249, 3052, 2920, 1934, 1687, 1578, 1539, 1435, 1369, 1298, 1161, 1123, 755. HRMS (ESI) Calcd. for $C_{30}H_{26}NO_2P$ $(M + H)^+$: 464.1780. Found: 464.1778.

Compound 3m: White solid; yield: 0.47 g (75%; using 0.37 g (1.40 mmol) of propargyl alcohol **1m**); mp 172-174 ^{o}C. ^{1}H NMR (400 MHz, $CDCl_3$) δ 1.42 (dd, *J* = 7.2 Hz, *J* = 4.4 Hz 3H, CHC*H*$_3$), 5.12 (m, 1H, C*H*CH$_3$), 7.03 (t, 1H, Ar-*H*), 7.11 (d, *J* = 7.6 Hz, 1H, Ar-*H*), 7.31 (t, 1H, Ar-*H*), 7.41-7.80 (m, 13H, Ar-*H*), 7.97 (d, *J* = 8.0 Hz, 1H, Ar-*H*), 8.33-8.35 (m, 2H, Ar-*H*), 11.36 (s, 1H, N*H*). ^{13}C NMR (100 MHz, $CDCl_3$) δ 12.8 (d, *J* = 5.4 Hz CH*C*H_3), 87.5 (d, *J* = 12.7 Hz, PCC*C*H), 98.8 (d, *J* = 100.9 Hz,

P*C*), 125.0, 126.0, 126.3, 127.4, 128.0, 128.4, 128.5, 128.8, 128.9, 129.1, 129.6, 130.3, 130.7, 131.4, 131.5_6, 131.6_1, 131.7, 131.8, 132.3 (d, *J* = 11.0 Hz), 134.7, 137.3 (Ar-*C*), 166.0 (*C*O), 212.0 (d, *J* = 8.2 Hz, PC*C*C). ^{31}P NMR (162 MHz, $CDCl_3$) δ 34.28. IR (KBr, cm^{-1}) 3227, 3058, 1940, 1666, 1605, 1578, 1534, 1436, 1310, 1173, 1118, 1101, 767 LC-MS: *m/z* 450 $[M+1]^+$. Anal.Calcd. for $C_{29}H_{24}NO_2P$: C, 77.49; H, 5.38; N, 3.12. Found: C, 77.58; H, 5.32; N, 3.21.

(d) Preparation of allenylphosphine oxides 4a–4j

Allenylphosphine oxides**4a–j**were synthesized according to literature procedures [12-13]. All of them are new. Spectroscopic and analytical data for these compounds are given below. All the compounds were purified by using ethyl acetate/hexane (1:1) mixture as eluent.

Compound 4a: This compound was prepared by using propargyl alcohol **2a** (0.95 g, 5.0 mmol) and chlorodiphenylphosphine (0.90 mL, 5.0 mmol). White solid; yield: 1.66 g, 85%; mp 64-66 ^{o}C; ^{1}H NMR (400 MHz, $CDCl_3$) δ 3.88 (s, 3H, COOCH_3), 4.80 (d, 2H, ^{4}J(P-H) = 10.4 Hz, PC*H*), 7.30-7.91 (m, 14H, Ar*H*); ^{13}C NMR: (100 MHz, $CDCl_3$) δ 52.3 (s, COO*C*H_3), 99.0 (d, ^{1}J(P-C) = 100.8 Hz, P*C*CCH_2), 127.6, 128.1, 128.2, 128.5, 128.6, 130.2, 130.6, 130.9, 131.2, 131.5, 131.7_5, 131.8, 131.9, 132.3, 132.6, 132.7 (Ar-*C* + PCC*C*), 168.0 (s, *C*OOMe) and 212.9 (d, ^{2}J(P-C) = 6.3 Hz, PC*C*CH_2); ^{31}P NMR (162 MHz, $CDCl_3$) δ 27.7; IR (KBr, cm^{-1}) 3040, 2955, 1931, 1713, 1593, 1485, 1437, 1269, 1186, 1088, 864, 797, 758, 702, 546; LC/MS *m/z* 375 $[M+1]^+$; Anal.Calcd. for $C_{23}H_{19}O_3P$: C, 73.79; H, 5.12. Found: C, 73.65; H, 5.18.

Compound 4b: This compound was prepared by using propargyl alcohol **2b** (1.02 g, 5.0 mmol) and chlorodiphenylphosphine (0.90 mL, 5.0 mmol). White solid; yield: 1.47 g, 79%; mp 78-80 ^{o}C; ^{1}H NMR (400 MHz, $CDCl_3$) δ 1.47 (t, ^{3}J(H-H) = 6.8 Hz,

=CHCH_3), 3.87 (s, 3H, COOCH_3), 5.14-5.28 (m, 1H, =C*H*Me), 7.24-7.87 (m, 14 H, Ar*H*); ^{13}C NMR (100 MHz, $CDCl_3$)δ 11.6 (d, ^4J(P-C) = 5.0 Hz, PCC*C*H_2), 52.1 (s, COO*C*H_3), 88.5(d, ^3J(P-C) = 13.0 Hz, PCC*C*Me), 99.1 (d, ^1J(P-C) = 103.0 Hz, P*C*CCMe), 127.0, 127.3, 127.9, 128.0, 129.9, 130.4, 131.0_0, 131.0_3, 131.2, 131.5, 131.6, 131.7, 131.8, 132.5, 132.9, 133.4, 133.5 (Ar-*C*), 168.1 (s, *C*OOMe) and 211.3 (d, ^2J(P-C) = 6.0 Hz, PC*C*CH_2); ^{31}P NMR (162 MHz, $CDCl_3$) δ 28.8; IR (KBr, cm^{-1}) 3059, 2949, 1946, 1717, 1593, 1487, 1437, 1373, 1262, 1188, 1119, 754, 706; LC/MS *m/z* 389 $[M+1]^+$; Anal.Calcd. for $C_{24}H_{21}O_3P$: C, 74.22; H, 5.45. Found: C, 74.12; H, 5.58.

Compound 4c: This compound was prepared by using propargyl alcohol **2c** (1.09 g, 5.0 mmol) and chlorodiphenylphosphine (0.90 mL, 5.0 mmol). White solid; yield: 1.70 g, 88%; mp 122-124 ^{o}C; ^{1}H NMR (400 MHz, $CDCl_3$) δ 1.46 and 1.48 (2 s, 6H, =C(CH_3)$_2$), 3.86 (s, 3H, COOCH_3), 7.24-7.91 (m, 14H, Ar*H*); ^{13}C NMR(100 MHz, $CDCl_3$) δ 18.3 (s, =C(*C*H_3)$_2$), 51.9 (s, COO*C*H_3), 97.6 (d, ^1J(P-C) = 103.8 Hz, P*C*CCMe$_2$), 98.9 (d, ^3J(P-C) = 12.6 Hz, PCC*C*Me$_2$), 127.0, 127.9, 128.0, 129.8, 130.5, 131.0, 131.3, 131.6, 131.7, 132.0, 133.1, 134.0 (d, ^2J(P-C) = 8.5 Hz, Ar-*C*), 168.2 (s, *C*OOMe) and 209.7 (s, PC*C*CMe$_2$); ^{31}P NMR (162 MHz, $CDCl_3$) δ 29.8; IR (KBr, cm^{-1}): 3052, 2986, 2951, 2915, 1946, 1725, 1591, 1487, 1439, 1372, 1291, 1264, 1184, 1086, 924, 760, 721, 698, 554; LC/MS *m/z* 403 $[M+1]^+$; Anal.Calcd. for $C_{25}H_{23}O_3P$: C, 74.62; H, 5.76. Found: C, 74.53; H, 5.68.

Compound 4d: This compound was prepared by using propargyl alcohol **2d** (1.29 g, 5.0 mmol) and chlorodiphenylphosphine (0.90 mL, 5.0 mmol). White solid; yield: 2.00 g, 90%; mp 100-102 ^{o}C; ^{1}H NMR (400 MHz, $CDCl_3$)δ 0.97-1.98 (m, 10H, cyclohexyl-*H*), 3.88 (s, 3H, COOCH_3), 7.23-7.94 (m, 14H, Ar*H*); ^{13}C NMR (100 MHz, $CDCl_3$) δ

25.4 and 26.2 (2 s, cyclohexyl-*C*), 29.1 (d, ^4J(P-C) = 4.6 Hz, cyclohexyl-*C*), 52.1 (s, COO*C*H_3), 97.8 (d, ^1J(P-C) = 104.4 Hz, P*C*CC), 105.0 (d, ^3J(P-C) = 13.0 Hz, PCC*C*), 127.1, 128.1, 128.2, 130.0, 130.7, 131.2, 131.5, 131.9, 132.0, 132.3, 133.4, 134.6 (d, ^2J(P-C) = 8.3 Hz, Ar-*C*), 168.5 (s, *C*OOMe), 206.7 (d, ^2J(P-C) = 6.3 Hz, PC*C*C); ^{31}P NMR (162 MHz, $CDCl_3$)δ 29.5; IR (KBr, cm^{-1}) 2932, 2855, 1941, 1719, 1485, 1435, 1262, 1161, 1123, 1084, 795, 779, 756, 723, 702; LC/MS *m/z* 442 $[M]^+$; Anal.Calcd. for $C_{28}H_{27}O_3P$: C, 76.00; H, 6.15. Found: C, 76.12; H, 6.21.

Compound 4e: This compound was prepared by using propargyl alcohol **2e** (1.33 g, 5.0 mmol) and chlorodiphenylphosphine (0.90 mL, 5.0 mmol). White solid; yield: 1.91 g, 85%; mp 98-100 ^oC; ^{1}H NMR: (400 MHz, $CDCl_3$) δ 3.81 (s, 3H, COOC*H*$_3$), 6.26 (d, *J* = 10.8 Hz, 1H, =C*H*), 7.17-7.46 (m, 13H, Ar*H*), 7.65-7.96 (m, 6H, Ar*H*); ^{13}C NMR (100 MHz, $CDCl_3$) δ 52.3 (s, COO*C*H_3), 97.1 (d, ^3J(P-C) = 12.6 Hz, PCC*C*), 104.6 (d, ^1J(P-C) = 99.0 Hz, P*C*CC), 127.6, 127.8, 127.9, 128.0, 128.2, 128.3, 128.6, 130.3, 131.0, 131.6_7, 131.7_3, 131.9, 132.0, 132.1, 132.4, 133.0, 168.0 (s, *C*OOMe) and211.5 (s, PC*C*CMe$_2$); ^{31}P NMR (162 MHz, $CDCl_3$) δ 27.5; IR (KBr, cm^{-1}) 1929, 1715, 1595, 1490, 1436, 1271, 1184, 1118, 750, 696, 559; LC/MS *m/z* 451 $[M+1]^+$; Anal.Calcd. for $C_{29}H_{23}O_3P$: C, 77.32; H, 5.15. Found: C, 77.46; H, 5.21.

Compound 4f: This compound was prepared by using propargyl alcohol **2f** (1.16 g, 5.0 mmol) and chlorodiphenylphosphine (0.90 mL, 5.0 mmol). White solid; yield: 1.70 g, 82%; mp 92-94 ^oC; ^{1}H NMR (400 MHz, $CDCl_3$) δ 0.74 (t, *J* ~ 7.6 Hz, 3H, CH_2CH_3), 1.52 (d, *J* = 6.0 Hz, 3H, C*H*$_3$), 1.70-1.86 (m, 2H, C*H*$_2$CH$_3$), 3.87 (s, 3H, COOC*H*$_3$), 7.22-7.46 (m, 8H, Ar*H*), 7.68-7.73 (m, 2H, Ar-*H*), 7.85-7.93 (m, 4H, Ar-*H*); ^{13}C NMR (100 MHz, $CDCl_3$) δ 11.6, 16.2 (d, ^4J(P-C) = 6.0 Hz, *C*H$_3$), 26.0 (d, ^4J(P-C) = 5.0 Hz, *C*H$_2$), 51.9 (s, COO*C*H$_3$), 99.1 (d, ^1J(P-C) = 104.0 Hz, P*C*CC), 104.5 (d, ^3J(P-C) =

13.0 Hz, PCC*C*), 127.1, 127.8, 127.9, 128.1, 129.9, 130.6, 131.1, 131.3, 131.7, 131.8, 132.2, 133.2, 134.3 (d, ^2J(P-C) = 8.0 Hz, Ar-*C*), 168.2 (s, *C*OOMe) and 209.0 (s, PC*C*C); ^{31}P NMR (162 MHz, $CDCl_3$) δ 29.2; IR (KBr, cm^{-1}) 3063, 2964, 1945, 1715, 1594, 1485, 1436, 1266, 1178, 1085, 921, 718, 696; LC/MS: *m/z* 417 [M+1]$^+$; Anal.Calcd. for $C_{26}H_{25}O_3P$: C, 74.99; H, 6.05. Found: C, 74.85; H, 6.12.

Compound 4g: This compound was prepared by using propargyl alcohol **2g** (0.67 g, 2.5mmol) and chlorodiphenylphosphine (0.45 mL, 2.5 mmol). White solid; yield: 0.94 g, 86%; mp 74-76 ^{o}C; ^{1}H NMR (400 MHz, $CDCl_3$) δ 3.89 (s, 3H, COOCH_3), 4.80 (d, 2H, ^4J(P-H) = 10.4 Hz, PCCCH_2), 7.43-7.89 (m, 13H, Ar*H*); ^{13}C NMR (100 MHz, $CDCl_3$) δ 52.6 (s, COO*C*H$_3$), 77.5 (d, ^3J(P-C) = 10.0 Hz, PCC*C*H$_2$), 98.4 (d, ^1J(P-C) = 100.5 Hz, P*C*CCH$_2$), 121.7, 128.2, 128.3, 131.1, 131.8, 131.9, 132.1, 132.6$_0$, 132.6$_4$, 133.2, 134.5, 166.7 (s, *C*OOMe) and 212.9 (d, ^2J(P-C) = 7.1 Hz, PC*C*CH$_2$); ^{31}P NMR (162 MHz, $CDCl_3$)δ 27.6; IR (KBr, cm^{-1}) 3052, 2969, 2946, 1946, 1912, 1723, 1480, 1435, 1289, 1242, 1192, 1092, 963, 723, 694, 552; LC/MS *m/z* 452 and 454 [M]$^+$; Anal.Calcd. for $C_{23}H_{18}BrO_3P$: C, 60.95; H, 4.00. Found: C, 60.85; H, 4.08.

Compound 4h: This compound was prepared by using propargyl alcohol **2h** (0.71 g, 2.5mmol) and chlorodiphenylphosphine (0.45 mL, 2.5 mmol). White solid; yield: 0.98 g, 84%; mp 90-92 ^{o}C; ^{1}H NMR (400 MHz, $CDCl_3$) δ 1.47 (dd→t, 3H, ^4J(P-H) ~ ^3J(H-H)~6.6 Hz, =CHCH_3), 3.88 (s, 3H, COOCH_3), 5.14-5.22 (m, 1H, =C*H*Me), 7.41-7.92 (m, 13H, Ar*H*); ^{13}C NMR (100 MHz, $CDCl_3$) δ 11.6 (d, ^4J(P-C) = 5.0 Hz, =CH*C*H$_3$), 52.4 (s, COO*C*H$_3$), 89.0 (d, ^3J(P-C) = 12.0 Hz, PCC*C*H$_2$), 98.5 (d, ^1J(P-C) = 103.0 Hz, P*C*CCH$_2$), 121.4, 128.1, 128.2, 130.3, 131.7, 131.8, 131.9, 132.1, 132.4, 132.6, 132.7, 132.8, 133.0, 134.3, 166.8 (s, *C*OOMe) and 211.4 (d, ^2J(P-C) = 5.0 Hz, PC*C*C); ^{31}P NMR (162 MHz, $CDCl_3$) δ 28.7; IR (KBr, cm^{-1}) 3052, 2953, 1951, 1726,

1583, 1485, 1436, 1288, 1244, 1184, 1096, 970; LC/MS *m/z* 467 and 469 $[M]^+$; Anal.Calcd. for $C_{24}H_{20}BrO_3P$: C, 61.69; H, 4.31. Found: C, 61.52; H, 4.38.

Compound 4i: This compound was prepared by using propargyl alcohol **2i** (0.74 g, 2.5mmol) and chlorodiphenylphosphine (0.45 mL, 5.0 mmol). White solid; yield: 1.10 g (91%); mp 132-134 ^oC; ^1H NMR (400 MHz, $CDCl_3$) δ 1.45 and 1.47 (2 s, 6H, $=C(CH_3)_2$), 3.87 (s, 3H, $COOCH_3$), 7.43-7.91 (m, 13H, Ar*H*); ^{13}C NMR (100 MHz, $CDCl_3$)δ 18.3 (d, ^4J(P-C) = 5.3 Hz, $=C(CH_3)_2$), 52.3 (s, $COOCH_3$), 96.9 (d, ^1J(P-C) = 103.3 Hz, P*C*CC), 99.4 (d, ^3J(P-C) = 12.9 Hz, PCC*C*), 121.1, 128.0, 128.2, 131.6, 131.7_0, 131.7_4, 132.1, 132.7, 132.8, 133.0_0, 133.0_3, 133.1, 133.2, 134.0 (Ar-*C*), 166.9 (s, *C*OOMe) and 209.9 (d, ^2J(P-C) = 5.4 Hz, $PC\mathit{C}CH_2$); ^{31}P NMR (162 MHz, $CDCl_3$) δ 29.8; IR (KBr, cm^{-1}) 3052, 2953, 2915, 2849, 1956, 1732, 1649, 1479, 1430, 1288, 1249, 1184, 1101, 838, 740; HRMS (ESI-TOF): Calcd. for $C_{25}H_{23}BrO_3P$ $[M^+ + H]$: *m/z* 481.0569 and 483.0569. Found: 481.0568 and 483.0545.

Compound 4j: This compound was prepared by using propargyl alcohol **2j** (0.84 g, 2.5 mmol) and chlorodiphenylphosphine (0.45 mL, 2.5 mmol). White solid; yield: 0.97 g, 74%; mp 102-104 ^oC; ^1H NMR: (400 MHz, $CDCl_3$) δ 0.94 (br s, 2H, cyclohexyl-*H*), 1.22-1.28 (m, 2H, cyclohexyl-*H*), 1.38-1.41 (m, 2H, cyclohexyl-*H*), 1.93 and 1.94 (2 br s, 4H, cyclohexyl-*H*), 3.87 (s, 3H, $COOCH_3$), 7.40-7.91 (m, 13H, Ar*H*); ^{13}C NMR (100 MHz, $CDCl_3$) δ 25.3 and 26.1 (2 s, cyclohexyl-*C*), 28.9 (d, ^4J(P-C) = 4.4 Hz, cyclohexyl-*C*), 52.4 (s, $COOCH_3$), 97.0 (d, ^1J(P-C) = 104.4 Hz, P*C*CC), 105.4 (d, ^3J(P-C) = 12.8 Hz, PCC*C*), 121.1, 128.2, 128.3, 131.6, 131.9, 132.0, 132.1, 132.2, 133.0, 133.1, 133.8, 134.1, 167.1 (s, *C*OOMe) and 206.8 (d, ^2J(P-C) = 6.1 Hz, PC*C*C); ^{31}P NMR (162 MHz, $CDCl_3$) δ 29.5; IR (KBr, cm^{-1}) 3057, 2931, 2849, 1951, 1732, 1479, 1436, 1293, 1249, 1189, 1096, 723; HRMS (ESI-TOF): Calcd. for

$C_{28}H_{27}BrO_3P$ [M^++H]: *m/z* 521.0882 and 523.0882. Found: 521.0882 and 523.0866.

(e) Synthesis of *N*-protected phosphinoyl indoles 5, 7 and 8

A mixture of *N*-acylated allenylphosphine oxide **3a** (0.48 g, 1.19 mmol), K_3PO_4 (0.13 g, 0.59 mmol) and tetrahydrofuran (10 mL) was stirred at 80 °C for 12 h. After completion of the reaction (TLC), the solvent was removed under reduced pressure. Purification the resulting product by column chromatography (hexane/ethyl acetate 1:1) afforded the desired product **5**. Compounds **7** and **8** were isolated using same method and the same molar quantity of *N*-benzoylated allenylphosphine oxide **3m**.

Compound 5: White solid; yield: 0.44 g, (92%); mp 218-220 °C; ^{1}H NMR (400 MHz, $CDCl_3$) δ 1.54 (br s, 3H, C*H*$_3$), 1.64 (d, *J* = 5.2 Hz, 3H, C*H*$_3$), 2.01 (br s, 3H, C*H*$_3$), 4.73 (d, *J* = 16.4 Hz, 1H, PC*H*), 6.30 (br s, 1H, Ar-*H*), 6.85 (t, 1H, Ar-*H*), 7.18-7.85 (m, 12H, Ar-*H*). ^{13}C NMR (100 MHz, $CDCl_3$) δ 20.5 and 21.5 (*C*H$_3$), 22.2 (*C*H$_3$), 48.3 (d, *J* = 62.0 Hz, P*C*H), 118.3, 124.0, 124.3, 126.3, 128.2 128.3, 128.5, 128.6, 131.4_5, 131.5_4, 132.2_5, 132.3_3, 132.5, 145.0 (d, *J* = 3.0 Hz), 169.6 (*C*O). ^{31}P NMR (162 MHz, $CDCl_3$) δ 26.69. IR (KBr, cm^{-1}) 3052, 2980, 2931, 1660, 1588, 1468, 1435, 1369, 1336, 1287, 1194, 1117, 871. HRMS (ESI) Calcd. for $C_{25}H_{24}NO_2P$ $(M + H)^+$: 402.1624. Found: 402.1627.

Compound 7: R_f = 0.55 (hexane/EtOAc 1:1); White solid; yield: 0.12 g, (60%; using 0.20 g (0.44 mmol) of allene **3m**); mp 184-186 °C; ^{1}H NMR (400 MHz, $CDCl_3$) δ 1.36 (dd, *J* = 12.0 Hz, 1.6 Hz, 3H, C*H*$_3$), 4.86 (d, *J* = 14.4 Hz, 1H, PC*H*), 5.37 (m, 1H, C*H*), 6.44 (d, *J* = 7.2 Hz, 1H, Ar-*H*), 6.80 (t, 1H, Ar-*H*), 7.11 (t, 1H, Ar-*H*), 7.28-7.87 (m, 16H, Ar-*H*). ^{13}C NMR (100 MHz, $CDCl_3$) δ 13.4 (d, *J* = 2.0 Hz, *C*H$_3$), 47.3 (d, *J* = 62.0 Hz, P*C*H), 116.0 (d, *J* = 9.0 Hz), 116.4, 123.5, 125.0 (d, *J* = 3.0 Hz), 125.3 (d, *J* = 6.0 Hz), 128.2, 128.5, 128.6, 128.6_5, 128.6_9, 128.8, 129.7, 130.9, 131.2, 131.8, 131.9,

132.1, 132.3, 132.4, 132.7, 135.9, 136.5 (d, J = 9.0 Hz), 144.7 (d, J = 4.0 Hz) (Ar-*C*), 168.5 (*C*O). ^{31}P NMR (162 MHz, $CDCl_3$) δ 27.31. IR (KBr, cm^{-1}) 3052, 2937, 1682, 1616, 1589, 1578, 1474, 1436, 1386, 1315, 1293, 1205, 1118, 932. HRMS (ESI) Calcd. for $C_{29}H_{24}NO_2P$ $(M + Na)^+$: 472.1443. Found: 472.1443. **Compound 8:** R_f = 0.45 (hexane/EtOAc 1:1); White solid; yield: 0.06 g, (31%; using 0.20 g (0.44 mmol) of allene **3m**); mp 168-170 ^oC; ^{1}H NMR (400 MHz, $CDCl_3$) δ 1.01 (t, 3H, CH_3), 3.18 (qrt, 2H, CH_2), 6.70 (d, J = 8.0 Hz, 1H, Ar-*H*), 6.89-6.92 (m, 2H, Ar-*H*), 6.99 (t, 1H, Ar-*H*), 7.47-7.81 (m, 15H, Ar-*H*). ^{13}C NMR (100 MHz, $CDCl_3$) δ 14.9 (CH_3), 20.2 (CH_2), 106.5 (d, J = 120.0 Hz), 113.4, 121.0, 122.6, 123.2, 128.7, 128.8, 129.2, 130.5, 131.9, 132.0, 132.1, 133.5, 134.0, 134.4, 134.5, 137.2 (d, J = 11.0 Hz), 153.4 (d, J = 18.0 Hz) (Ar-*C*), 169.9 (*C*O). ^{31}P NMR (162 MHz, $CDCl_3$) δ 23.45. IR (KBr, cm^{-1}) 3052, 2964, 2926, 2855, 1699, 1595, 1523, 1452, 1353, 1310, 1266, 1189, 1118, 1074, 975. HRMS (ESI) Calcd. for $C_{29}H_{24}NO_2P$ $(M + Na)^+$: 472.1443. Found: 472.1446.

(f) Synthesis of compounds 6 and 9 by deprotection of compounds 5, 7 and 8

In a 50 mL round-bottomed flask, *N*-acetylated phosphinoylindole **5** (0.25 g, 0.62 mmol) and NaOH (0.05 g, 1.24 mmol), were dissolved in an ethanol/water mixture (v/v 4:1, 20 mL), and the contents were heated with stirring at 80 °C for 8 h. After the completion of the reaction (TLC), the solvent was removed under reduced pressure. The crude product was purified by column chromatography (hexane/EtOAc; 1:1) affording **6** as a white solid. Simlarly compound **9** was obtained from the isomers 7 and **8**.

Compound 6. White solid; yield: 0.21 g, (93%); mp 242-244 ^{o}C; ^{1}H NMR (400 MHz, $CDCl_3$) δ 1.19 (d, *J* = 6.4 Hz, 6H, C*H*$_3$), 3.90 (m, 1H, C*H*), 6.54 (d, *J* = 8.4 Hz, 1H, Ar-*H*), 6.82 (t, 1H, Ar-*H*), 7.02 (t, 1H, Ar-*H*), 7.29 (d, *J* = 7.6 Hz, 1H, Ar-*H*), 7.40-7.77 (m, 10H, Ar-*H*), 10.21 (br s, 1H, N*H*). ^{13}C NMR (100 MHz, $CDCl_3$) δ 22.5 (*C*H$_3$), 26.6 (*C*H), 97.7 (d, *J* = 129.0 Hz, P*C*), 111.3, 120.1, 120.5, 121.6, 128.5, 128.6, 129.3 (d, *J* = 12.0 Hz), 131.7, 131.9, 132.0, 134.5, 135.5, 136.3 (d, *J* = 12.0 Hz), 156.0 (d, *J* = 17.0 Hz) (Ar-*C*). ^{31}P NMR (162 MHz, $CDCl_3$) δ 24.30. IR (KBr, cm^{-1}) 3161, 3068, 2970, 2865, 1490, 1457, 1435, 1293, 1156, 1117, 1101, 744. HRMS (ESI) Calcd. for $C_{23}H_{22}NOP$ (M + H)$^+$: 360.1518. Found: 360.1515.

Compound 9. White solid; yield: 0.11 g, (91%; using 0.15 g (0.34 mmol) mixture of *N*-benzoylated phosphinoyl indoles **7** and **8**); mp 214-218 ^{o}C; ^{1}H NMR (400 MHz, $CDCl_3$) δ 1.06 (t, 3H, CH$_2$C*H*$_3$), 2.88 (qrt, 2H, C*H*$_2$), 6.56 (d, *J* = 8.0 Hz, 1H, Ar-*H*), 6.81 (t, 1H, Ar-*H*), 7.01 (t, 1H, Ar-*H*), 7.26 (d, *J* = 8.4 Hz, 1H, Ar-*H*), 7.40-7.78 (m, 10H, Ar-*H*), 10.63 (br s, 1H, N*H*). ^{13}C NMR (100 MHz, $CDCl_3$) δ 14.3 (*C*H$_3$), 21.2 (*C*H$_2$), 98.4 (d, *J* = 128.0 Hz, P*C*), 111.3, 120.0, 120.5, 121.5, 128.5, 128.6, 129.6 (d, *J* = 13.0 Hz), 131.7, 131.8, 131.9, 134.4, 135.4, 136.3 (d, *J* = 12.0 Hz), 152.0 (d, *J* = 17.0 Hz) (Ar-*C*). ^{31}P NMR (162 MHz, $CDCl_3$) δ 23.95. IR (KBr, cm^{-1}) 3156, 3085, 3036, 2975, 2932, 1622, 1578, 1534, 1490, 1463, 1436, 1293, 1331, 1162, 1112, 1101, 1084, 756. HRMS (ESI) Calcd. for $C_{22}H_{20}NOP$ (M + H)$^+$: 346.1632. Found: 346.1634.

(g) One-pot synthesis of phosphinoyl indoles 6 and 9–19

To an oven-dried round-bottomed flask (50 mL) was added *N*-acetylated propargyl alcohol **1a** [7] (0.33 g, 1.52 mmol), Et_3N (0.25 mL, 1.82 mmol) and tetrahydrofuran (10 mL) under N_2 atmosphere. To this was added chlorodiphenylphosphine (0.33

mL, 1.82 mmol) dissolved in 5 mL of tetrahydrofuran drop-wise at 0 °C during 15 min; the contents brought to rt and stirred for 6–10 h. After formation of the allene (TLC), NaOH (0.08 g, 2.04 mmol) and an ethanol/water mixture (v/v 2:1, 15 mL) were added and then the mixture was stirred further at 80 °C for 8–10 h. After completion of the reaction (TLC), the solvent was removed under reduced pressure. The residue was dissolved in ethyl acetate (20 mL), washed with water (2 × 10 mL) and then with brine (10 mL). The organic part was dried over anhyd. Na_2SO_4 and the solvent was removed under reduced pressure. Purification by column chromatography (hexane/ethyl acetate 1:1) afforded the desired product **6** as a white solid; yield: 0.44 g, (80%). Analytical data are given above. Similarly compounds **9**–**19** were prepared.

Compound 9. White solid; yield: 0.41 g, (78%; using 0.31 g (1.53 mmol) of propargyl alcohol **1b**). Analytical data are given above.

Compound 10. White solid; yield: 0.26 g, (87%; using 0.2 g (0.72 mmol) of propargyl alcohol **1c**); mp 238-240 ^{o}C; ^{1}H NMR (400 MHz, $CDCl_3$) δ 1.64 (d, *J* = 7.2 Hz, 3H, C*H*$_3$), 5.37 (qrt, 1H, C*H*), 6.58 (d, *J* = 8.4 Hz, 1H, Ar-*H*), 6.83 (t, 1H, Ar-*H*), 7.01 (t, 1H, Ar-*H*), 7.14-7.80 (m, 16H, Ar-*H*), 9.48 (s, 1H, N*H*). ^{13}C NMR (100 MHz, $CDCl_3$) δ 20.4 (*C*H$_3$), 36.5 (*C*H), 99.3 (d, *J* = 126.0 Hz, P*C*), 111.3, 120.5, 120.8, 122.0, 126.6, 127.7, 128.4, 128.5_5, 128.6_0, 129.3 (d, *J* = 12.0 Hz), 131.7, 131.8, 131.9, 132.0, 134.2 (d, *J* = 9.0 Hz), 135.3 (d, *J* = 9.0 Hz), 136.1 (d, *J* = 11.0 Hz), 143.0, 153.5 (d, *J* = 17.0 Hz) (Ar-*C*). ^{31}P NMR (162 MHz, $CDCl_3$) δ 24.25. IR (KBr, cm^{-1}) 3167, 3068, 2986, 2942, 1479, 1458, 1436, 1288, 1162, 1123, 1074, 745**.** HRMS (ESI) Calcd. for $C_{28}H_{24}NOP$ $(M + H)^+$: 422.1675. Found: 422.1679.

Compound 11. White solid; yield: 0.53 g, (82%; using 0.37 g (1.95 mmol) of propargyl alcohol **1d**); mp 272-274 °C; ^{1}H NMR (400 MHz, $CDCl_3$+CD_3OD) δ 2.25 (d, *J* = 1.2 Hz, 3H, C*H*$_3$), 6.52 (d, *J* = 8.0 Hz, 1H, Ar-*H*), 6.76 (t, 1H, Ar-*H*), 6.97 (t, 1H, Ar-*H*), 7.23-7.66 (m, 11H, Ar-*H*), 11.03 (br s, 1H, N*H*). ^{13}C NMR (100 MHz, $CDCl_3$+CD_3OD) δ 13.4 (*C*H$_3$), 97.8 (d, *J* = 131.0 Hz, P*C*), 111.0, 119.5, 120.4, 121.5, 128.5, 128.6, 129.5 (d, *J* = 13.0 Hz), 131.5, 131.6, 131.8, 133.2, 134.2, 136.0 (d, *J* = 12.0 Hz), 145.8 (d, *J* = 18.0 Hz) (Ar-*C*). ^{31}P NMR (162 MHz, $CDCl_3$+CD_3OD) δ 26.51. IR (KBr, cm^{-1}) 3156, 3052, 2920, 2838, 1534, 1484, 1440, 1347, 1287, 1150, 1123, 1073, 810. HRMS (ESI) Calcd. for $C_{21}H_{18}NOP$ $(M + H)^+$: 332.1205.

Compound 12. White solid; yield: 0.91 g, (81%; using 0.69 g (2.99 mmol) of propargyl alcohol **1e**); mp 258-260 °C; ^{1}H NMR (400 MHz, $CDCl_3$) δ 0.68 (t, 3H, CH$_2$C*H*$_3$), 1.21 (d, *J* = 7.2 Hz, 3H, C*H*$_3$), 1.55-1.66 (m, 2H, C*H*$_2$), 3.69-3.74 (m, 1H, C*H*), 6.60 (d, *J* = 8.0 Hz, 1H, Ar-*H*), 6.84 (t, 1H, Ar-*H*), 7.05 (t, 1H, Ar-*H*), 7.30-7.78 (m, 11H, Ar-*H*), 9.70 (s, 1H, N*H*). ^{13}C NMR (100 MHz, $CDCl_3$) δ 12.1 and 20.6 (2 s, *C*H$_3$), 29.9 (*C*H$_2$), 33.1 (*C*H), 99.1 (d, *J* = 129.0 Hz, P*C*), 111.2, 120.3, 120.5, 121.6, 128.4_0, 128.4_4, 128.5, 128.6, 129.3 (d, *J* = 12.0 Hz), 131.6, 131.9, 132.0, 134.6 (d, *J* = 15.0 Hz), 135.7 (d, *J* = 14.0 Hz), 136.4 (d, *J* = 11.0 Hz), 155.1 (d, *J* = 18.0 Hz) (Ar-*C*). ^{31}P NMR (162 MHz, $CDCl_3$) δ 23.95. IR (KBr, cm^{-1}) 3145, 3074, 2964, 2871, 1479, 1435, 1331, 1293, 1166, 1117, 750. HRMS (ESI) Calcd. for $C_{24}H_{24}NOP$ $(M + H)^+$: 374.1675. Found: 374.1678.

Compound 13. White solid; yield: 0.26 g (63%; using 0.27 g (1.02 mmol) of propargyl alcohol **1f**); mp 192-194 °C; ^{1}H NMR (400 MHz, $CDCl_3$) δ 4.43 (s, 2H, C*H*$_2$), 6.65 (d, *J* = 8.0 Hz, 1H, Ar-*H*), 6.86 (t, 1H, Ar-*H*), 7.05 (t, 1H, Ar-*H*), 7.14-7.77 (m, 16H, Ar-*H*), 9.16 (s, 1H, N*H*). ^{13}C NMR (100 MHz, $CDCl_3$) δ 33.5 (*C*H$_2$), 99.3 (d, *J* = 128.0 Hz, P*C*), 111.5, 120.1, 120.5, 121.7, 126.3, 128.3_6, 128.4_2, 128.6. 129.0,

129.4 (d, *J* = 13.0 Hz), 131.6, 131.8, 131.9, 134.1, 135.1, 136.5 (d, *J* = 12.0 Hz), 138.4, 148.5 (d, *J* = 17.0 Hz) (Ar-*C*). ^{31}P NMR (162 MHz, $CDCl_3$) δ 24.49. IR (KBr, cm^{-1}) 3145, 3074, 2926, 2778, 1518, 1485, 1430, 1299, 1151, 1096, 1063, 745. HRMS (ESI) Calcd. for $C_{27}H_{22}NOP$ $(M + H)^+$: 408.1518. Found: 408.1519.

Compound 14. White solid; yield: 0.54 g, (69%; using 0.50 g (1.94 mmol) of propargyl alcohol **1g**); mp 268-270 ^{o}C; ^{1}H NMR (400 MHz, $CDCl_3$+CD3OD) δ 0.90-1.64 (m, 10H, CH_2), 2.76 (m, 1H, C*H*), 6.68 (d, *J* = 8.0 Hz, 1H, Ar-*H*), 6.77 (t, 1H, Ar-*H*), 6.98 (t, 1H, Ar-*H*), 7.30 (d, *J* = 8.0 Hz, 1H, Ar-*H*), 7.35-7.67 (m, 10H, Ar-*H*), 10.76 (br s, 1H, N*H*). ^{13}C NMR (100 MHz, $CDCl_3$+CD3OD) δ 25.6, 26.1 and 32.6 (*C*H_2), 36.4 (*C*H), 96.8 (d, *J* = 131.0 Hz, P*C*), 111.1, 120.1, 120.3, 121.5, 128.4, 128.5, 129.1 (d, *J* = 12.0 Hz), 131.6, 131.7, 132.3, 133.5, 134.6, 136.2 (d, *J* = 11.0 Hz), 154.3 (d, *J* = 19.0 Hz) (Ar-*C*). ^{31}P NMR (162 MHz, $CDCl_3$+CD3OD) δ 26.02. IR (KBr, cm^{-1}) 3167, 3117, 2980, 2931, 2849, 1484, 1430, 1287, 1156, 1101, 744. HRMS (ESI) Calcd. for $C_{26}H_{26}NOP$ $(M + H)^+$: 400.1831. Found: 400.1834.

Compound 15. White solid; yield: 0.35 g, (82%; using 0.25 g (1.22 mmol) of propargyl alcohol **1h**); mp 264-266 ^{o}C; ^{1}H NMR (400 MHz, $CDCl_3$+CD_3OD) δ 2.13 and 2.24 (2 s, 6H, CH_3), 6.36 (s, 1H, Ar-*H*), 6.85 (d, *J* = 8.0 Hz, 1H, Ar-*H*), 7.17 (d, *J* = 7.6 Hz, 1H, Ar-*H*), 7.42-7.72 (m, 10H, Ar-*H*), 10.96 (br s, 1H, N*H*). ^{13}C NMR (100 MHz, $CDCl_3$+CD_3OD) δ 13.5 and 21.3 (2 s, *C*H_3), 97.3 (d, *J* = 131.0 Hz, P*C*), 110.7, 119.4, 122.9, 128.4, 128.6, 129.5, 129.9 (d, *J* = 12.0 Hz), 131.6, 131.7, 133.3, 134.3 (d, *J* = 8.0 Hz), 145.7 (d, *J* = 19.0 Hz) (Ar-*C*). ^{31}P NMR (162 MHz, $CDCl_3$+CD_3OD) δ 26.55. IR (KBr, cm^{-1}) 3118, 3074, 3014, 2970, 2921, 2849, 1589, 1523, 1479, 1430, 1315, 1189, 1145, 1101, 1068, 805. HRMS (ESI) Calcd. for $C_{22}H_{20}NOP$ $(M + H)^+$: 346.1362. Found: 346.1359.

Compound 16. White solid; yield: 0.60 g, (85%; using 0.43 g (1.96 mmol) of propargyl alcohol **1i**); mp 252-254 ^{o}C; ^{1}H NMR (400 MHz, $CDCl_3$+CD_3OD) δ 1.03 (t, 3H, CH_3), 2.06 (s, 3H, CH_3), 2.70 (qrt, 2H, CH_2), 6.28 (s, 1H, Ar-*H*), 6.80 (d, *J* = 8.0 Hz, 1H, Ar-*H*), 7.16 (d, *J* = 8.0 Hz, 1H, Ar-*H*), 7.35-7.67 (m, 10H, Ar-*H*), 10.97 (br s, 1H, N*H*). ^{13}C NMR (100 MHz, $CDCl_3$+CD_3OD) δ 13.8 (*C*H_3), 20.9 (*C*H_2), 21.3 (*C*H_3), 96.4 (d, *J* = 131.0 Hz, P*C*), 110.8, 119.5, 122.9, 128.3, 128.5, 129.4, 129.7 (d, *J* = 12.0 Hz), 131.6, 131.7, 133.4, 134.5, 134.6 (d, *J* = 8.0 Hz), 151.6 (d, *J* = 18.0 Hz) (Ar-*C*). ^{31}P NMR (162 MHz, $CDCl_3$+CD_3OD) δ 26.80. IR (KBr, cm^{-1}) 3118, 3068, 3014, 2970, 2932, 1578, 1523, 1436, 1310, 1178, 1156, 805. HRMS (ESI) Calcd. for $C_{23}H_{22}NOP$ $(M + H)^+$: 360.1518. Found: 360.1522.

Compound 17. White solid; yield: 0.54 g, (79%; using 0.45 g (1.61 mmol) of propargyl alcohol **1j**); mp 230-232 ^{o}C; ^{1}H NMR (400 MHz, $CDCl_3$+CD_3OD) δ 2.13 (s, 3H, CH_3), 4.21 (s, 2H, CH_2), 6.39 (s, 1H, Ar-*H*), 6.86 (d, *J* = 8.4 Hz, 1H, Ar-*H*), 7.03-7.11 (m, 5H, Ar-*H*), 7.16 (d, *J* = 8.4 Hz, 1H, Ar-*H*), 7.38-7.70 (m, 10H, Ar-*H*), 10.38 (br s, 1H, N*H*). ^{13}C NMR (100 MHz, $CDCl_3$+CD_3OD) δ 21.3 (*C*H_3), 33.3 (*C*H_2), 98.3 (d, *J* = 130.0 Hz, P*C*), 110.9, 119.8, 123.4, 126.3, 128.3, 128.4, 128.5, 128.7, 129.6 (d, *J* = 12.0 Hz), 129.8, 131.7, 131.8, 133.3, 134.4, 134.6 (d, *J* = 12.0 Hz), 138.1, 147.9 (d, *J* = 17.0 Hz) (Ar-*C*). ^{31}P NMR (162 MHz, $CDCl_3$+CD_3OD) δ 26.63. IR (KBr, cm^{-1}) 3112, 3058, 2921, 2866, 1584, 1529, 1452, 1436, 1315, 1162, 1123, 1101, 800. HRMS (ESI) Calcd. for $C_{28}H_{24}NOP$ $(M + H)^+$: 422.1675. Found: 422.1677.

Compound 18. White solid; yield: 0.40 g, (68%; using 0.36 g (1.56 mmol) of propargyl alcohol **1k**); mp 248-250 ^{o}C; ^{1}H NMR (400 MHz, $CDCl_3$) δ 1.18 (d, *J* = 6.8 Hz, 6H, CH_3), 2.13 (s, 3H, CH_3), 3.79 (m, 1H, C*H*), 6.33 (s, 1H, Ar-*H*), 6.86 (d, *J* = 8.0 Hz, 1H, Ar-*H*), 7.19 (d, *J* = 8.4 Hz, 1H, Ar-*H*), 7.29-7.83 (m, 10H, Ar-*H*), 9.83 (br s, 1H, N*H*). ^{13}C NMR (100 MHz, $CDCl_3$) δ 21.6 and 22.5 (*C*H_3), 26.7 (*C*H), 97.3 (d, *J*

= 128.0 Hz, P*C*), 110.8, 120.1, 123.0, 128.4, 128.5, 129.7 (d, *J* = 17.0 Hz), 131.5, 131.9, 132.0, 134.4, 134.6 (d, *J* = 9.0 Hz), 135.7, 155.7 (d, *J* = 18.0 Hz) (Ar-*C*). ^{31}P NMR (162 MHz, $CDCl_3$) δ 24.78. IR (KBr, cm^{-1}) 3288, 3047, 2959, 2915, 1512, 1436, 1304, 1184, 1112, 701. HRMS (ESI) Calcd. for $C_{24}H_{24}NOP$ (M + H)$^+$: 374.1675. Found: 374.1678.

Compound 19. Light yellow solid; yield: 0.42 g, (74%; using 0.39 g (1.32 mmol) of propargyl alcohol **1l**); mp 212-214 ^{o}C; ^{1}H NMR (400 MHz, $CDCl_3$) δ 1.61 (d, *J* = 7.2 Hz, 3H, C*H*$_3$), 2.13 (s, 3H, C*H*$_3$), 5.23 (qrt, 1H, C*H*), 6.35 (s, 1H, Ar-*H*), 6.84 (d, *J* = 8.4 Hz, 1H, Ar-*H*), 7.10-7.79 (m, 16H, Ar-*H*), 9.48 (s, 1H, N*H*). ^{13}C NMR (100 MHz, $CDCl_3$) δ 20.3, 21.5 (2 s, *C*H$_3$), 36.7 (*C*H), 98.1 (d, *J* = 128.0 Hz, P*C*), 111.1 120.1, 123.2, 126.2, 127.6, 128.3, 128.4, 128.5, 129.6 (d, *J* = 13.0 Hz), 131.5, 131.6, 131.9_1, 131.9_4, 132.0, 134.2 (d, *J* = 27.0 Hz), 134.7 (d, *J* = 12.0 Hz), 135.3 (d, *J* = 27.0 Hz), 143.4, 153.2 (d, *J* = 18.0 Hz) (Ar-*C*). ^{31}P NMR (162 MHz, $CDCl_3$) δ 24.44. IR (KBr, cm^{-1}) 3128, 3047, 2970, 2921, 1584, 1436, 1321, 1189, 1151, 1118, 1074, 805. HRMS (ESI) Calcd. for $C_{29}H_{26}NOP$ (M + H)$^+$: 436.1831. Found: 436.1832.

(h) Synthesis of compounds 20–29

The respective alene (**4a–j**, 0.5 mmol) was treated with trifluoroacetic acid (2 mL) at rt for 6 h. The solvent was removed under vacuum and the crude product was purified by column chromatography using an ethyl acetate/hexane (2:3 v/v) mixture as the eluent to obtain the respective phosphinoyl isocoumarin **20–29**.

Compound 20 ,prepared from allenylphosphine oxide **4a** (0.18 g, 0.5 mmol): White solid; yield: 0.159 g, 88%; mp 134-136 ^{o}C; ^{1}H NMR (400 MHz, $CDCl_3$) δ 2.15 (s, 3H, C=CC*H*$_3$), 7.40-8.28 (m, 14H, Ar*H*). ^{13}C NMR (100 MHz, δ 21.2 (s, C=C*C*H$_3$), 105.8 (d, ^1J(P-C) = 105.0 Hz, P-*C*), 120.1 (d, ^2J(P-C) = 8.0 Hz, Ar-*C*), 126.8, 128.0, 129.0,

129.1, 129.5, 131.4, 131.5, 132.4, 132.7, 133.8, 134.3, 136.4 (d, ^2J(P-C) = 8.0 Hz, Ar-*C*), 161.1 (s, *C*OO), 163.2 (d, ^2J(P-C) = 18.0 Hz, P-C=*C*). ^{31}P NMR (162 MHz, $CDCl_3$) δ 27.5. IR (KBr, cm^{-1}) 3059, 2961, 1738, 1605, 1480, 1437, 1264, 1184, 1080, 1036, 801, 762, 687, 542, 523. LC/MS *m/z* 361 $[M+1]^+$. Anal. Calcd. for $C_{22}H_{17}O_3P$: C, 73.33; H, 4.76. Found: C, 73.48; H, 4.65.

Compound 21, prepared from allenylphosphine oxide **4b** (0.194 g, 0.5 mmol): White solid; yield: 0.153 g, 82%; mp 132-134 ^{o}C; ^{1}H NMR (400 MHz, $CDCl_3$) δ 1.03 (t, 3H, ^3J(H-H) = 7.4 Hz, CH_2CH_3), 2.60 (qrt, 2H, ^3J(H-H) = 7.3 Hz, CH_2CH_3), 7.39-7.83 (m, 14H, Ar*H*). ^{13}C NMR (100 MHz, $CDCl_3$) δ 12.4 (s, CH_2CH_3), 27.2 (s, CH_2CH_3), 105.1 (d, ^1J(P-C) = 105.2 Hz, P-*C*), 120.3 (d, ^2J(P-C) = 7.6 Hz, Ar-*C*), 127.1, 128.0, 129.0, 129.1, 129.6, 131.6, 131.7, 132.5, 132.9, 134.0, 134.3, 136.6 (d, ^2J(P-C) = 7.8 Hz, Ar-*C*), 161.4 (s, *C*OO), 168.0 (d, ^2J(P-C) = 18.6 Hz, P-C=*C*). ^{31}P NMR (162 MHz, $CDCl_3$) δ 28.4. IR (KBr, cm^{-1}) 3054, 2976, 2915, 1719, 1599, 1483, 1439, 1291, 1194, 1123, 1088, 1065, 777, 698, 540, 517. LC/MS *m/z* 375 $[M+1]^+$. Anal. Calcd. for $C_{23}H_{19}O_3P$: C, 73.79; H, 5.12. Found: C, 73.65; H, 5.18.

Compound 22, prepared from allenylphosphine oxide **4c** (0.201 g, 0.5 mmol): White solid; yield: 0.169 g, 87%; mp 200-202 ^{o}C; ^{1}H NMR (400 MHz, $CDCl_3$) δ 1.26 and 1.75 (2 d, 6H, ^5J(P-H) = 3.6 Hz and 5.2 Hz respectively, $=CH(CH_3)_2$), 4.78 (d, 1H, ^2J(P-H) = 18.0 Hz, PC*H*), 7.29-7.94 (m, 14H, Ar*H*). ^{13}C NMR (100 MHz, $CDCl_3$) δ 17.0 and 18.3 (2 s, $C(CH_3)_2$), 45.5 (d, ^1J(P-C) = 57.5 Hz, P*C*H), 118.6 (d, ^2J(P-C) = 9.8 Hz, C-C=*C*), 125.2, 127.7, 128.2, 128.4, 128.5, 128.9, 129.9, 130.0, 131.5, 131.6, 132.4, 132.6, 133.7, 134.3, 135.9 (d, ^2J(P-C) = 9.4 Hz, Ar-*C*), 162.1 (s, *C*OO). ^{31}P NMR (162 MHz, $CDCl_3$) δ 28.7. IR (KBr, cm^{-1}) 3058, 2928, 2855, 1730, 1678,

1595, 1439, 1231, 1183, 1161, 1117, 700, 534. LC/MS *m/z* 389 $[M+1]^+$. Anal.Calcd. for $C_{24}H_{21}O_3P$: C, 74.22; H, 5.45. Found: C, 74.11; H, 5.61.

Compound 23, prepared from allenylphosphine oxide **4d** (0.221 g, 0.5 mmol): White solid; yield: 0.178 g, 83%; mp 148-150 °C; ^{1}H NMR (400 MHz, $CDCl_3$) δ 0.57-0.67, 1.04-1.11 and 1.45-1.69 (m, 10H, cyclohexyl), 2.54 (m, 1H, cyclohexyl-C*H*), 7.39-8.30 (m, 14H, Ar*H*). ^{13}C NMR (100 MHz, $CDCl_3$) δ 25.2, 25.6, 29.9 (3 s, -$(CH_2)_5$-), 41.2 (s, CH_2CHCH_2), 104.8 (d, ^1J(P-C) = 106.6 Hz, P-*C*), 120.4 (d, ^2J(P-C) = 7.9 Hz, Ar-*C*), 127.5, 128.0, 128.3, 128.5, 129.1, 129.2, 129.5, 130.1, 131.5, 131.6, 132.4, 133.3, 133.7, 134.4, 136.7, 136.8, 161.3 (s, *C*OO), 169.0 (d, ^2J(P-C) = 18.9 Hz, P-C=*C*). ^{31}P NMR (162 MHz, $CDCl_3$) δ 28.1. IR (KBr, cm^{-1}) 2931, 2849, 1742, 1605, 1578, 1479, 1177, 1112, 1062, 777. LC/MS *m/z* 429 $[M+1]^+$. Anal.Calcd. for $C_{27}H_{25}O_3P$: C, 75.69; H, 5.88. Found: C, 75.48; H, 5.96.

Compound 24, prepared from allenylphosphine oxide **4e** (0.225 g, 0.5 mmol): White solid; yield: 0.191 g, 88%; mp 178-180 °C; ^{1}H NMR (400 MHz, $CDCl_3$) δ 4.61 (d, ^2J(PH) = 16.0 Hz, 1H, PC*H*), 5.46 (d, ^4J(PH) = 4.0 Hz, 1H, =C*H*Ph), 7.20-7.62 (m, 14H, Ar*H*), 7.70 (dd, *J* = 8.0 Hz, 2H, Ar*H*), 7.87 (dd, *J* = 8.0 Hz, 2H, Ar*H*), 7.98 (d, *J* = 8.0 Hz, 1H, Ar*H*). ^{13}C NMR (100 MHz, $CDCl_3$) δ 49.6 (d, ^1J(P-C) = 55.0 Hz, P-*C*), 114.7 (d, ^2J(P-C) = 9.0 Hz, Ar-*C*), 124.3, 127.5, 127.8, 128.1, 128.4, 128.6, 129.1, 130.1, 132.0 (d, ^2J(P-C) = 9.0 Hz, Ar-*C*), 132.3 (d, ^2J(P-C) ~ 9.0 Hz, Ar-*C*), 132.6, 133.1, 133.5, 133.9, 141.4 (d, ^2J(P-C) = 9.0 Hz, Ar-*C*), 160.6 (s, *C*OO). ^{31}P NMR (162 MHz, $CDCl_3$) δ 29.4. IR (KBr, cm^{-1}) 3029, 2956, 1737, 1666, 1441, 1304, 1244, 1195, 1068, 1030, 756, 701. HRMS (ESI) Calcd. for $C_{28}H_{22}O_3P$ $[M^+ +H]$: 437.1307. Found: 437.1305.

Compound 25, (*E*+*Z* 1:0.65 ratio), prepared from allenylphosphine oxide **4f** (0.208 g, 0.5 mmol). In the aliphatic region only major isomer peaks are given, but peaks due to the minor isomer are also present. White solid; yield: 0.172 g (86%; E+Z 1:0.65 ratio); mp 202-204 ^{o}C; ^{1}H NMR (400 MHz, $CDCl_3$) δ 0.91 (t, *J* = 7.6 Hz, 3H, CH_2CH_3), 1.28 (d, *J* = 3.2 Hz, 3H, CH_3), 2.11-2.31 (m, 2H, CH_2CH_3), 4.76 (d, ^{2}J(PH) = 17.6 Hz, 1H, PC*H*), 7.27-7.64 (m, 16H, Ar*H*), 7.90-7.99 (m, 4H, Ar*H*). ^{13}C NMR (100 MHz, $CDCl_3$) δ 11.9, 15.9, 23.8, 45.6 (d, ^{1}J(P-C) = 57.0 Hz, P-*C* for major isomer), 45.7 (d, ^{1}J(P-C) = 57.0 Hz, P-*C* for minor isomer), 123.8 (d, ^{2}J(P-C) = 10.0 Hz), 125.2, 128.0, 128.1, 128.3, 128.4, 128.5, 129.1, 130.1, 131.5, 131.6, 132.3, 132.4, 132.4_5, 132.5_3, 133.7, 133.8, 134.4 (d, *J* = 6.0 Hz), 134.7, 135.7 (d, ^{2}J(P-C) = 10.0 Hz, Ar-*C*), 135.9 (d, ^{2}J(P-C) = 10.0 Hz, Ar-*C*), 162.3 (s, *C*OO). ^{31}P NMR (162 MHz, $CDCl_3$) δ 28.0 (major) and 28.8 (minor). IR (KBr, cm^{-1}) 3061, 2912, 1737, 1671, 1436, 1315, 1232, 1184, 1156, 1123, 767, 701. HRMS (ESI) Calcd. for $C_{25}H_{24}O_3P$ [M^++H]: 403.1464. Found: 403.1467.

Compound 26, prepared from allenylphosphine oxide **4g** (0.227 g, 0.5 mmol): White solid; yield: 0.196 g, 89%; mp 186-188 ^{o}C; ^{1}H NMR (400 MHz, $CDCl_3$) δ 2.08 (s, 3H, C=CCH_3), 7.50-8.40 (m, 13H, Ar*H*). ^{13}C NMR (100 MHz, $CDCl_3$) δ 21.4 (s, C=CCH_3), 105.7 (d, ^{1}J(P-C) = 102.8 Hz, P-*C*=C), 121.8, 122.0, 128.6, 129.2, 129.3, 131.5, 131.6, 132.0, 132.4, 132.7, 133.4, 135.4 (d, ^{2}J(P-C) = 6.5 Hz, Ar-*C*), 159.9 (s, Ar*C*OO), 163.3 (d, ^{2}J(P-C) = 17.8 Hz, P-C=*C*). ^{31}P NMR (162 MHz, $CDCl_3$) δ 27.6. IR (KBr, cm^{-1}) 3058, 2924, 2855, 1746, 1599, 1474, 1437, 1316, 1265, 1231, 1186, 1119, 1078, 814, 725, 698. LC/MS *m/z* 439 $[M+1]^+$ and 441 $[M+3]^+$. Anal.Calcd. for $C_{22}H_{16}BrO_3P$: C, 60.16; H, 3.67. Found: C, 60.26; H, 3.61.

Compound 27, prepared from allenylphosphine oxide **4h** (0.237 g, 0.5 mmol): White solid; yield: 0.195 g, 86%; mp 192-194 °C; ^{1}H NMR (400 MHz, $CDCl_3$) δ 0.99 (t, 3H, ^{3}J(H-H) = 7.4 Hz, CH_2CH_3), 2.53 (qrt, 2H, ^{3}J(H-H) = 7.1 Hz, CH_2CH_3), 7.51-8.42 (m, 13H, Ar*H*). ^{13}C NMR (100 MHz, $CDCl_3$) δ 12.2 (s, CH_2CH_3), 27.3 (s, CH_2CH_3), 104.9 (d, ^{1}J(P-C) = 104.7 Hz, P-*C*=C), 122.0, 128.8, 129.1, 129.3, 131.5, 131.6, 132.0, 132.7, 137.4, 160.1 (s, *C*OO), 167.9 (d, ^{2}J(P-C) = 18.9 Hz, P-C=*C*). ^{31}P NMR (162 MHz, $CDCl_3$) δ 27.9. IR (KBr, cm^{-1}) 3052, 2920, 2849, 1742, 1599, 1473, 1430, 1183, 1090, 728. HRMS (ESI) Calcd. for $C_{23}H_{18}BrNaO_3P$ [M^++Na]: 475.0075 and 477.0075. Found: 475.0087 and 477.0070.

Compound 28, prepared from allenylphosphine oxide **4i** (0.241 g, 0.5 mmol): White solid; yield: 0.215 g, 92%; mp 240-242 °C; ^{1}H NMR (400 MHz, $CDCl_3$) δ 1.23 and 1.73 (2 s, 6H, $C(CH_3)_2$), 4.72 (d, 1H, ^{2}J(P-H) = 16.4 Hz, PC*H*), 7.09-8.08 (m, 13H, Ar*H*). ^{13}C NMR (100 MHz, $CDCl_3$) δ 17.1 and 18.4 (2 s, $C(CH_3)_2$), 45.2 (d, ^{1}J(P-C) = 57.1 Hz, P*C*H), 119.2 (d, ^{2}J(P-C) = 9.3 Hz, $C(CH_3)_2$), 122.3, 126.9, 127.7, 128.4, 128.6, 128.7, 129.7, 131.6, 131.7, 132.3, 132.4, 132.6, 132.8, 133.3, 135.5 (d, ^{2}J(P-C) = 9.9 Hz, Ar-*C*), 136.6 and 160.9 (s, *C*=O). ^{31}P NMR (162 MHz, $CDCl_3$) δ 28.3. IR (KBr, cm^{-1}) 3057, 2920, 2854, 1726, 1682, 1435, 1232, 1172, 706. HRMS (ESI) Calcd. for $C_{24}H_{20}BrNaO_3P$ [M^++Na]: 489.0231 and 491.0231. Found: 489.0270 and 491.0247.

Compound 29, prepared from allenylphosphine oxide **4j** (0.261 g, 0.5 mmol): White solid; yield: 0.216 g, 85%; mp 210-212 °C; ^{1}H NMR (400 MHz, $CDCl_3$) δ 0.85-2.32 (m, 10H, cyclohexyl-*H*), 4.76 (d, 1H, ^{2}J(P-H) = 12.0 Hz, PC*H*), 7.06-8.09 (m, 13H, Ar*H*). ^{13}C NMR (100 MHz, $CDCl_3$) δ 26.0, 26.2, 26.5 and 28.8 (4 s, cyclohexyl-*C*), 44.5 (d, ^{1}J(P-C) = 57.0 Hz, P-*C*), 122.2, 126.4, 127.1, 127.8, 128.5, 128.6, 128.8,

129.5, 131.6, 131.7, 132.3, 132.5, 132.7, 132.8, 133.1, 133.5 and 136.5 (Ar-*C*), 161.3 (s, *C*=O). ^{31}P NMR (162 MHz, $CDCl_3$) δ 28.8. IR (KBr, cm^{-1}) 3057, 2931, 2860, 1742, 1583, 1446, 1177, 701. HRMS (ESI) Calcd. for $C_{27}H_{25}BrO_3P$ [M^++H]: 507.0725 and 509.0725. Found: 507.0723 and 509.0670.

(i) Synthesis of compounds 30–35

The respective allene **4b–d** or **4h–j** (0.5 mmol) was treated with wet trifluoroacetic acid (2 mL; TFA:H_2O = 20:1) at 70 °C for 12 h. Solvent was removed under vacuum and the products were isolated by column chromatography using an ethyl acetate/hexane (2:3 v/v) mixture as the eluent to obtain the phosphinoylisocoumarins **21–23**, **27–29** along with the corresponding phosphorus-free isocoumarins **30–35**. Compounds **30–35** (higher R_f components) were separated from this mixture by column chromatography using ethyl acetate/hexane (1:20) as the eluent.

Compound 30, using allenylphosphine oxide **4b** (0.194 g, 0.5 mmol): Yield: 84% (**21** + **30**, isolated); 0.028 g (32%, **30**). The spectral data are in accordance with the literature reports [14].

Compound 31, using the allenylphosphine oxide **4c** (0.201 g, 0.5 mmol): Yield: 87% (**22** + **31**, isolated); 0.039 g (41%, **31**). The spectral data are in accordance with the literature reports [15].

Compound 32, using the allenylphosphine oxide **4d** (0.221 g, 0.5 mmol): Yield: 87% (**23** + **32**, isolated); 0.055 g (38%, **32**). The spectral data are in accordance with the literature reports [11].

Compound 33, using the allenylphosphine oxide **4h** (0.237 g, 0.5 mmol): White soild; yield: 88% (**27** + **33**, isolated); 0.048 g (38%, **33**); mp 74-76 ^{o}C; ^{1}H NMR (400 MHz, $CDCl_3$) δ 1.28 (t, 3H, ^{3}J(H-H) = 7.4 Hz, CH_2CH_3), 2.56 (qrt, 2H, ^{3}J(H-H) = 7.3 Hz, CH_2CH_3), 6.23 (s, 1H, C*H*=CEt), 7.24-8.38 (m, 3H, Ar*H*). ^{13}C NMR (100 MHz, $CDCl_3$) δ 11.2 (CH_2CH_3), 26.8 (CH_2CH_3), 101.4, 120.9, 121.6, 126.8, 132.1, 136.4, 137.9 and 160.2 (Ar-C), 161.8 (*C*OO). IR (KBr, cm^{-1}) 3068, 2920, 2849, 1737, 1660, 1474, 1156, 1041, 849. HRMS (ESI) Calcd. for $C_{11}H_9BrNaO_2$ [M$^+$+Na]: 274.9684 and 276.9684. Found: 274.9684 and 276.9670.

Compound 34, using allenylphosphine oxide **4i** (0.241 g, 0.5 mmol): White solid; yield: 90% (**28** + **34**, isolated); 0.047 g (35%, **34**); mp 78-80 ^{o}C; ^{1}H NMR (400 MHz, $CDCl_3$) δ 1.25-1.30 (m, 6H, $CH(CH_3)_2$), 2.76-2.80 (m, 1H, C*H*Me_2), 6.23 (s, 1H, C*H*=C), 7.25-8.39 (m, 3H, Ar-*H*). ^{13}C NMR (100 MHz, $CDCl_3$) δ 20.2 ($CH(CH_3)_2$), 32.5 (*C*HMe_2), 100.1, 120.9, 121.8, 127.0, 132.1, 136.5, 137.8 and 161.8 (Ar-*C*), 163.7 (*C*OO). IR (KBr, cm^{-1}) 2959, 2921, 2855, 1726, 1655, 1480, 1074, 844. HRMS (ESI) Calcd. for $C_{12}H_{11}BrNaO_2$ [M$^+$+Na]: 288.9840 and 290.9840. Found: 288.9868 and 290.9847.

Compound 35, using allenylphosphine oxide **4j** (0.261 g, 0.5 mmol): White solid; yield: 87% (**29** + **35**, isolated); 0.065 g (42%, **35**); mp 138-140 ^{o}C; ^{1}H NMR (400 MHz, $CDCl_3$) δ 1.25-2.46 (m, 11H, cyclohexyl-*H*), 6.20 (s, 1H, C*H*=C), 7.24-8.37 (m, 3H, Ar*H*). ^{13}C NMR (100 MHz, $CDCl_3$) δ 25.8, 26.0, 30.6 and 42.0 (cyclohexyl-*C*), 100.4, 120.8, 121.8, 127.0, 132.0, 136.5, 137.8 and 161.9 (Ar-*C*), 163.0 (*C*OO). IR (KBr, cm^{-1}) 2926, 2849, 1721, 1649, 1452, 1260, 855, 805. HRMS (ESI) Calcd. for $C_{15}H_{15}BrNaO_2$ [M$^+$+Na]: 329.0153 and 331.0153. Found: 329.0170 and 331.0151.

We have also isolated the phosphoryl indole (**B**) by using ($OCH_2CMe_2CH_2O$)PCl and propargyl alcohol **1c** by using the procedure shown in Scheme 4.

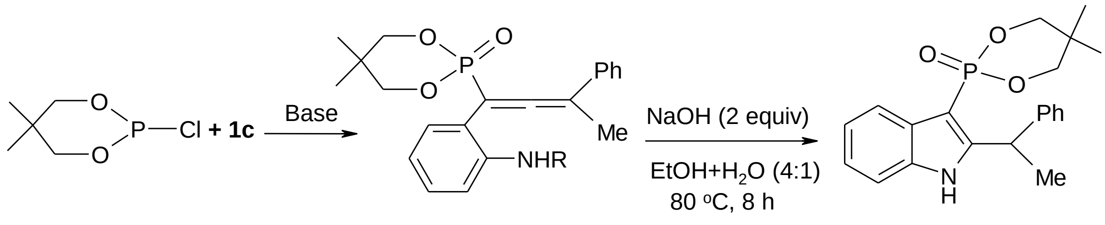

Compound A: White solid; yield: 0.37 g (72%; using 0.35 g (1.25 mmol) of propargyl alcohol **1c** and ($OCH_2CMe_2CH_2O$)PCl [16]); mp 80-82 °C. ^{1}H NMR (400 MHz, $CDCl_3$) δ 0.78 and 1.23 (2 s, 6H, C*H*$_3$), 2.16 (s, 3H, COC*H*$_3$), 2.25 (d, *J* = 6.8 Hz, 3H, C*H*$_3$), 3.86-4.08 (m, 4H, C*H*$_2$), 7.12 (t, 1H, Ar-*H*), 7.28-7.46 (m, 7H, Ar-*H*), 8.02 (d, *J* = 8.4 Hz, 1H, Ar-*H*), 9.55 (br s, 1H, N*H*). ^{13}C NMR (100 MHz, $CDCl_3$) δ 16.9 (d, *J* = 7.0 Hz, *C*H$_3$), 20.6, 21.8 and 24.3 (3 s, *C*H$_3$), 32.5 (d, *J* = 7.0 Hz, *C*(CH_3)$_2$), 77.3 and 77.4 (2 s, *C*H$_2$), 94.6 (d, *J* = 182.0 Hz, P*C*), 103.4 (d, *J* = 15.0 Hz PCC*C*), 123.9 (d, *J* = 7.0 Hz), 124.4, 124.8, 126.1, 128.4, 128.5, 128.6, 129.0, 129.4, 130.4 (d, *J* = 6.0 Hz), 132.1, 132.2, 132.9, 134.1 (d, *J* = 6.0 Hz), 136.6 (Ar-*C*), 169.2 (*C*O), 211.8 (PC*C*C). ^{31}P NMR (162 MHz, $CDCl_3$) δ 9.01. IR (KBr, cm^{-1}) 3277, 3058, 2964, 2932, 2921, 1945, 1688, 1578, 1523, 1436, 1370, 1249, 1052, 1003, 844. HRMS (ESI) Calcd. for $C_{23}H_{26}NO_4P$ $(M + Na)^+$: 434.1497. Found: 434.1494.

Compound B: White solid; yield: 0.19 g, (83%; using 0.25 g (0.62 mmol) of allenylphosphonate **A**); mp 184-186 °C; ^{1}H NMR (400 MHz, $CDCl_3$) δ 0.96 and 1.15 (2 s, 6H, C*H*$_3$), 1.53 (d, *J* = 7.2 Hz, 1H, C*H*$_3$), 3.55-4.23 (m, 4H, C*H*$_2$), 4.97 (qrt, 1H, C*H*), 7.14-7.84 (m, 9H, Ar-*H*), 10.08 (s, 1H, N*H*). ^{13}C NMR (100 MHz, $CDCl_3$) δ 20.7, 21.6 and 21.9 (3 s, *C*H$_3$), 32.5 (*C*(CH_3)$_2$), 37.6 (*C*H), 75.5 and 75.7 (*C*H$_2$), 94.0 (d, *J* = 215.0 Hz), 111.8, 120.1, 121.4, 122.4, 126.7, 127.5, 128.4 (d, *J* = 13.0 Hz), 136.2

(d, *J* = 14.0 Hz), 143.0, 152.3 (d, *J* = 26.0 Hz) (Ar-*C*). ^{31}P NMR (162 MHz, $CDCl_3$) δ 16.50. IR (KBr, cm^{-1}) 3162, 3129, 3090, 2964, 2871, 1578, 1496, 1458, 1436, 1299, 1249, 1222, 1118, 1058, 1014, 948. HRMS (ESI) Calcd. for $C_{21}H_{24}NO_3P$ $(M + H)^+$: 370.1573. Found: 370.1570.

References

[1]. Perrin, D. D.; Armarego, W. L. F.; Perrin, D. R. *Purification of Laboratory Chemicals*; Pergamon: Oxford, U.K., 1986.

[2]. Xiao, W-J.; Alper, H. *J. Org. Chem.* **1999**, *64*, 9646.

[3] Gimbert, C.; Vallribera, A. *Org. Lett.* 2009, *11*, 269-271. doi 10.1021/ol802346r

[4]. Evindar, G.; Batey, R. A. *J. Org. Chem.* **2006**, *71*, 1802.

[5]. Larock, R. C.; Harrison, L. W. *J. Am. Chem. Soc*. **1984**, *106*, 4218.

[6]. Zhou, N.; Wang, Li.; Thompson, D. W. Zhao, Y. *Org. Lett,* **2008**, *10*, 3001.

[7]. Arcadi, A.; Cacchi, S.; Fabrizi, G.; Marinelli, F.; Pace, P. *Eur. J. Org. Chem.* **1999**, 3305-3313. doi:10.1002/chin.200009044

[8]. Wang, M-K.; Zhou, Z-L.; Tang, R-Y.; Zhang, X-G.; Deng,. C-l. *Synlett.* **2013**, *24*, 737–740. doi: 10.1055/s-0032-1318347

[9]. Kleinbeck, F.; Toste. F. D. *J. Am. Chem. Soc.***2009**, *131*, 9178.

[10]. Sato, Y.; Ohashi, K.; Mori, M. *Tetrahedron Lett.***1999**, *40*, 5231.

[11]. Mikhailovskaya, T. F.; Vasilevsky, S. F. *Russ. Chem. Bull. Int. Ed.***2010**, *59*, 632.

[12]. Iorga, B.; Eymery, F.; Carmichael, D.; Savignac, P. *Eur. J. Org. Chem.* **2000**, 3103.

[13] Bhuvan Kumar, N. N.; Chakravarty, M.; Satish Kumar, N.; Sajna, K. V.; Kumara Swamy, K. C. *J. Chem. Sci*. **2009**, *121*, 23.

[14] Cherry, K.; Parrain, J. L.; Thibonnet, J.; Duchene, A.; Abarbri, M. *J. Org. Chem.* **2005**, *70*, 6669. doi: 10.1021/jo050638z.

[15] Hauser, F. M.; Baghdanov, V. M. *J. Org. Chem.* **1988**, *53*, 4676. doi: 10.1021/jo00255a006.

[16] Kumara Swamy, K. C.; Kumaraswamy, S.; Senthil Kumar, K.; Muthiah, C. *Tetrahedron Lett.* **2005**, *46*, 3347.

^{1}H NMR and ^{13}C NMR Spectra

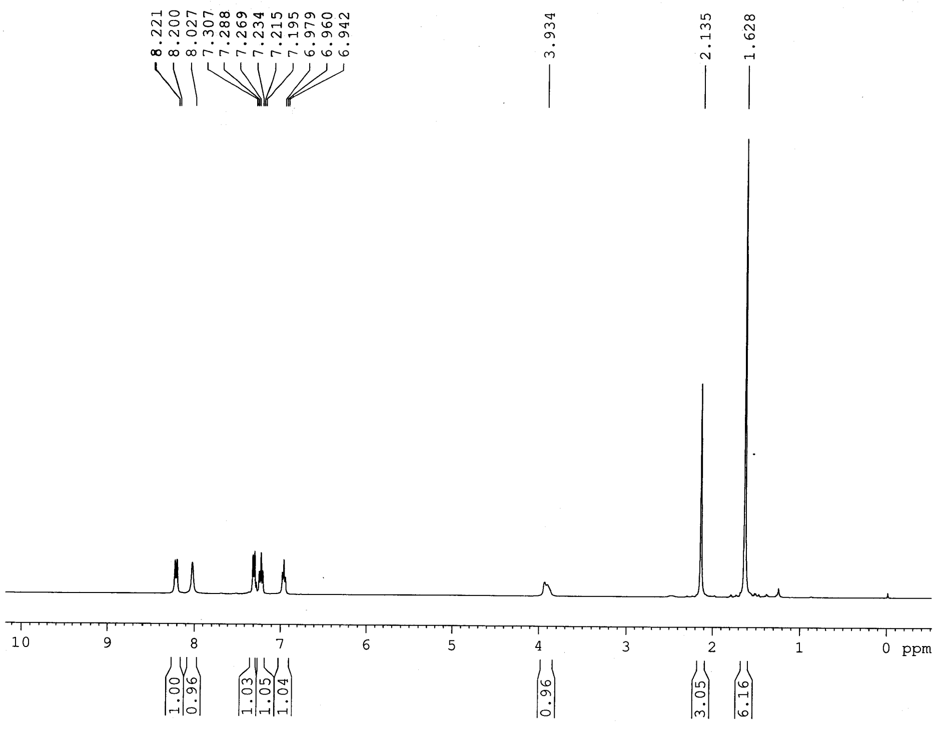

Figure S1: ^{1}H NMR spectrum of compound **1a**.

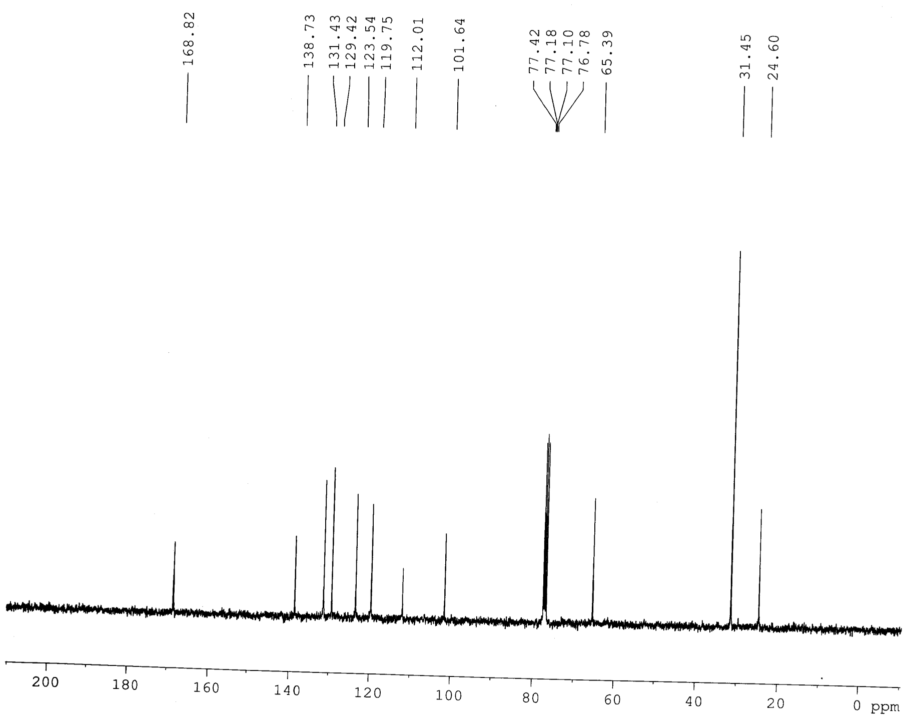

Figure S2: ^{13}C NMR spectrum of compound **1a**.

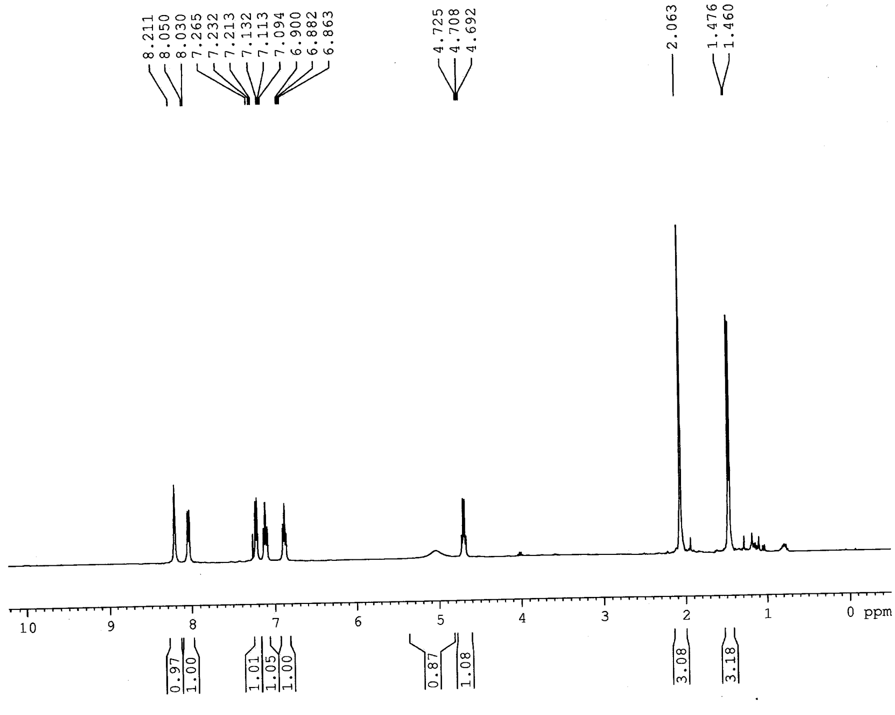

Figure S3: ^{1}H NMR spectrum of compound **1b**.

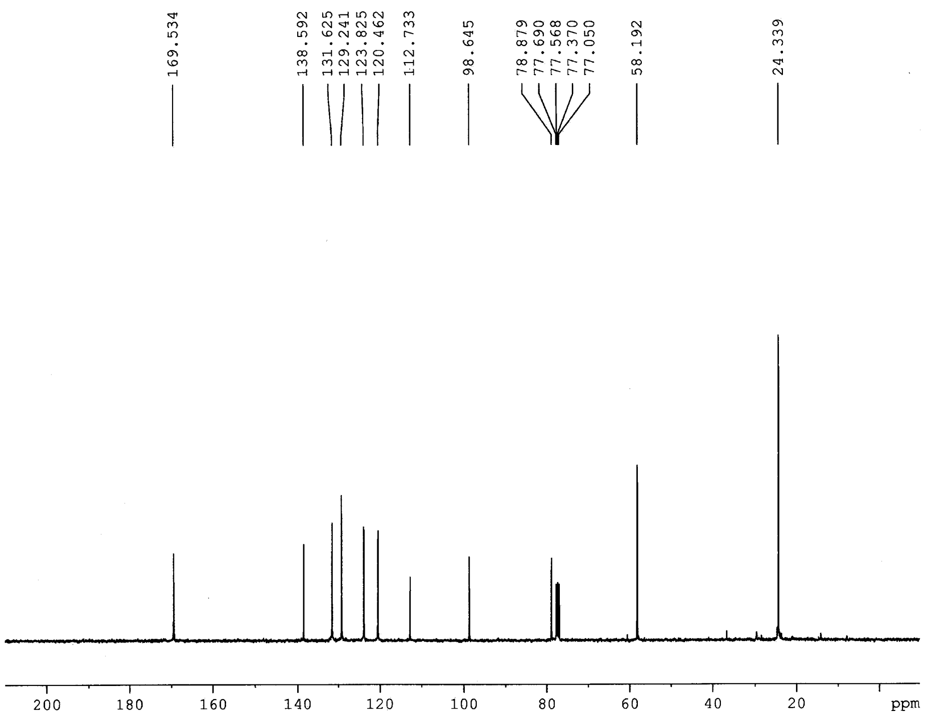

Figure S4: ^{13}C NMR spectrum of compound **1b**.

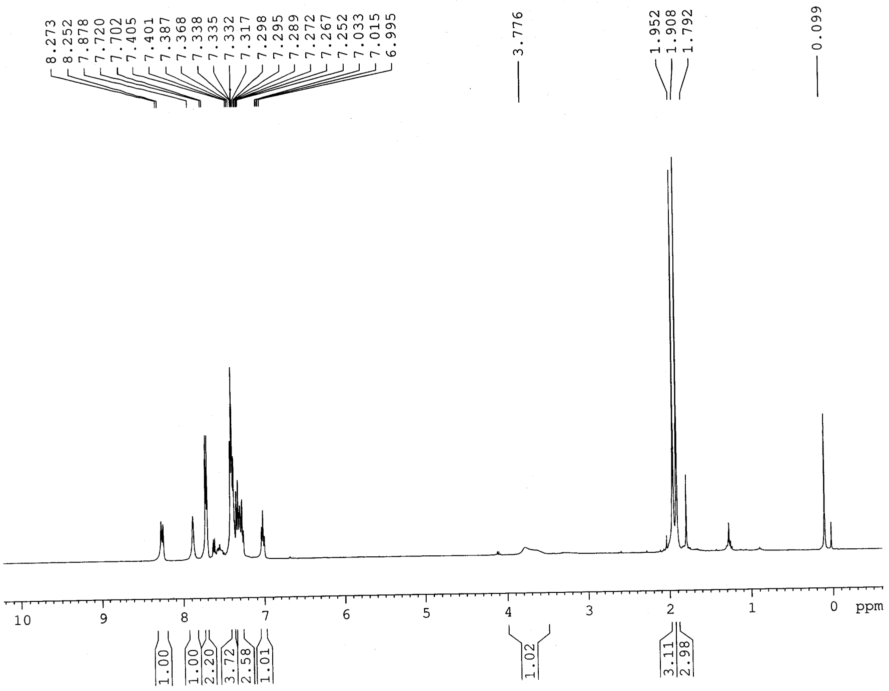

Figure S5: ^{1}H NMR spectrum of compound **1c**.

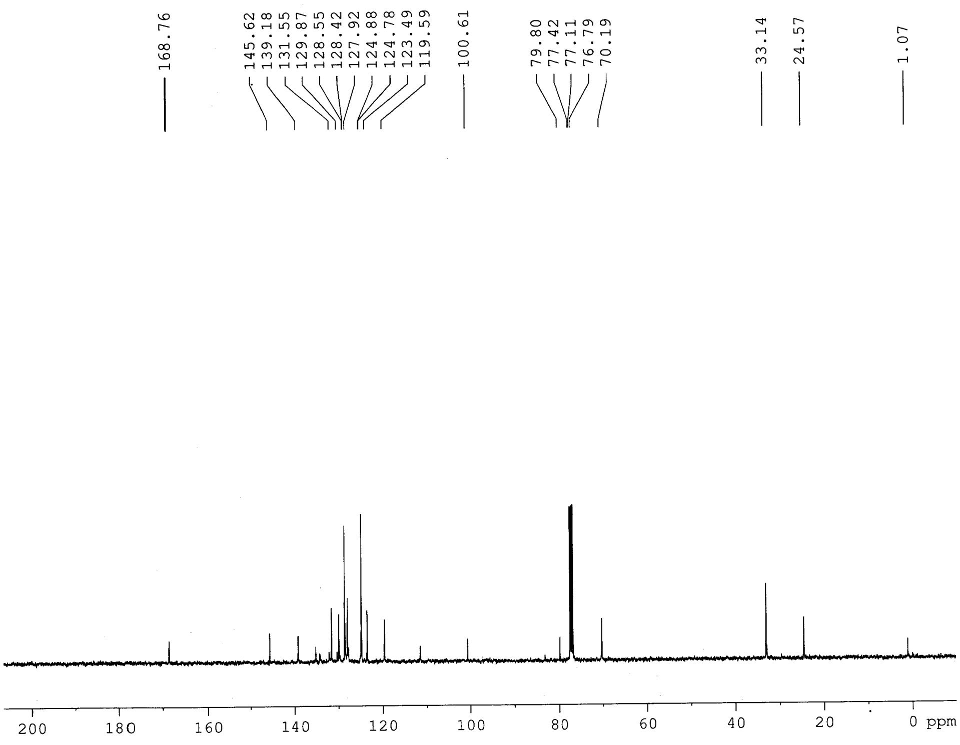

Figure S6: ^{13}C NMR spectrum of compound **1c**.

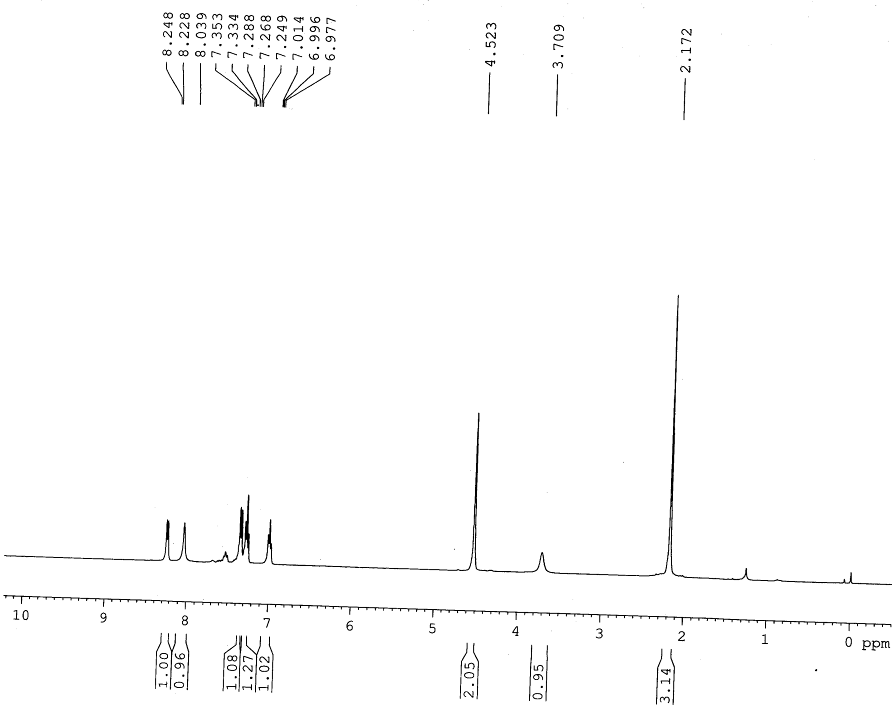

Figure S7: ^{1}H NMR spectrum of compound **1d**.

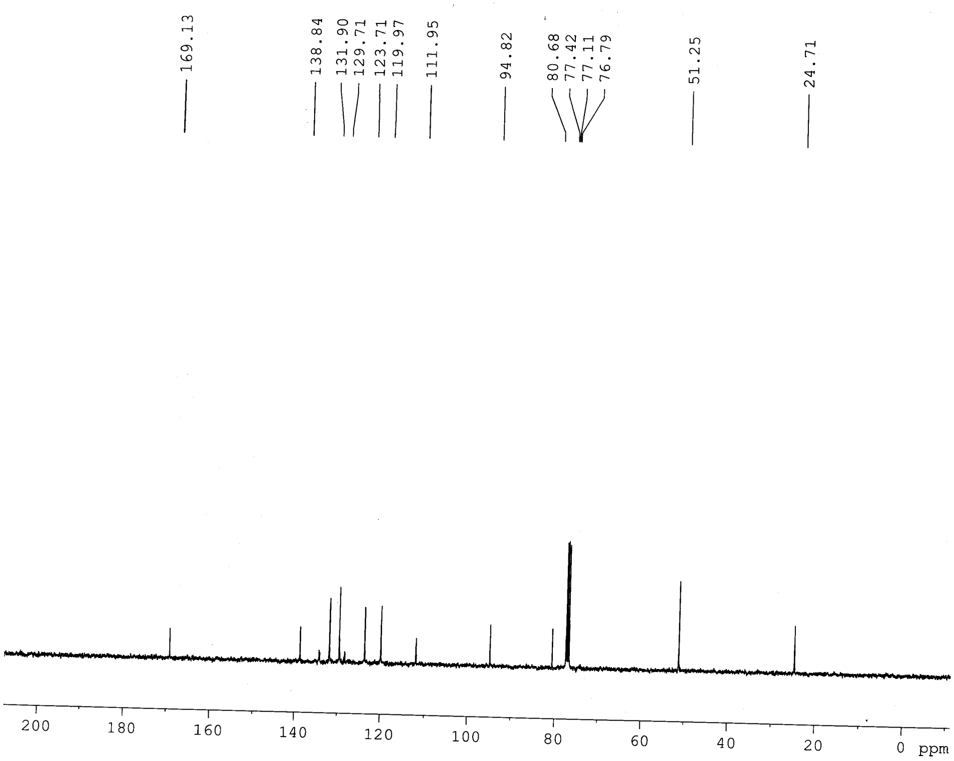

Figure S8: ^{13}C NMR spectrum of compound **1d**.

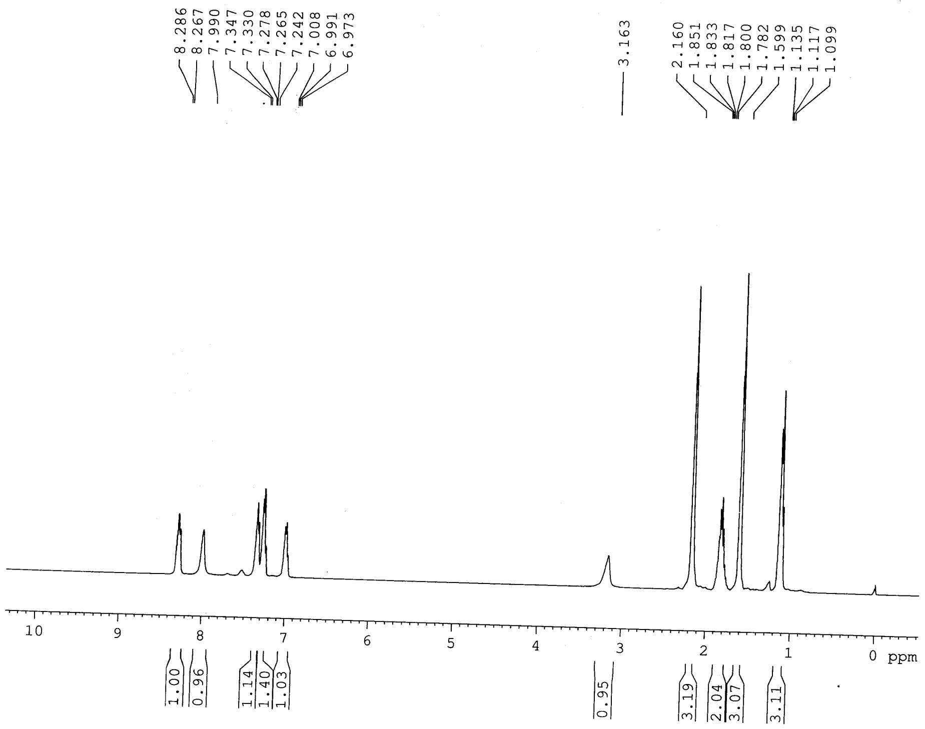

Figure S9: ^{1}H NMR spectrum of compound **1e**.

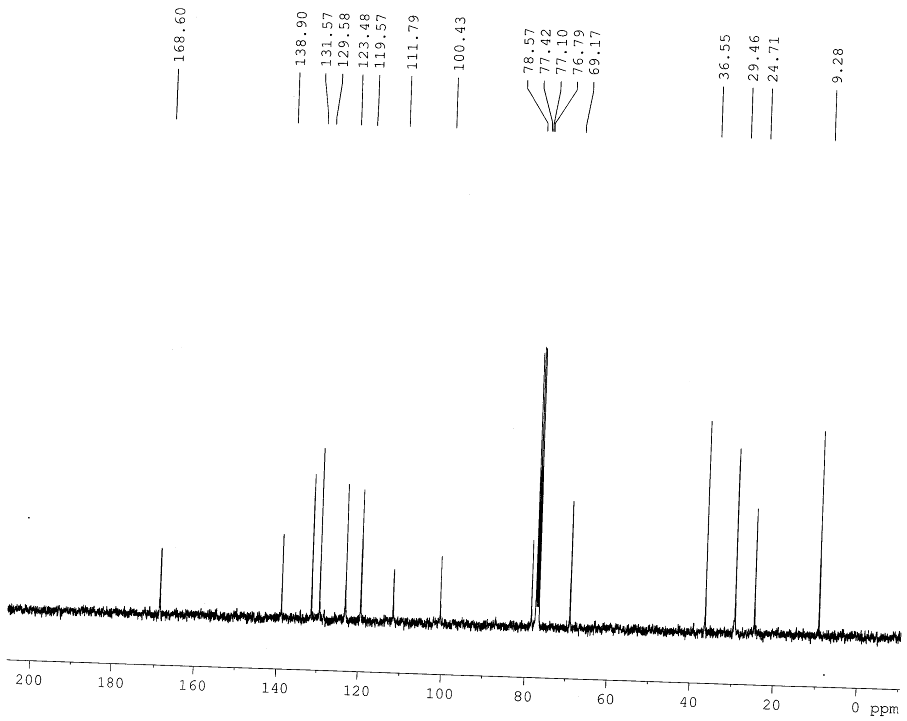

Figure S10: ^{13}C NMR spectrum of compound **1e**.

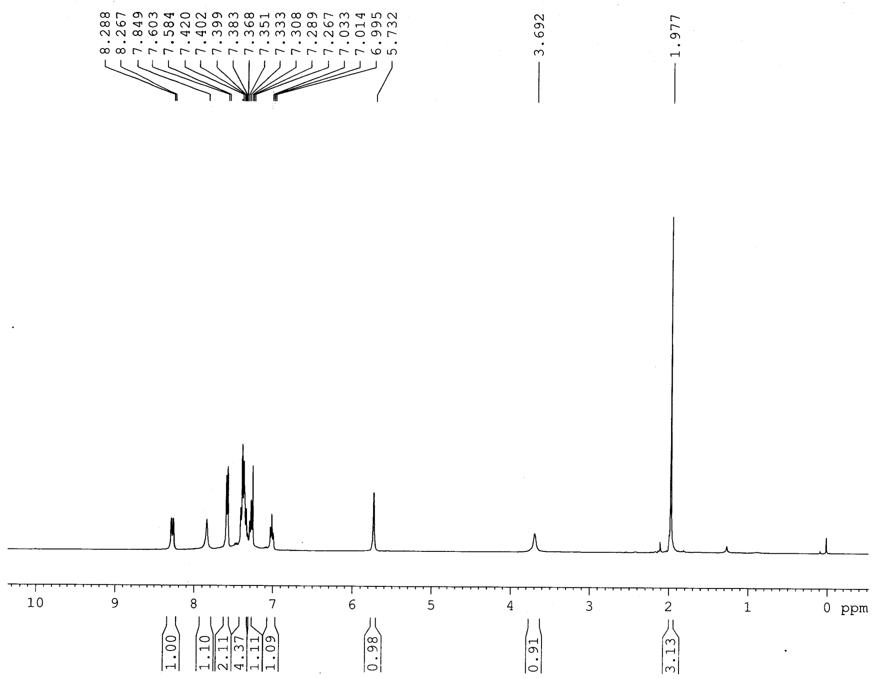

Figure S11: ^{1}H NMR spectrum of compound **1f**.

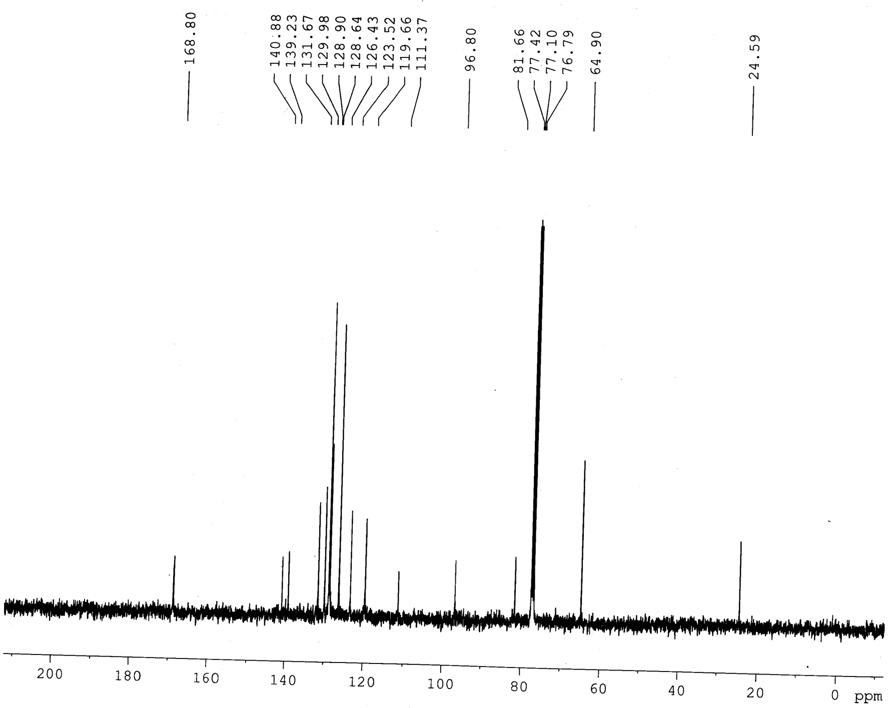

Figure S12: ^{13}C NMR spectrum of compound **1f**.

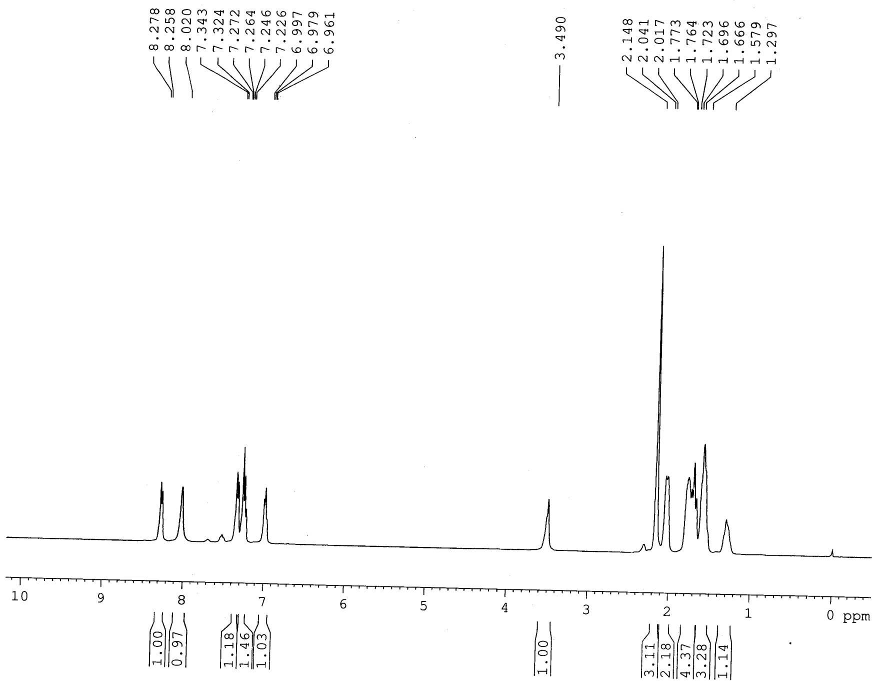

Figure S13: ^{1}H NMR spectrum of compound **1g**.

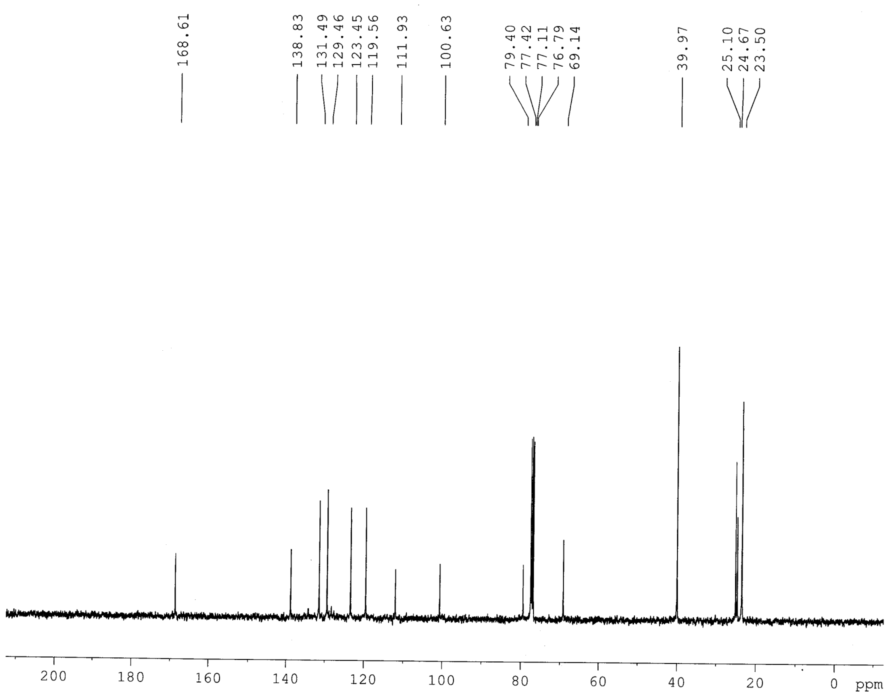

Figure S14: ^{13}C NMR spectrum of compound **1g**.

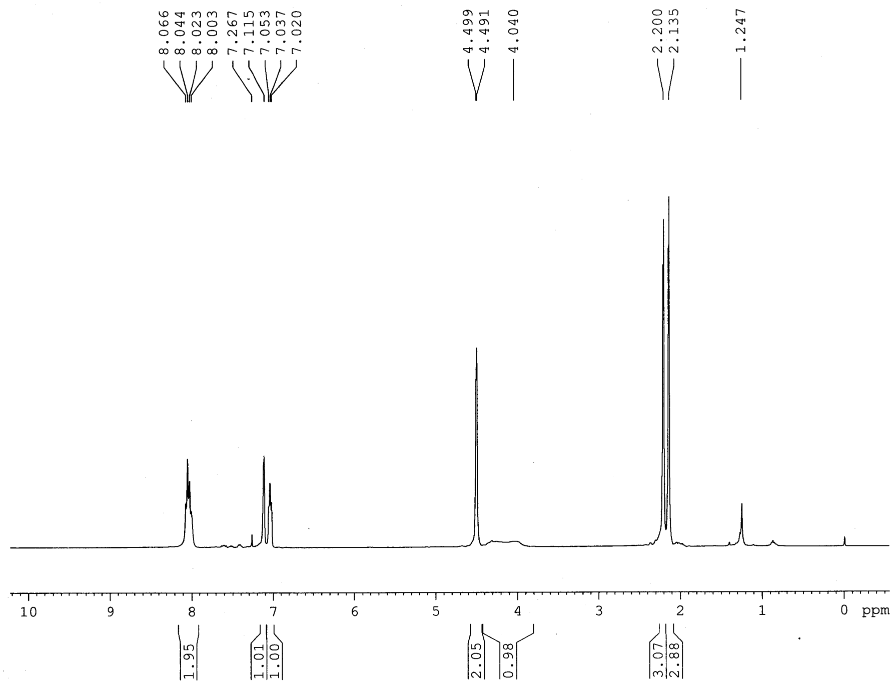

Figure S15: ^{1}H NMR spectrum of compound **1h**.

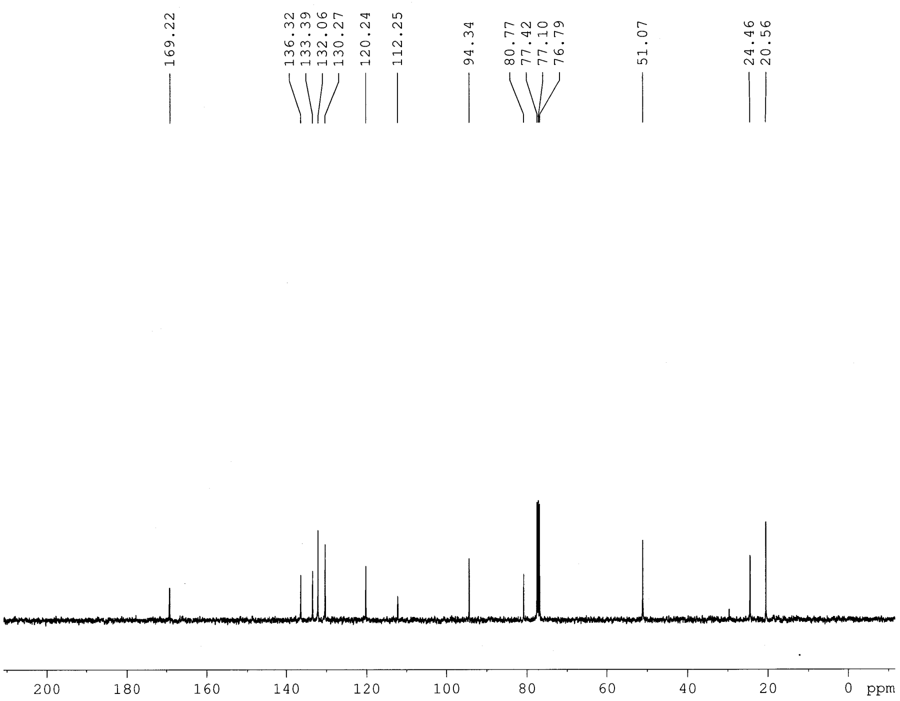

Figure S16: ^{13}C NMR spectrum of compound **1h**.

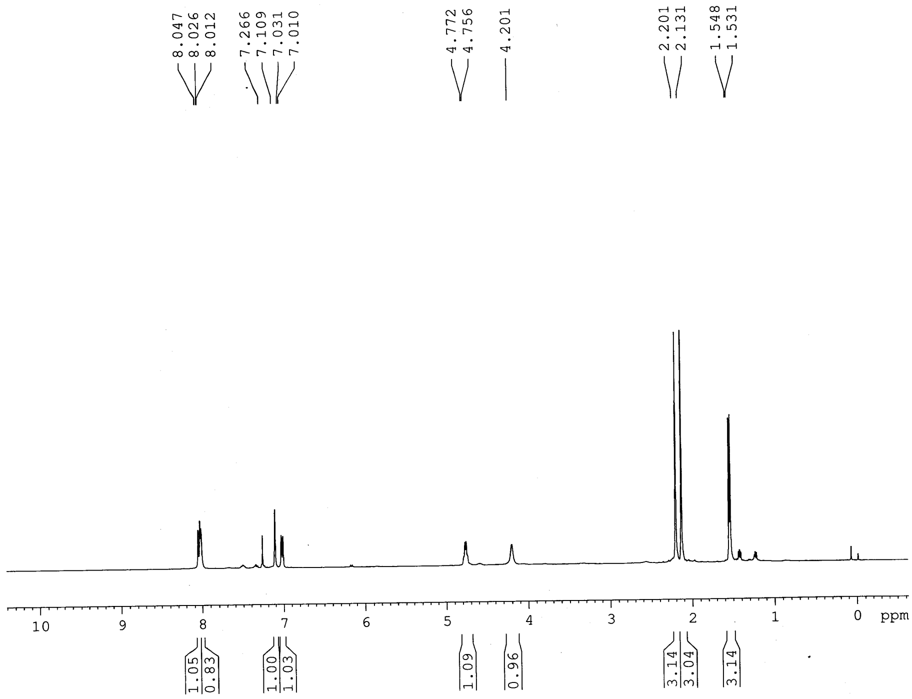

Figure S17: ^{1}H NMR spectrum of compound **1i**.

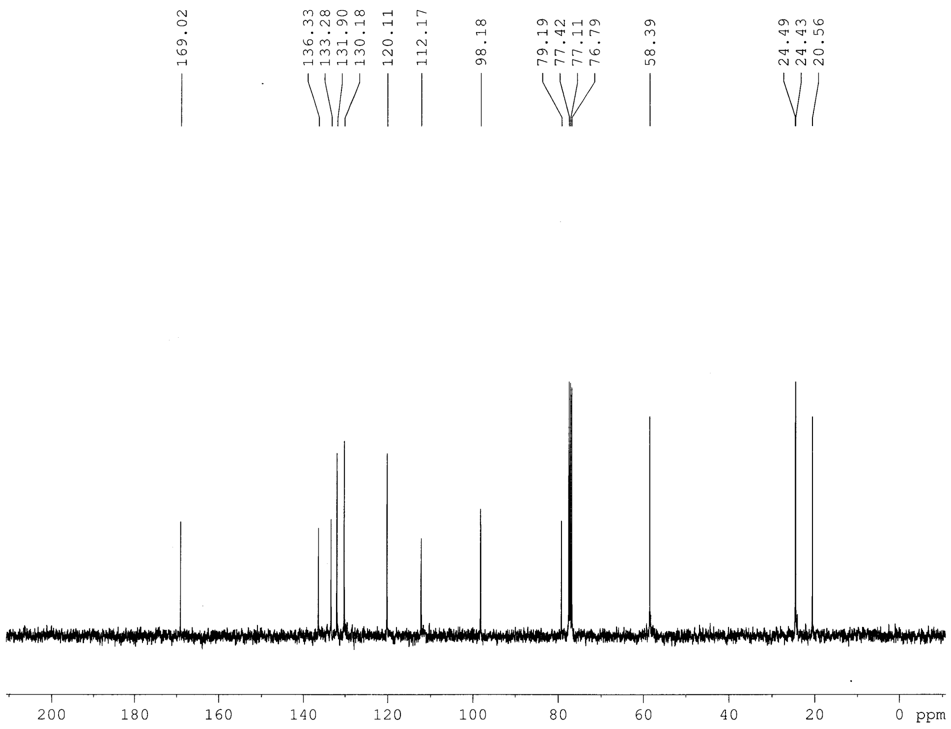

Figure S18: ^{13}C NMR spectrum of compound **1i**.

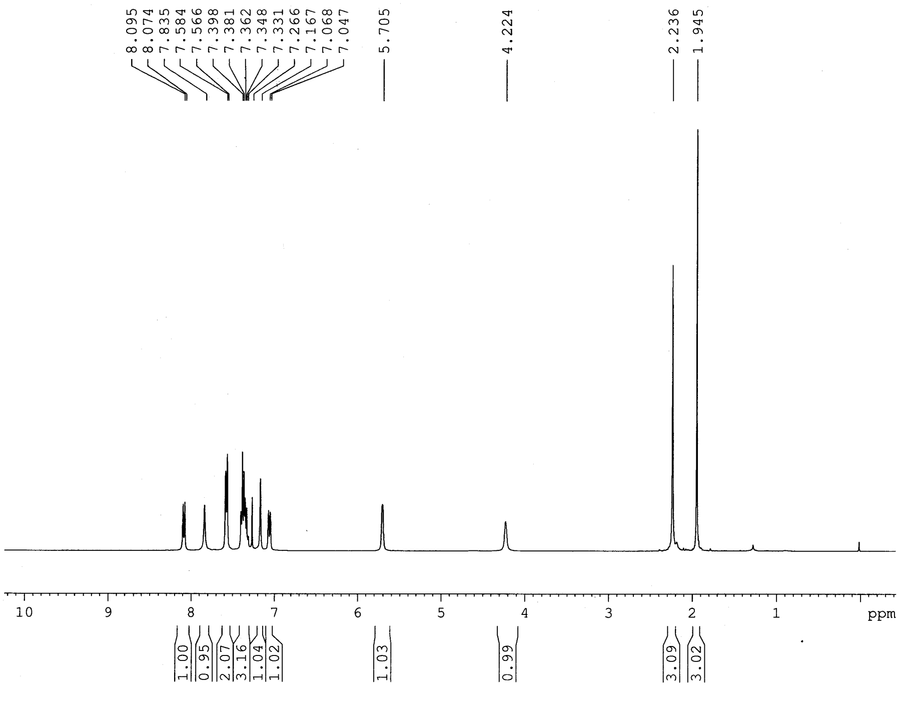

Figure S19: ^{1}H NMR spectrum of compound **1j**.

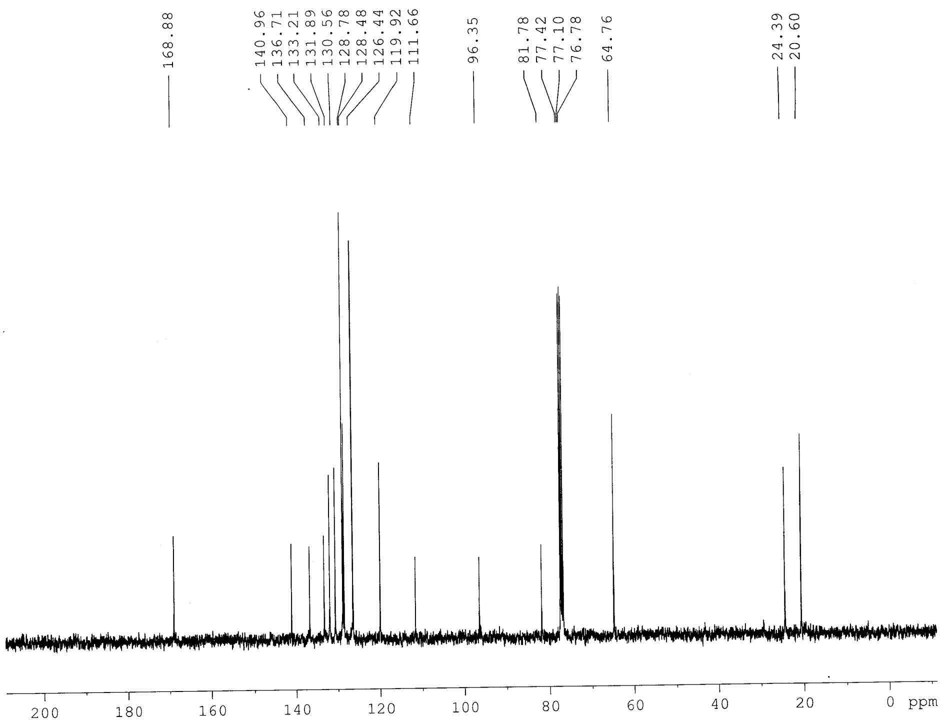

Figure S20: ^{13}C NMR spectrum of compound **1j**.

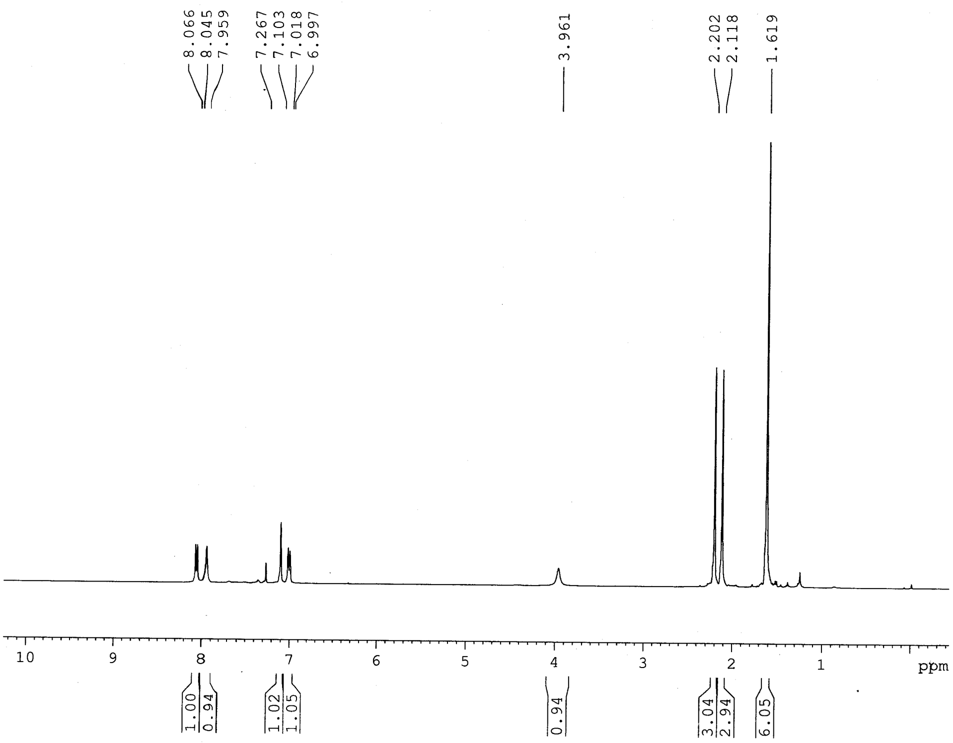

Figure S21: ^{1}H NMR spectrum of compound **1k**.

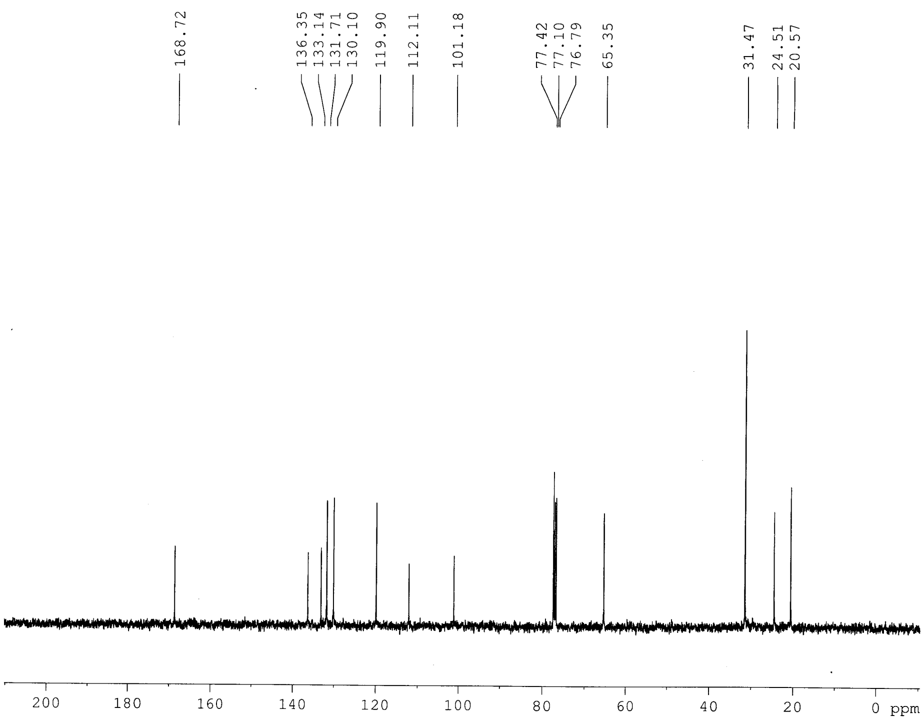

Figure S22: ^{13}C NMR spectrum of compound **1k**.

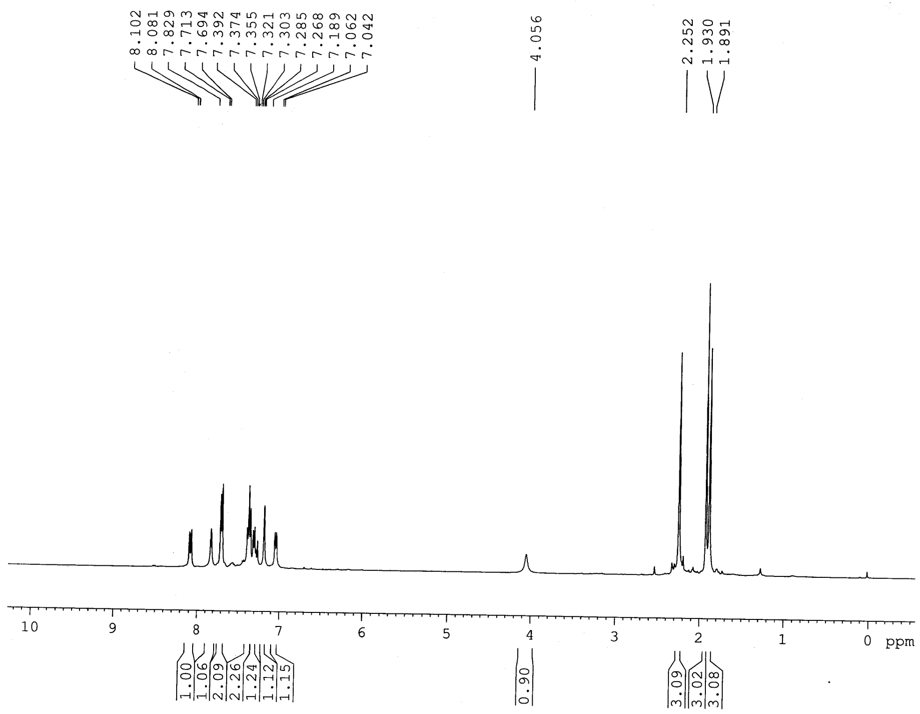

Figure S23: ^{1}H NMR spectrum of compound **1l**.

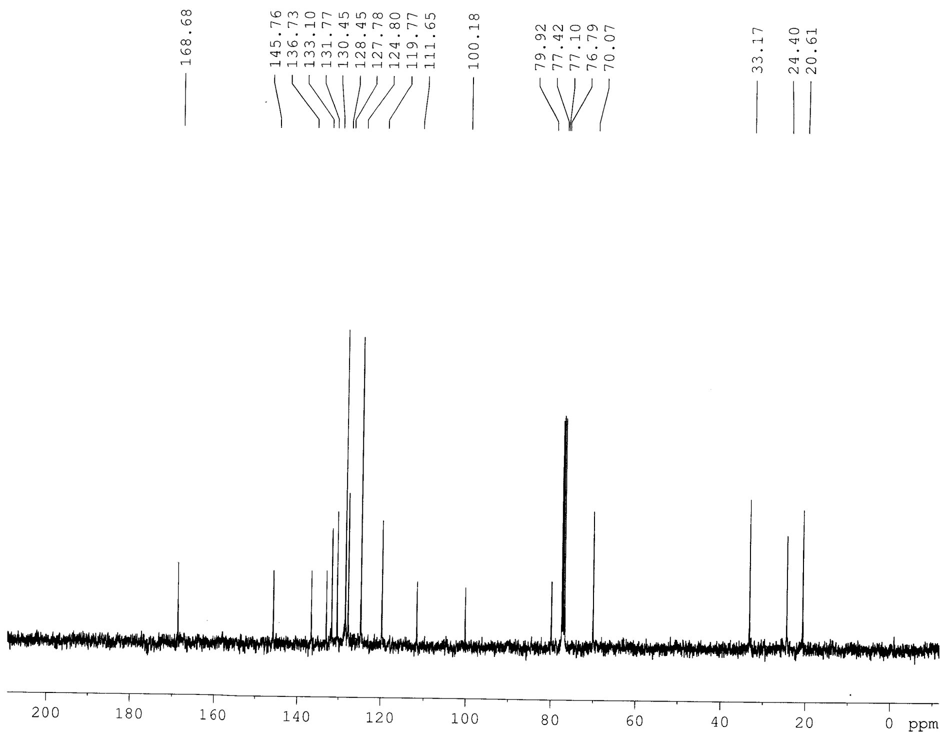

Figure S24: ^{13}C NMR spectrum of compound **1l**.

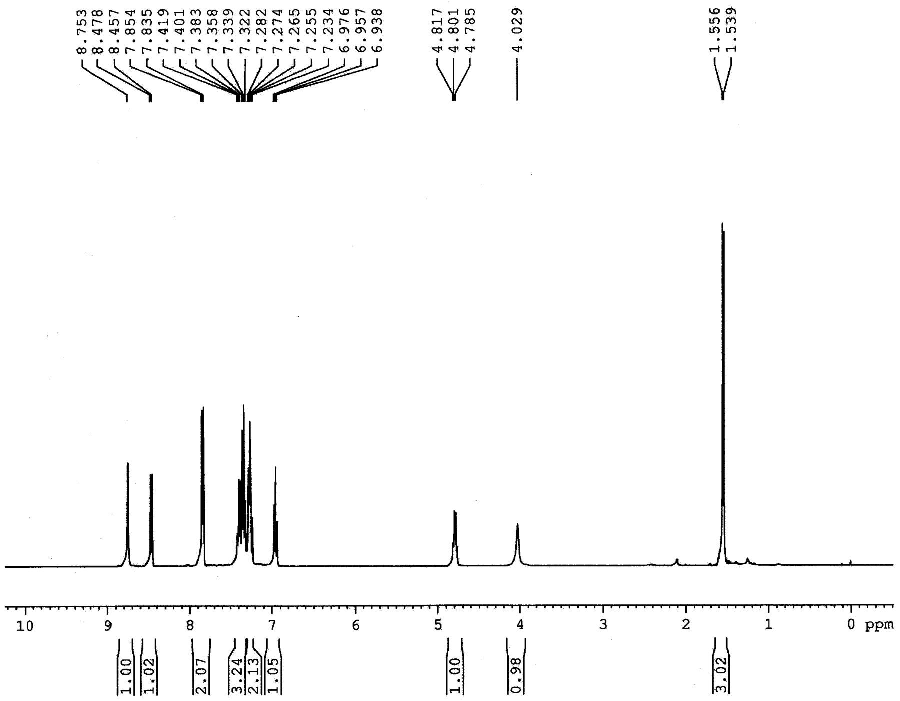

Figure S25: ^{1}H NMR spectrum of compound **1m**.

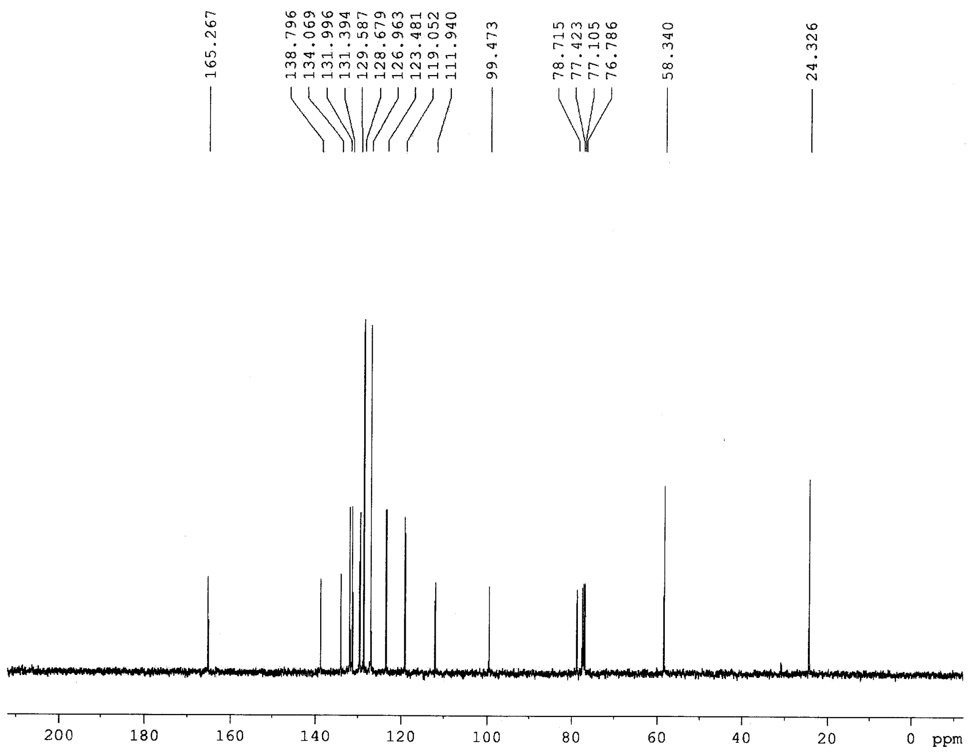

Figure S26: ^{13}C NMR spectrum of compound **1m**.

\

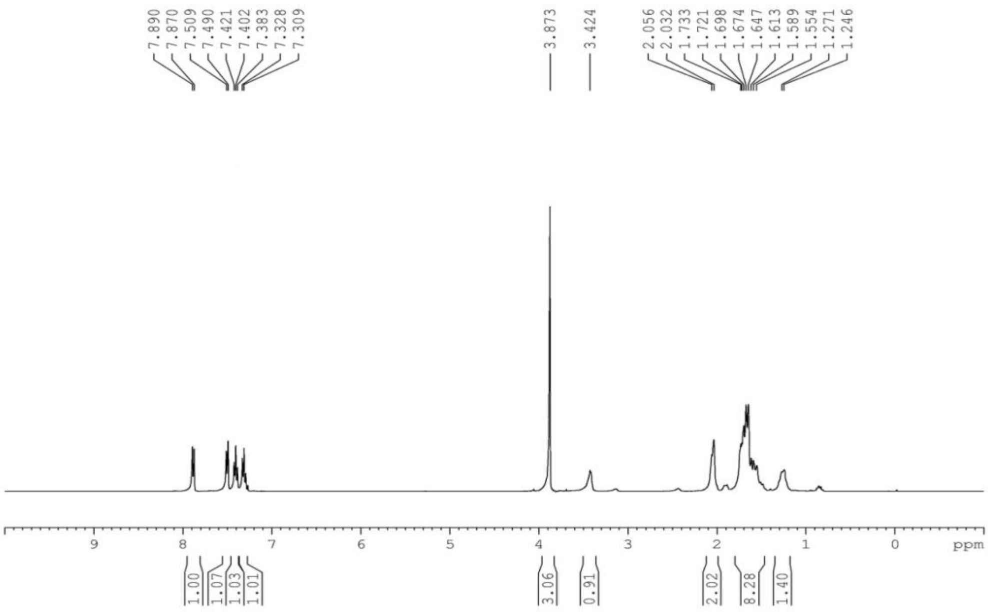

Figure S27: ^{1}H NMR spectrum of compound **2d**.

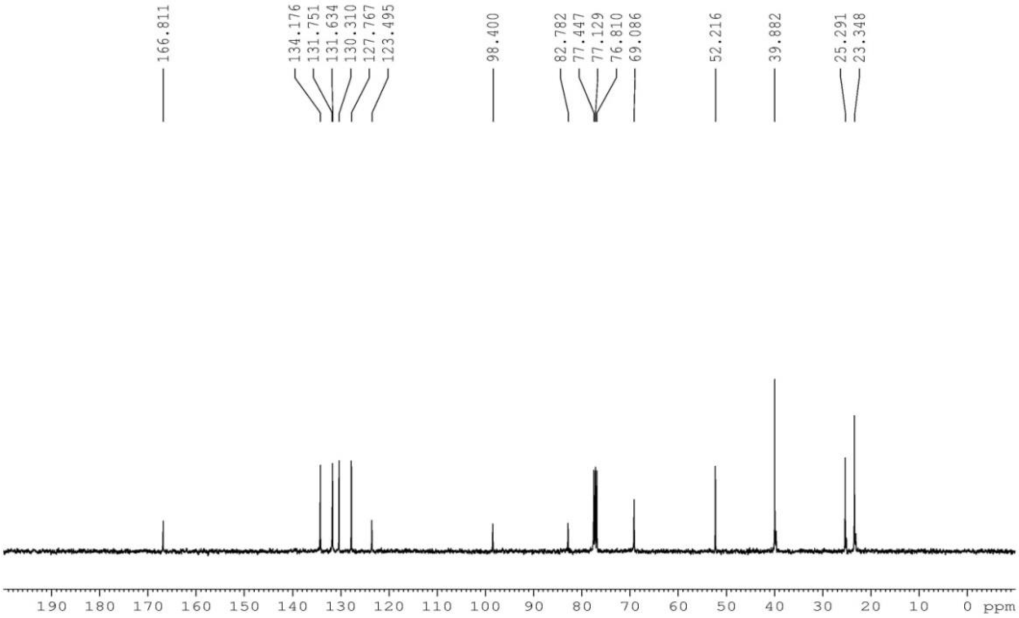

Figure S28: ^{13}C NMR spectrum of compound **2d**.

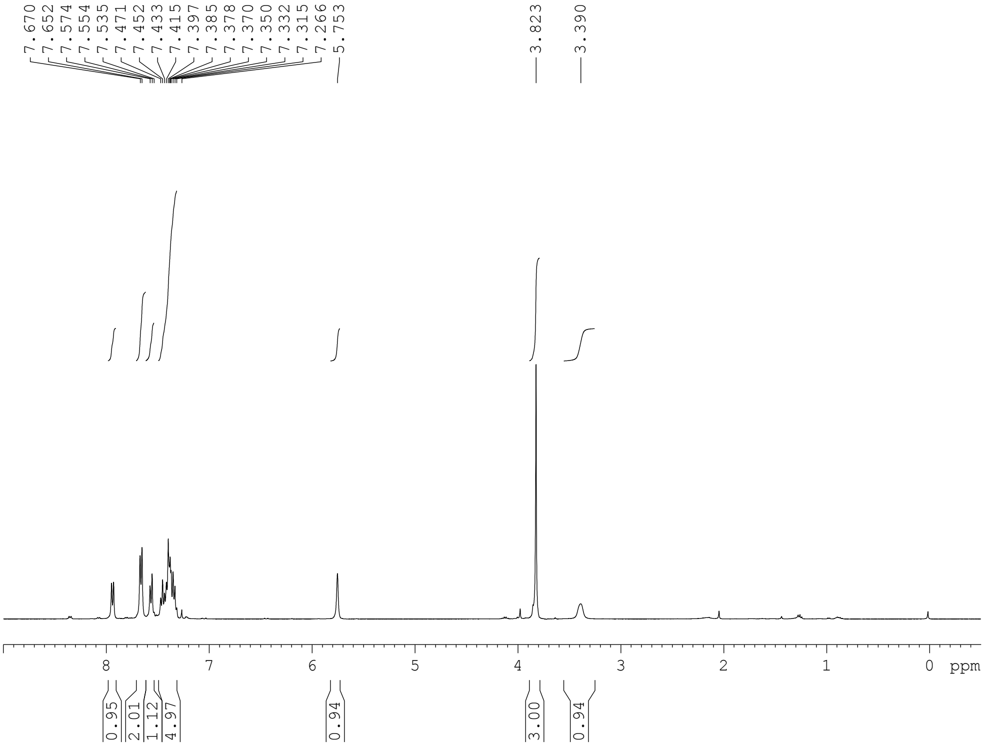

Figure S29: ^{1}H NMR spectrum of compound **2e**.

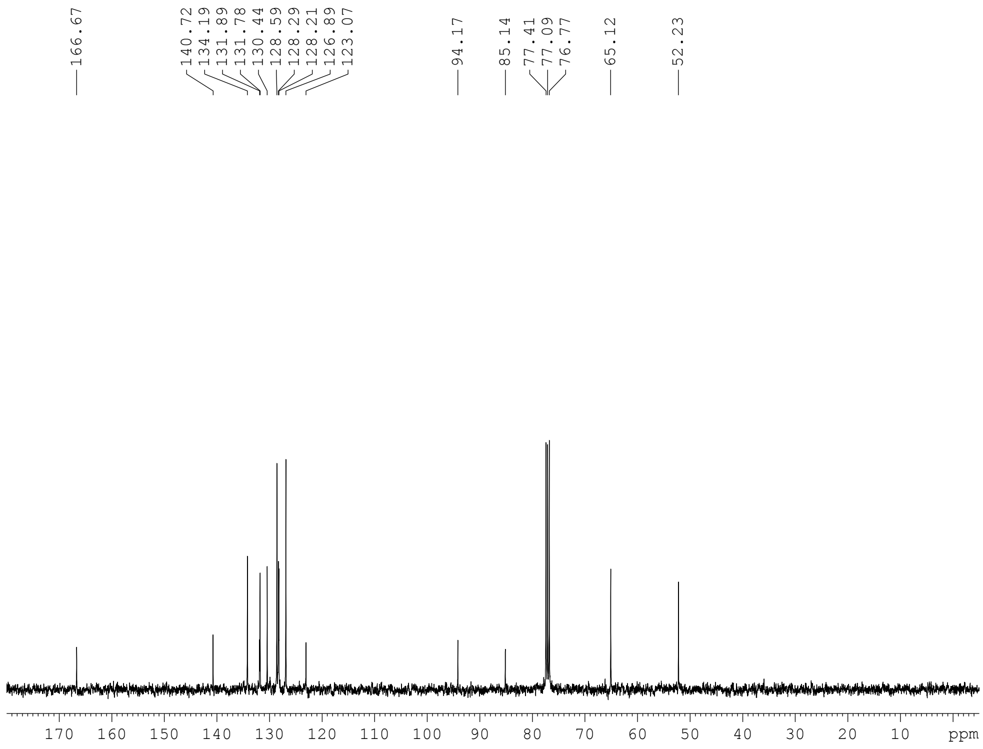

Figure S30: ^{13}C NMR spectrum of compound **2e**.

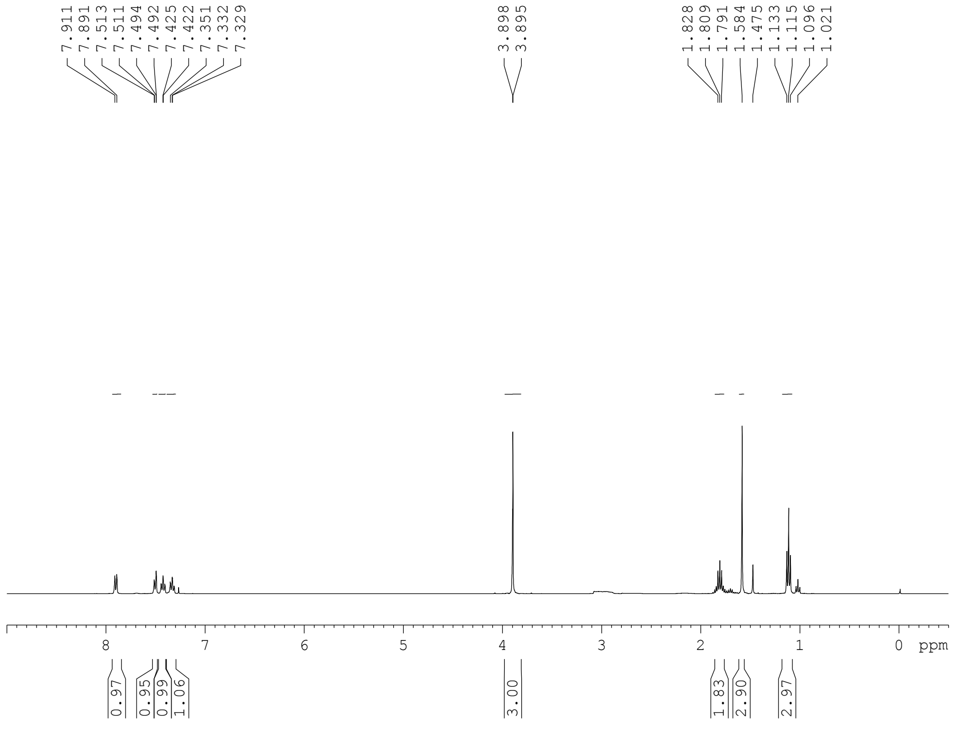

Figure S31: ^{1}H NMR spectrum of compound **2f**.

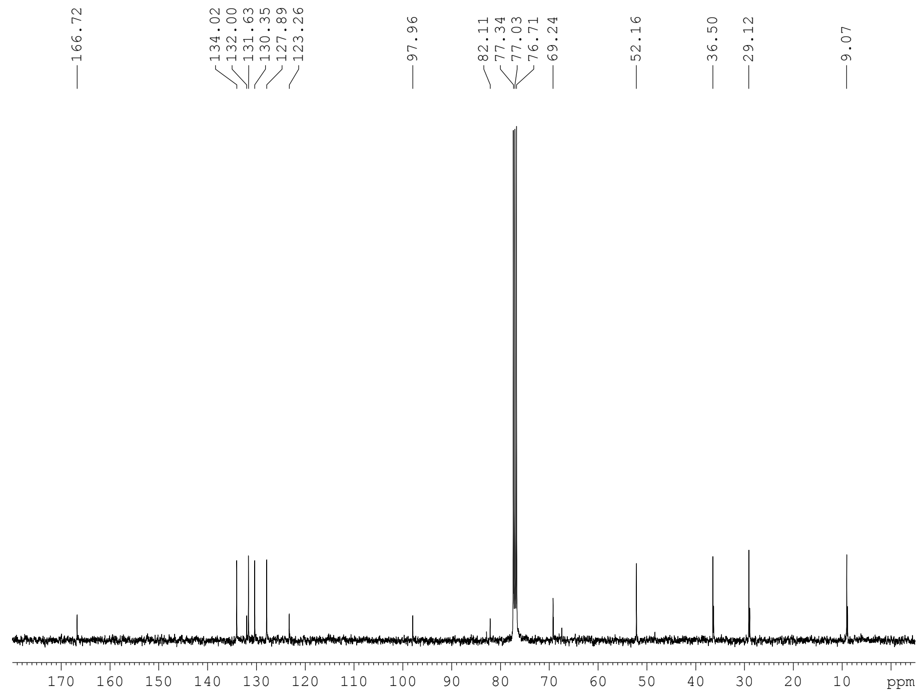

Figure S32: ^{13}C NMR spectrum of compound **2f**.

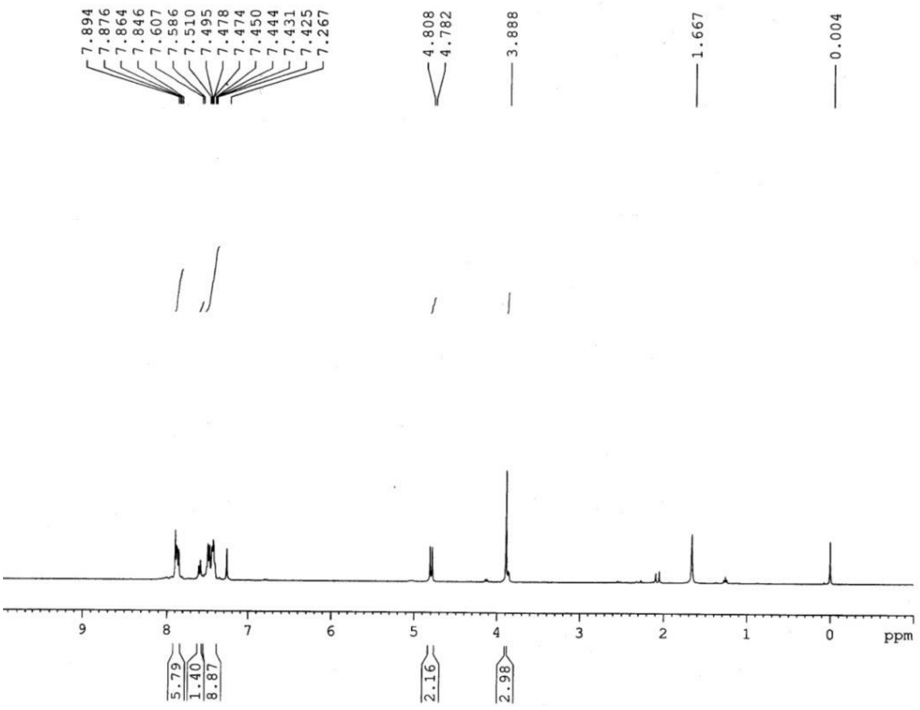

Figure S33: ^{1}H NMR spectrum of compound **2g**.

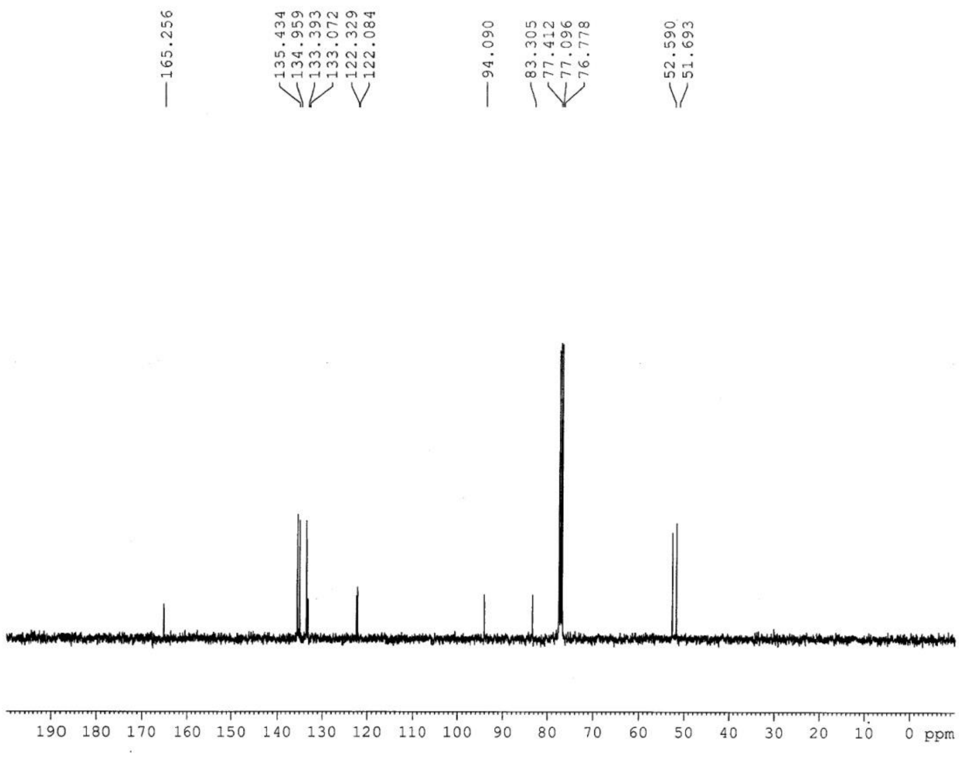

Figure S34: ^{13}C NMR spectrum of compound **2g**.

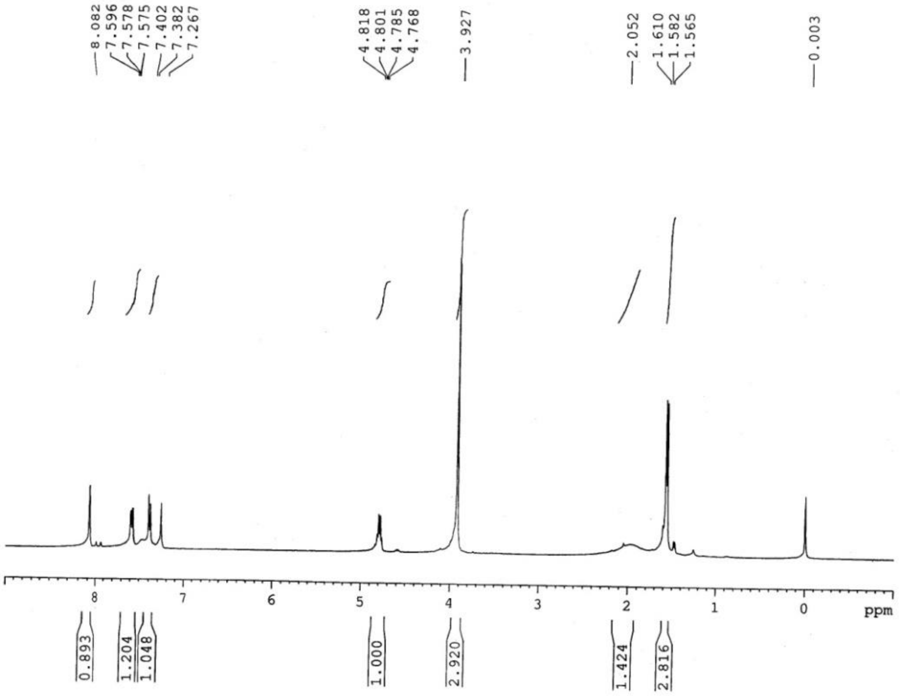

Figure S35: ^{1}H NMR spectrum of compound **2h**.

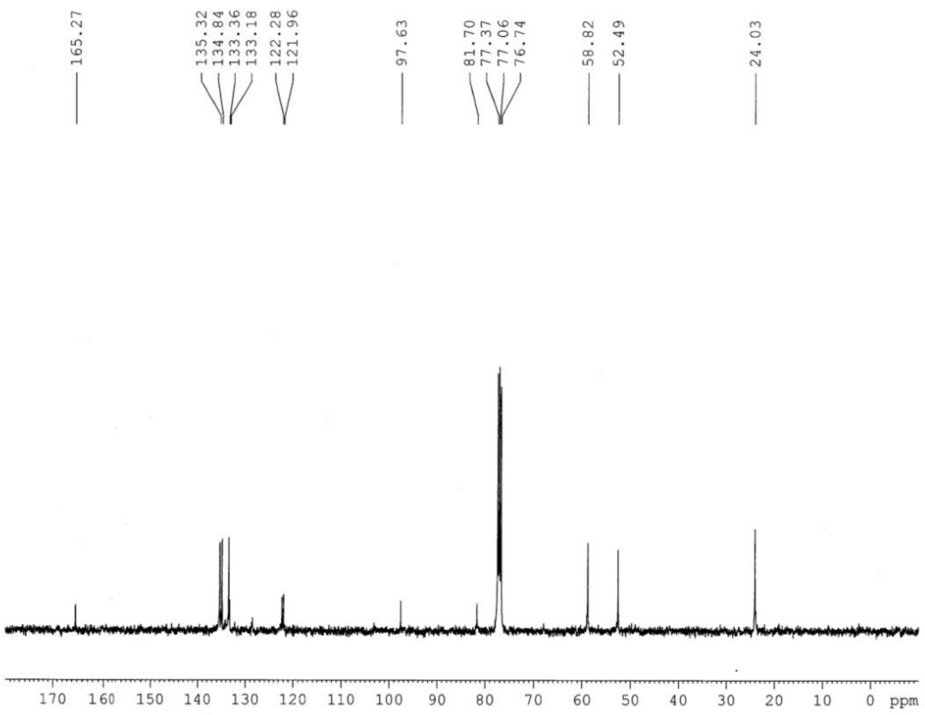

Figure S36: ^{13}C NMR spectrum of compound **2h**.

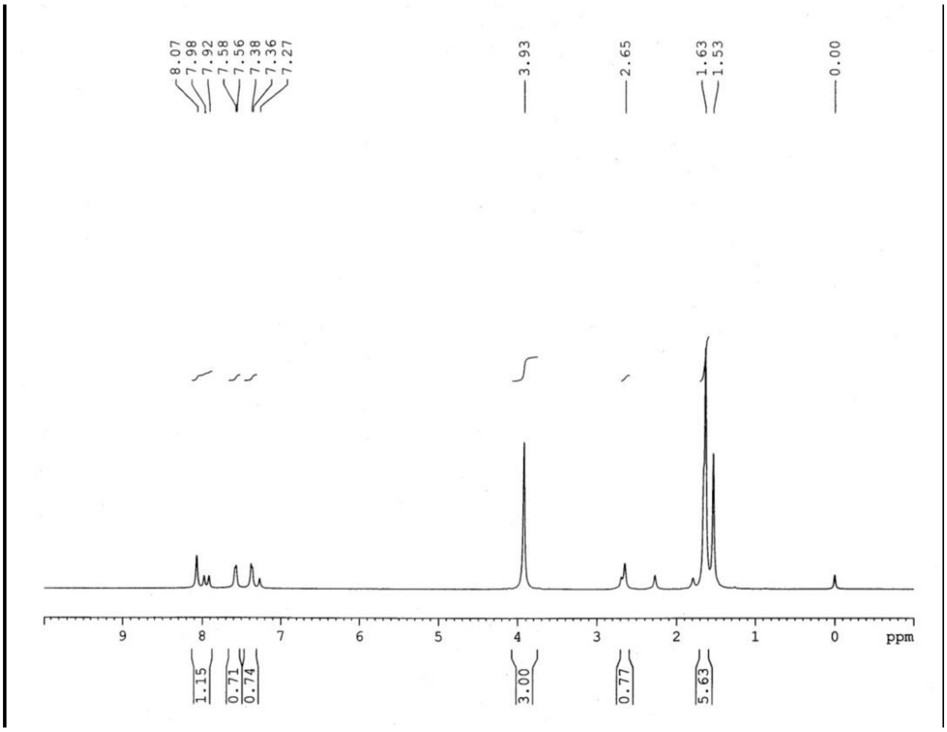

Figure S37: ^{1}H NMR spectrum of compound **2i**.

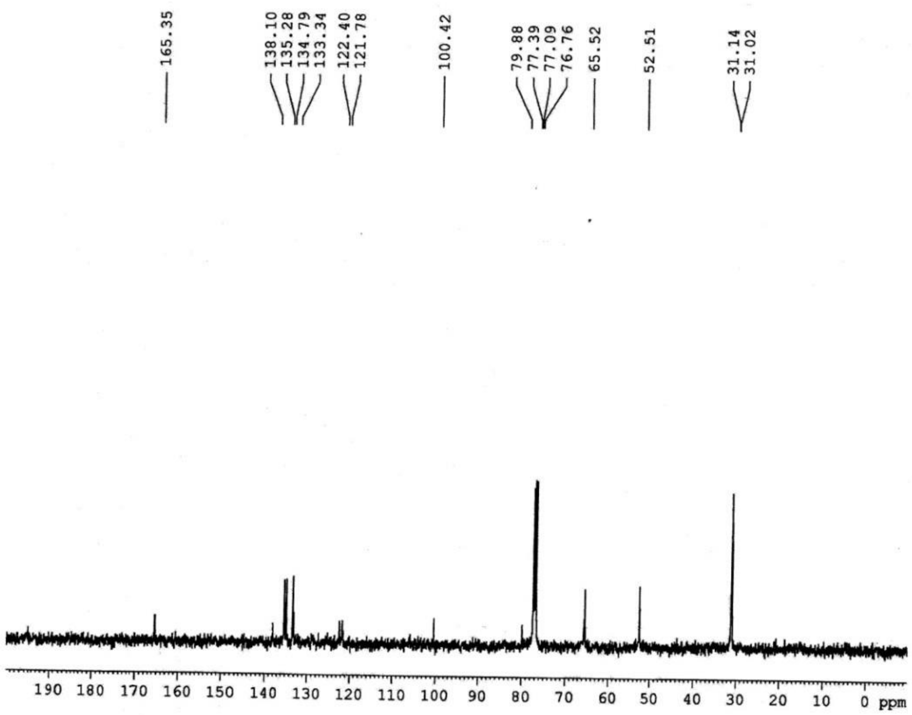

Figure S38: ^{13}C NMR spectrum of compound **2i**.

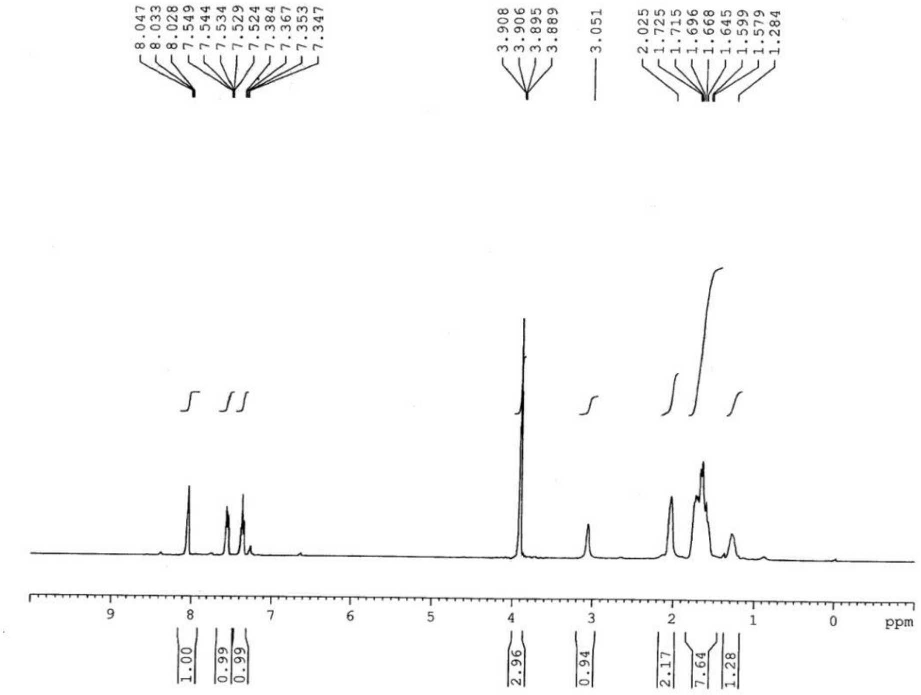

Figure S39: ^{1}H NMR spectrum of compound **2j**.

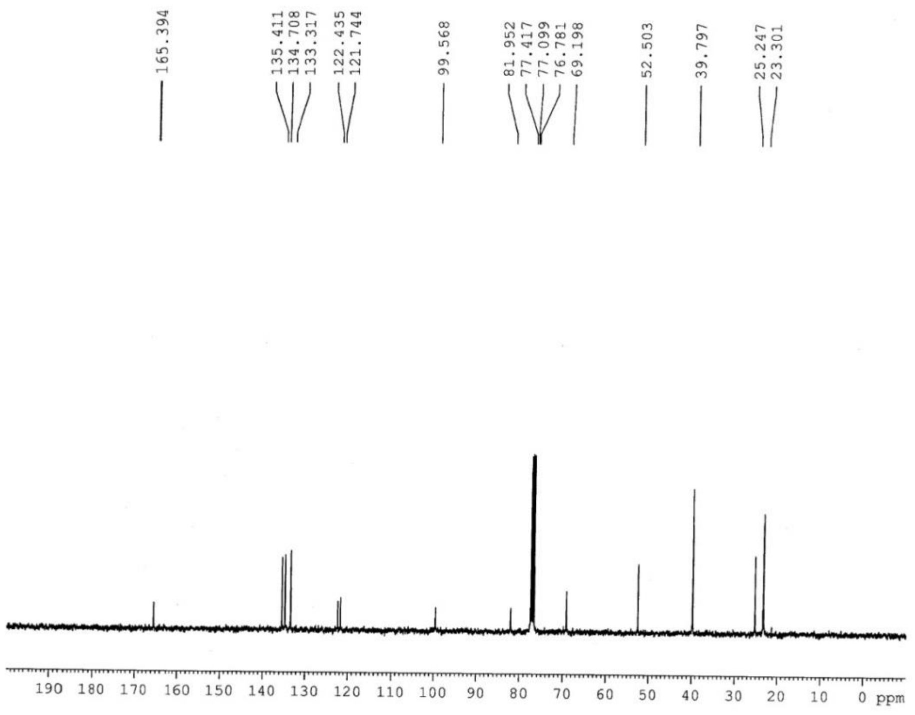

Figure S40: ^{13}C NMR spectrum of compound **2j**.

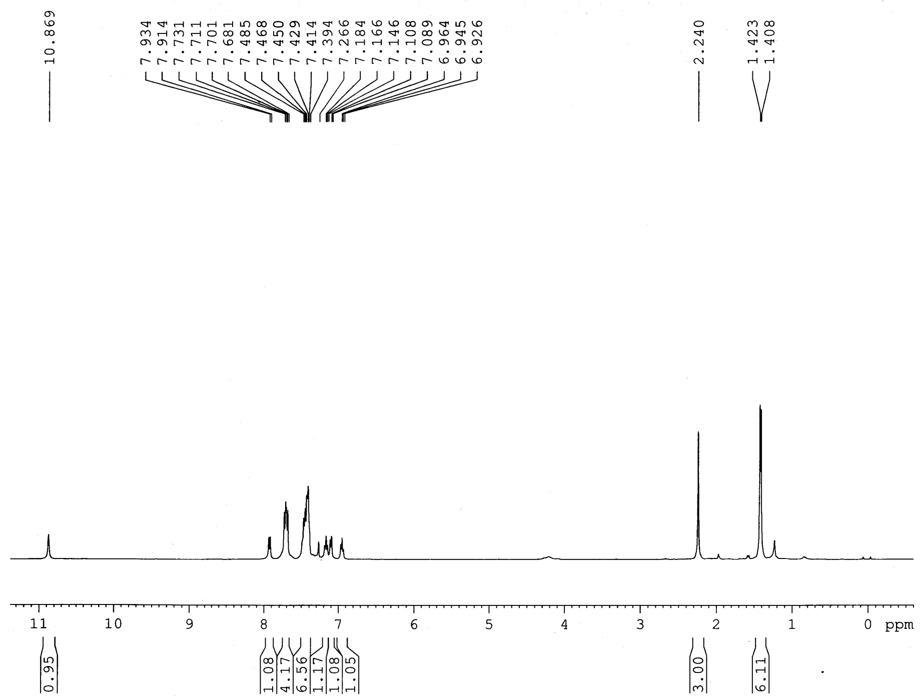

Figure S41: ^{1}H NMR spectrum of compound **3a**.

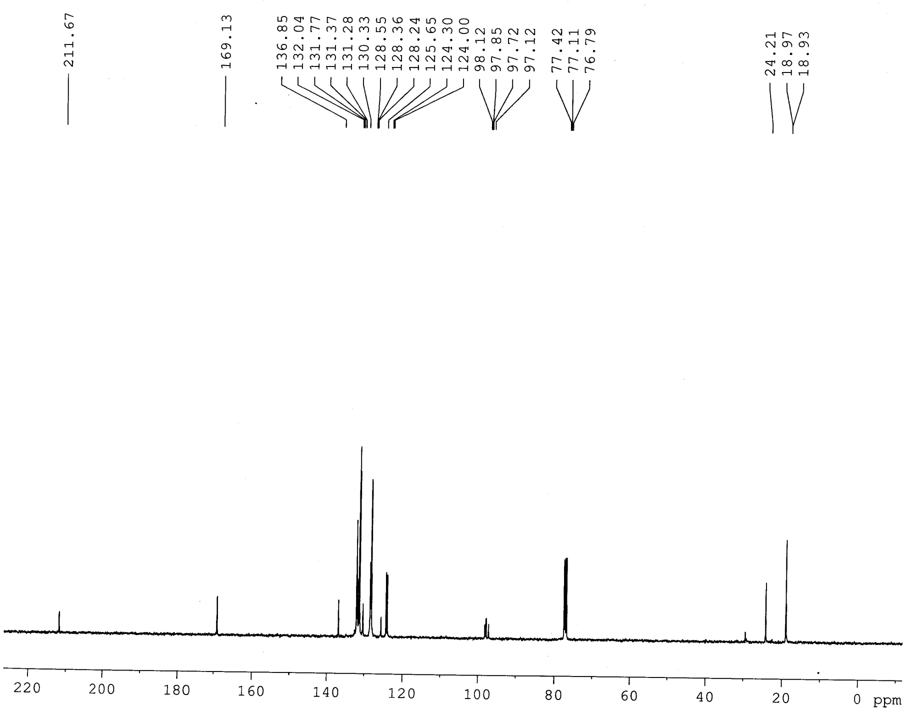

Figure S42: ^{13}C NMR spectrum of compound **3a**.

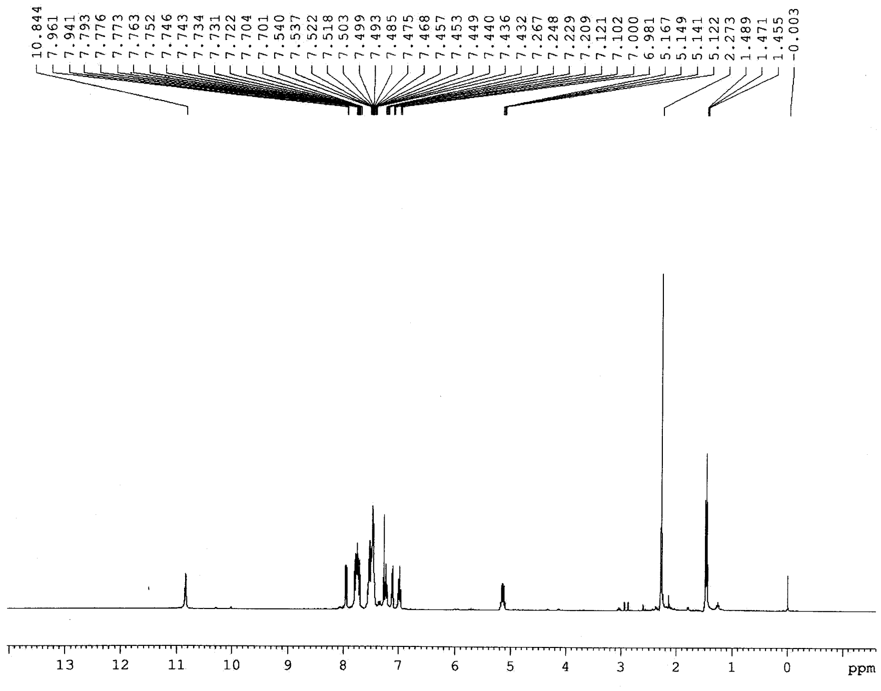

Figure S43: ^{1}H NMR spectrum of compound **3b**.

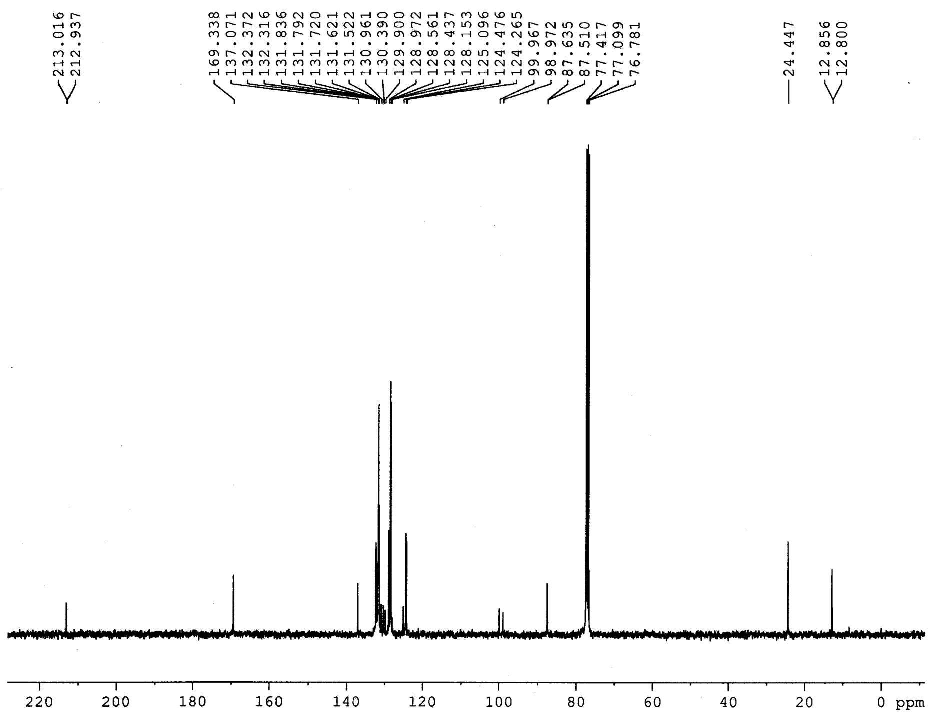

Figure S44: ^{13}C NMR spectrum of compound **3b**.

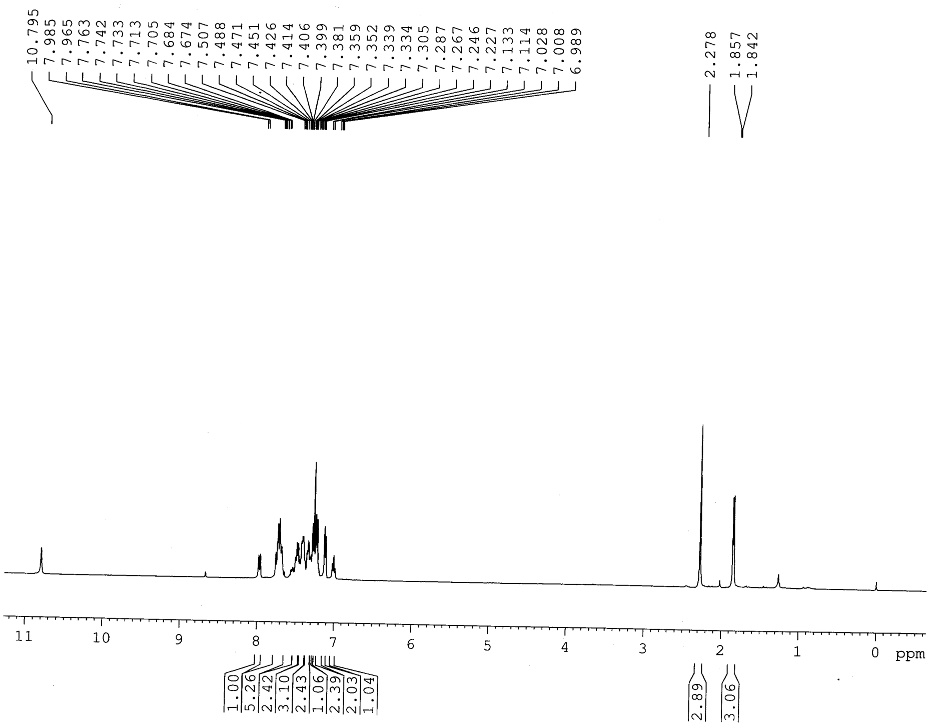

Figure S45: ^{1}H NMR spectrum of compound **3c**.

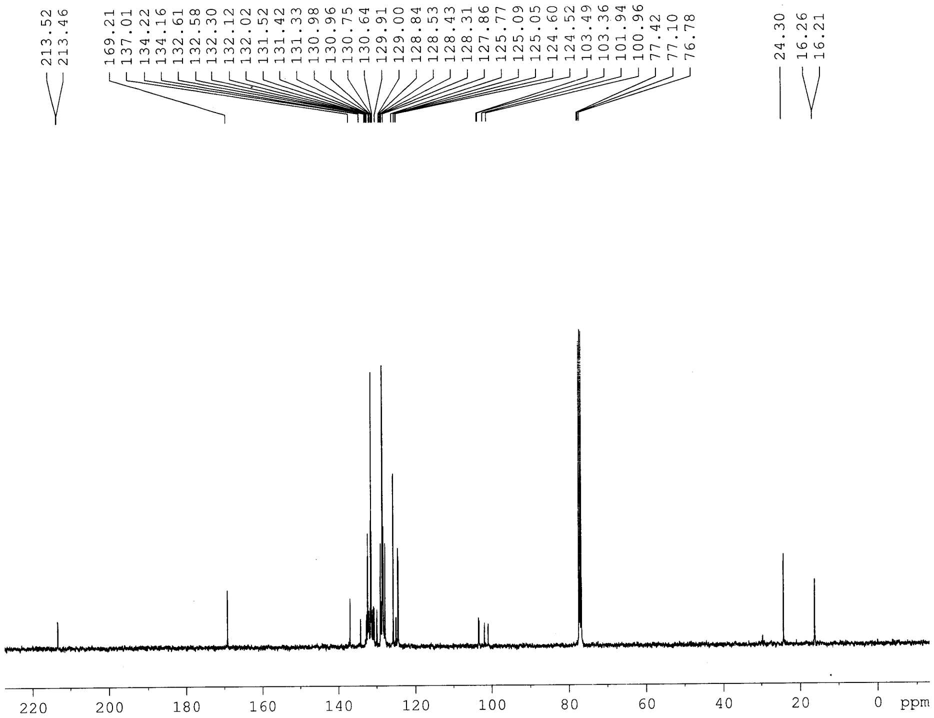

Figure S46: ^{13}C NMR spectrum of compound **3c**.

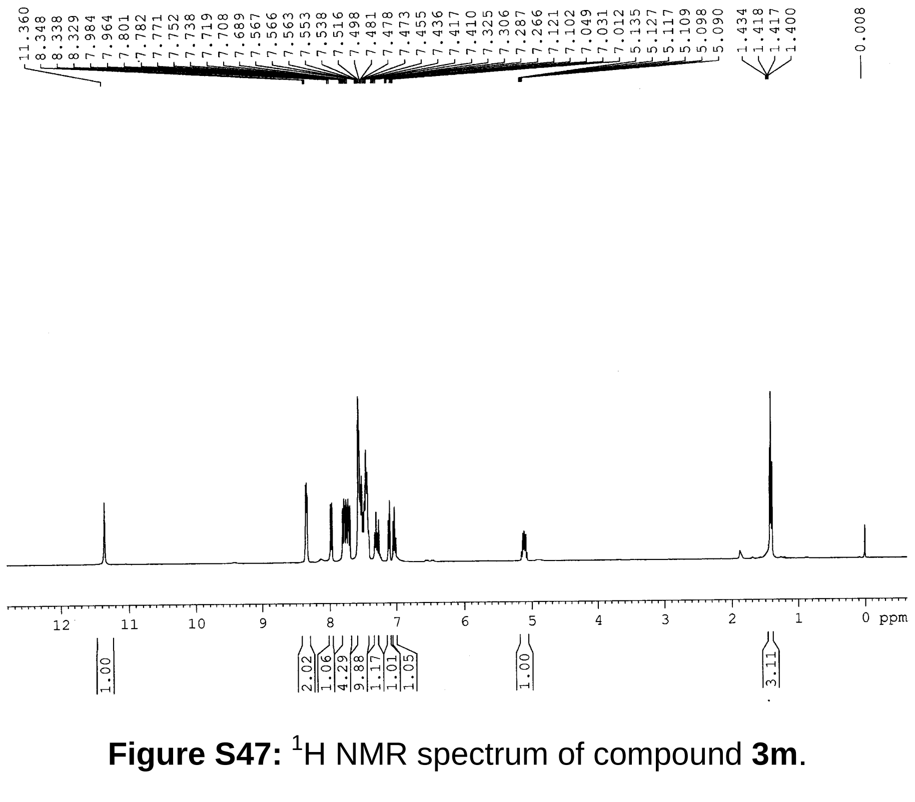

Figure S47: ^{1}H NMR spectrum of compound **3m**.

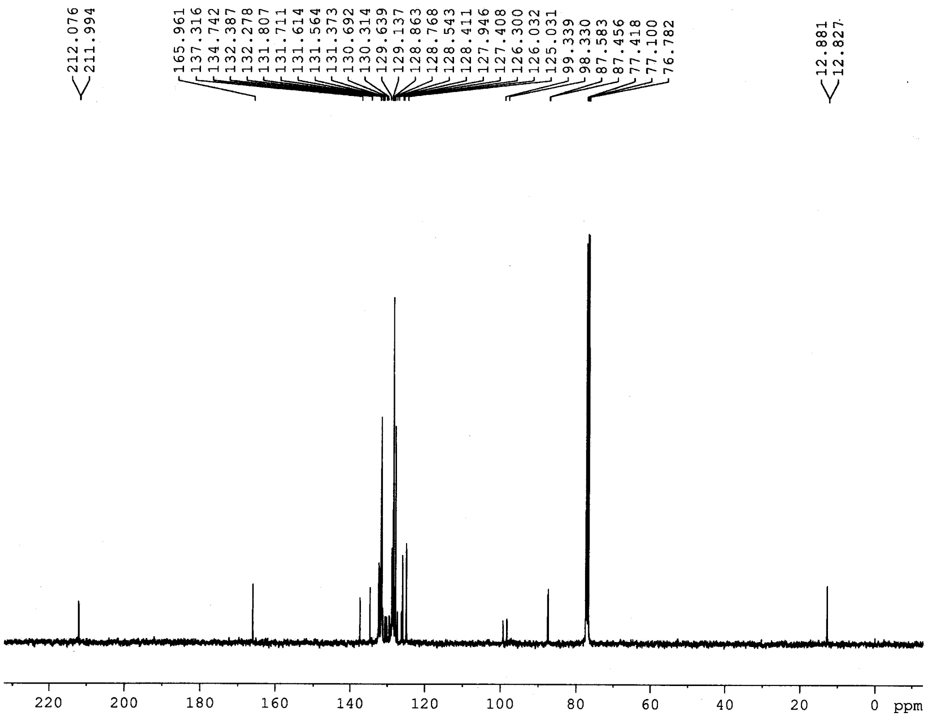

Figure S48: ^{13}C NMR spectrum of compound **3m**.

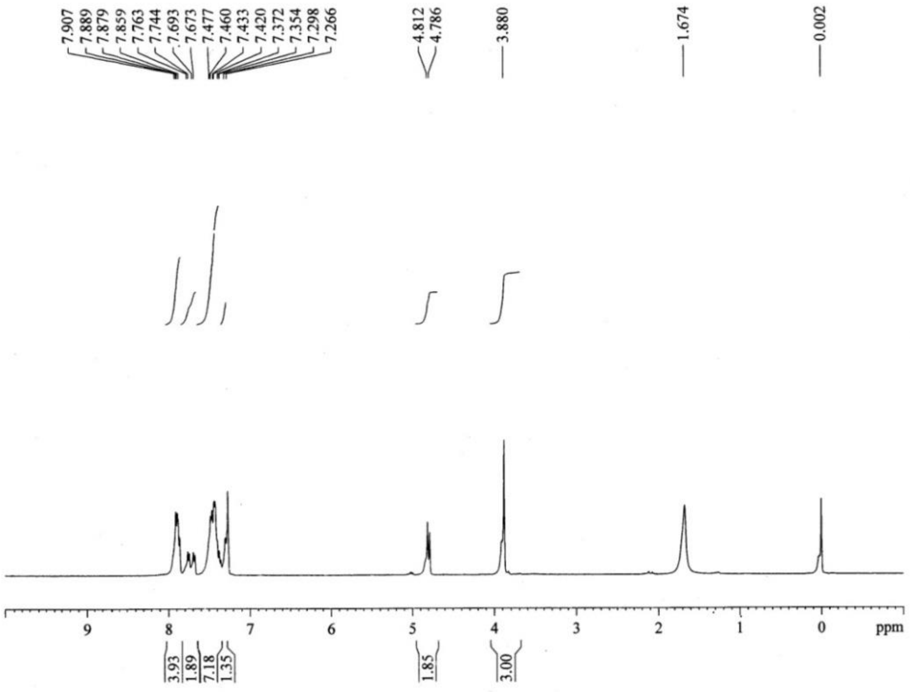

Figure S49: ^{1}H NMR spectrum of compound **4a**.

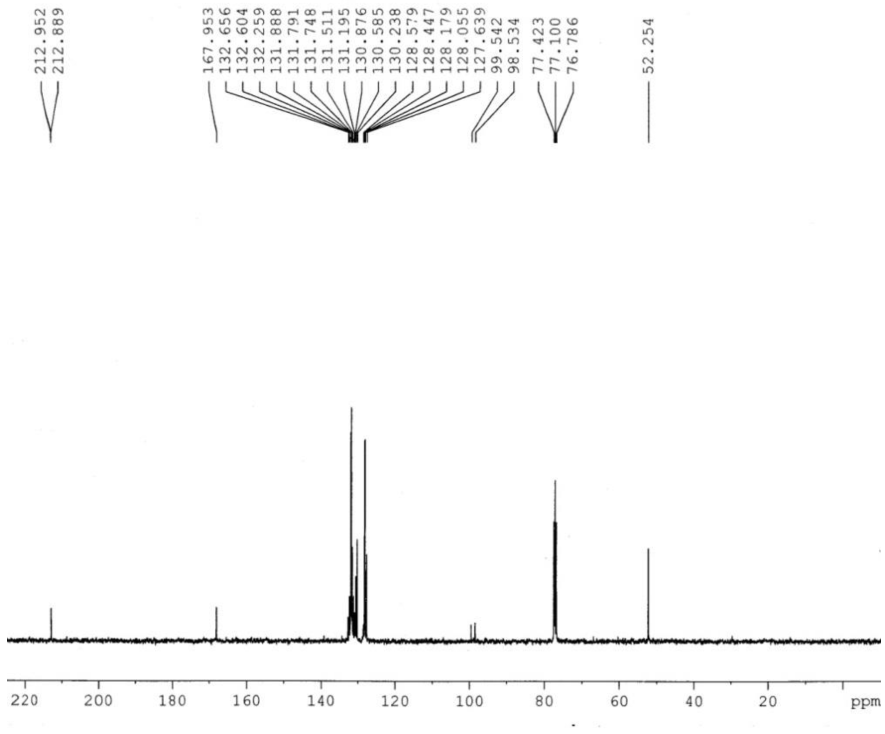

Figure S50: ^{13}C NMR spectrum of compound **4a**.

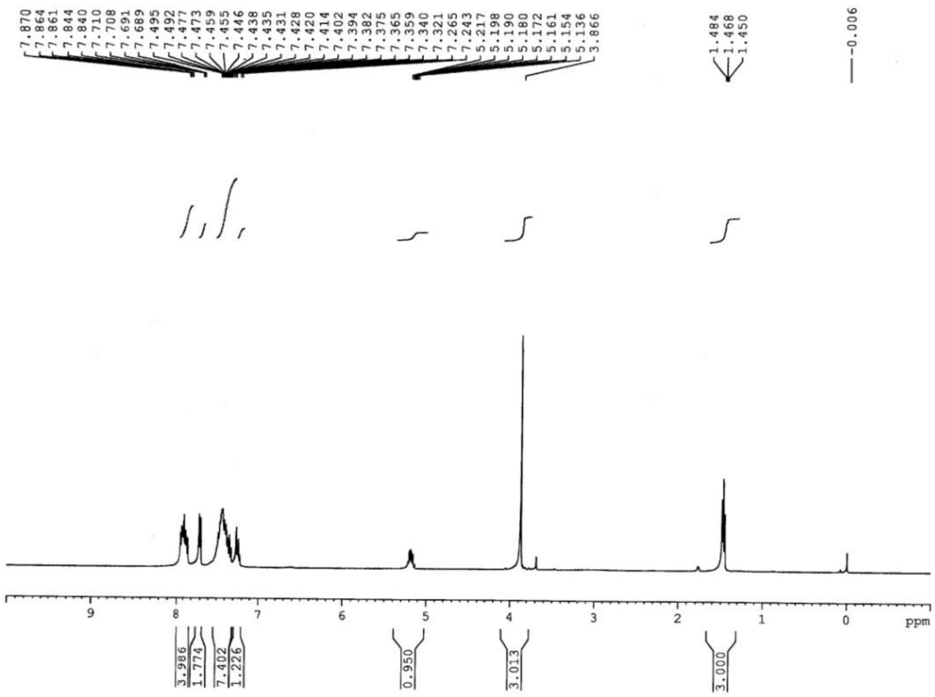

Figure S51: ^{1}H NMR spectrum of compound **4b**.

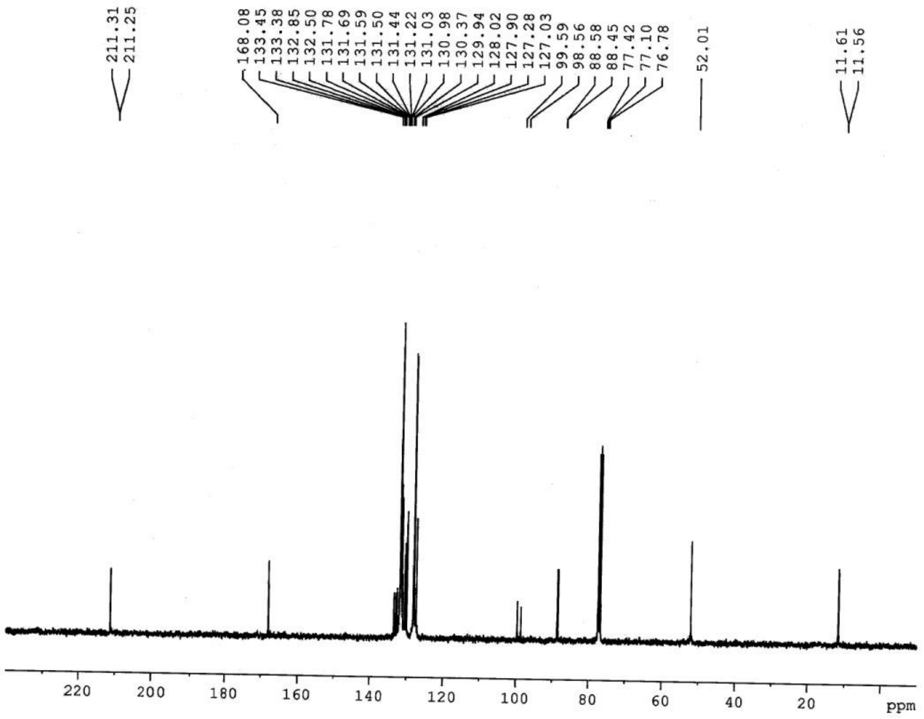

Figure S52: ^{13}C NMR spectrum of compound **4b**.

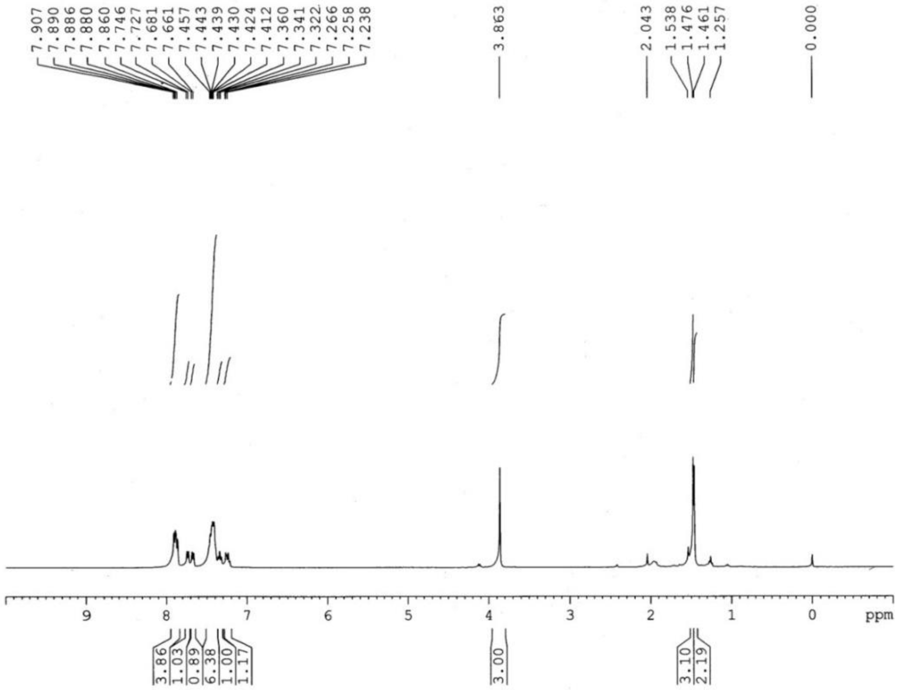

Figure S53: ^{1}H NMR spectrum of compound **4c**.

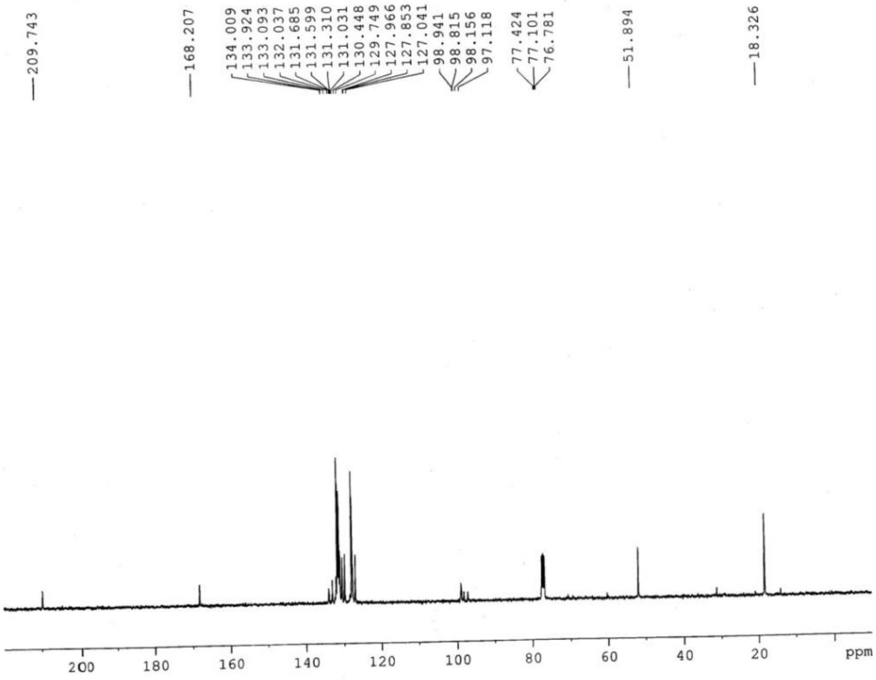

Figure S54: ^{13}C NMR spectrum of compound **4c**.

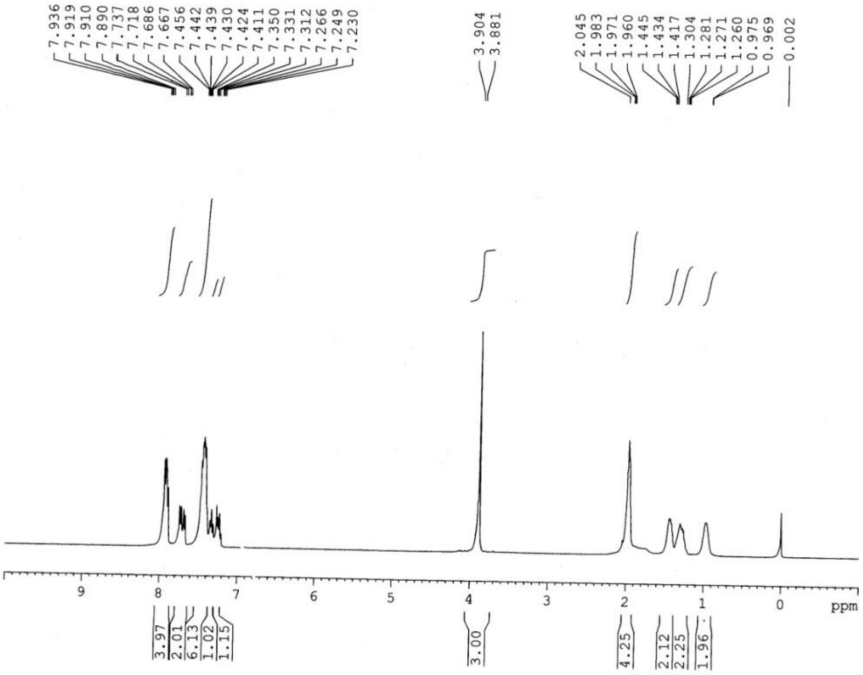

Figure S55: ^{1}H NMR spectrum of compound **4d**.

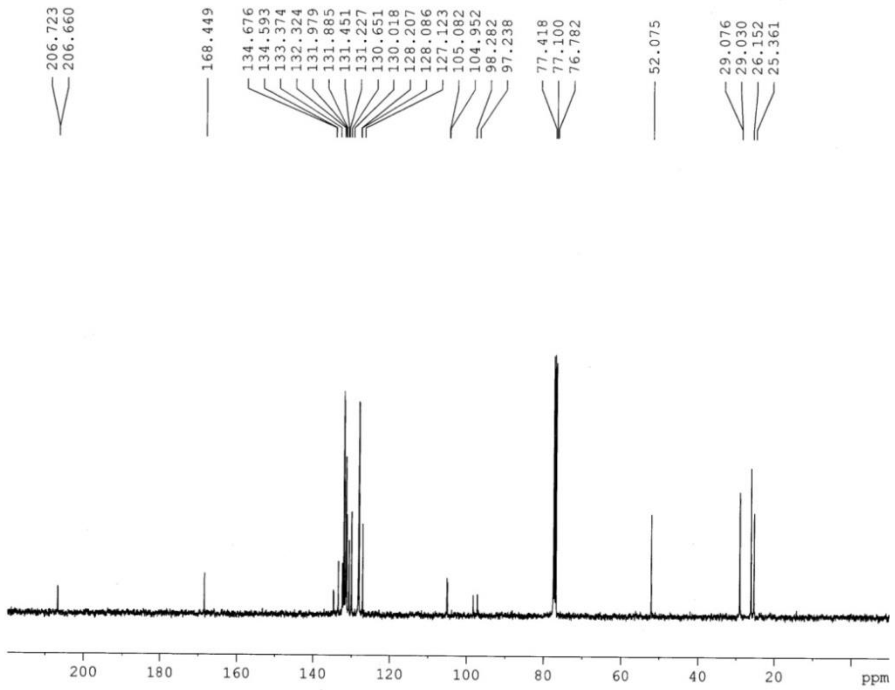

Figure S56: ^{13}C NMR spectrum of compound **4d**.

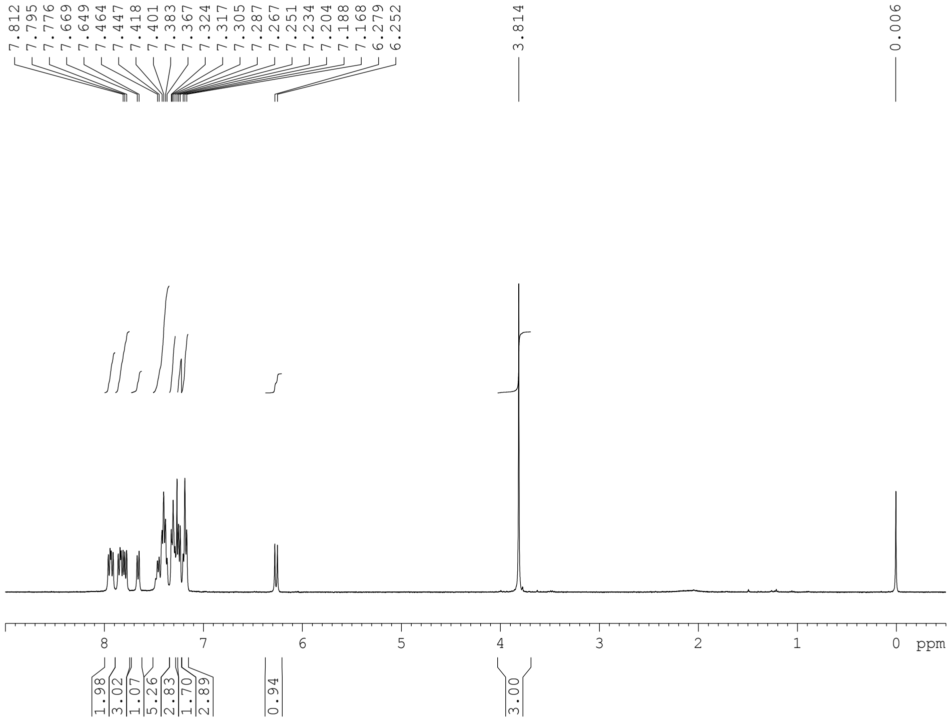

Figure S57: ^{1}H NMR spectrum of compound **4e**.

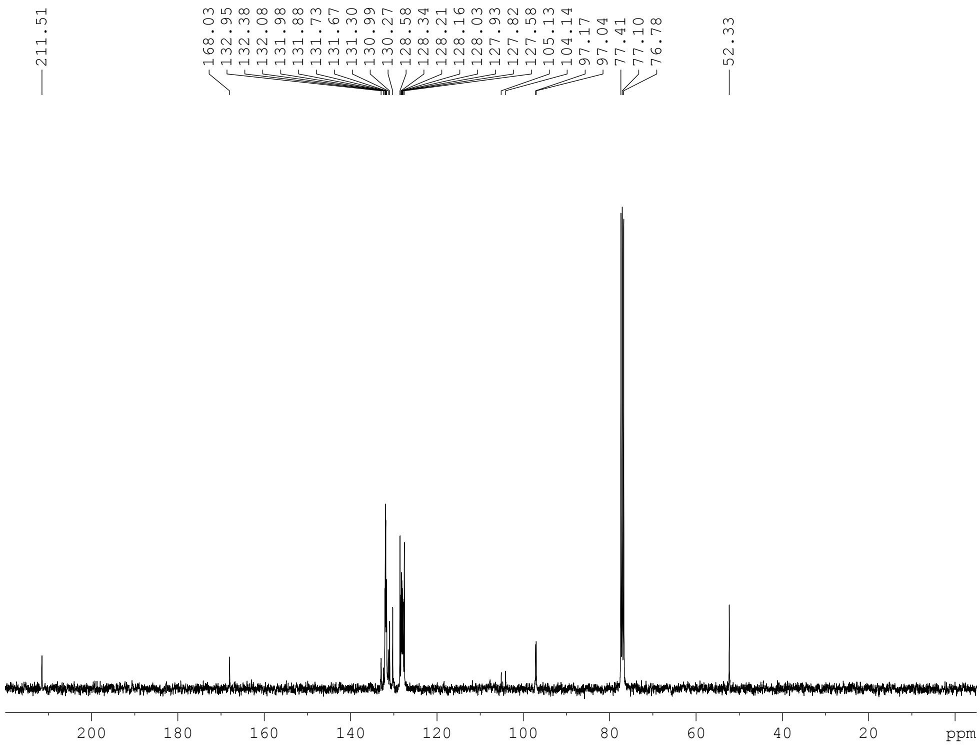

Figure S58: ^{13}C NMR spectrum of compound **4e**.

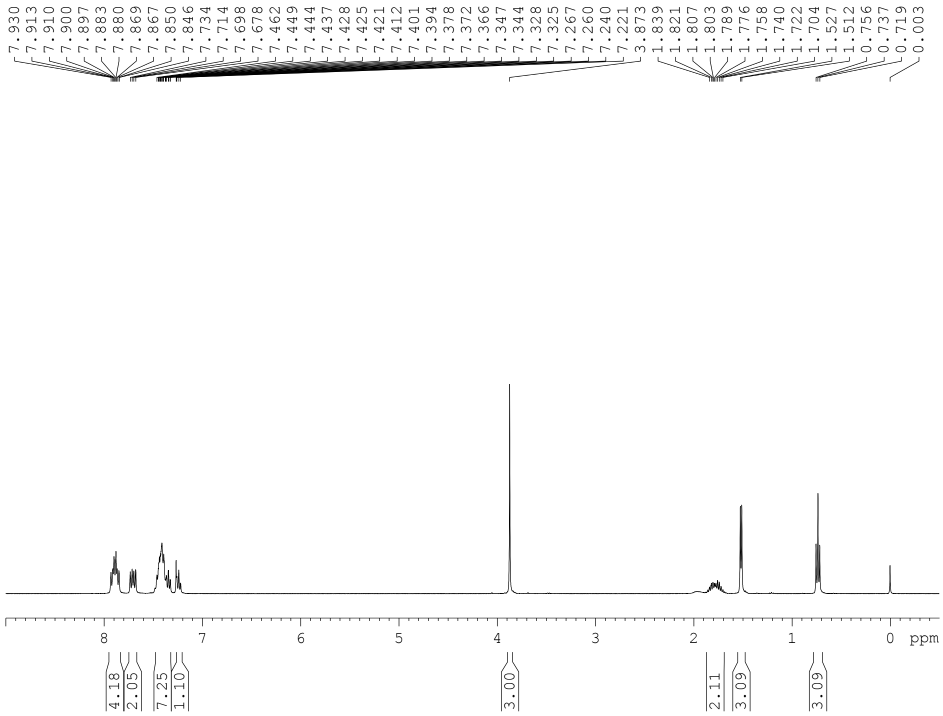

Figure S59: ^{1}H NMR spectrum of compound **4f**.

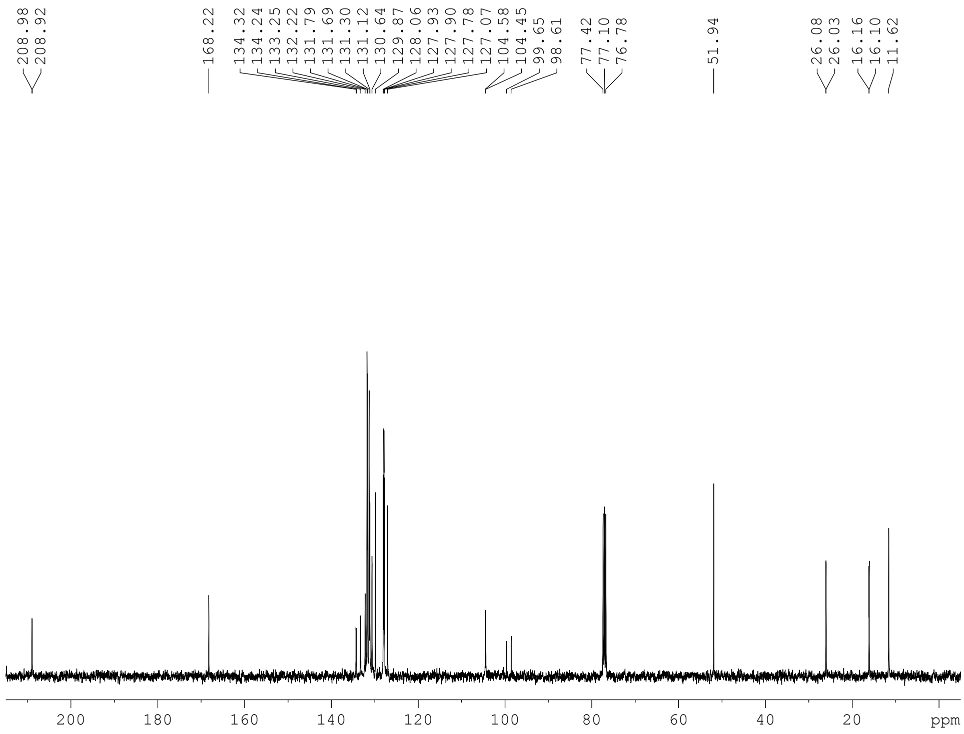

Figure S60: ^{13}C NMR spectrum of compound **4f**.

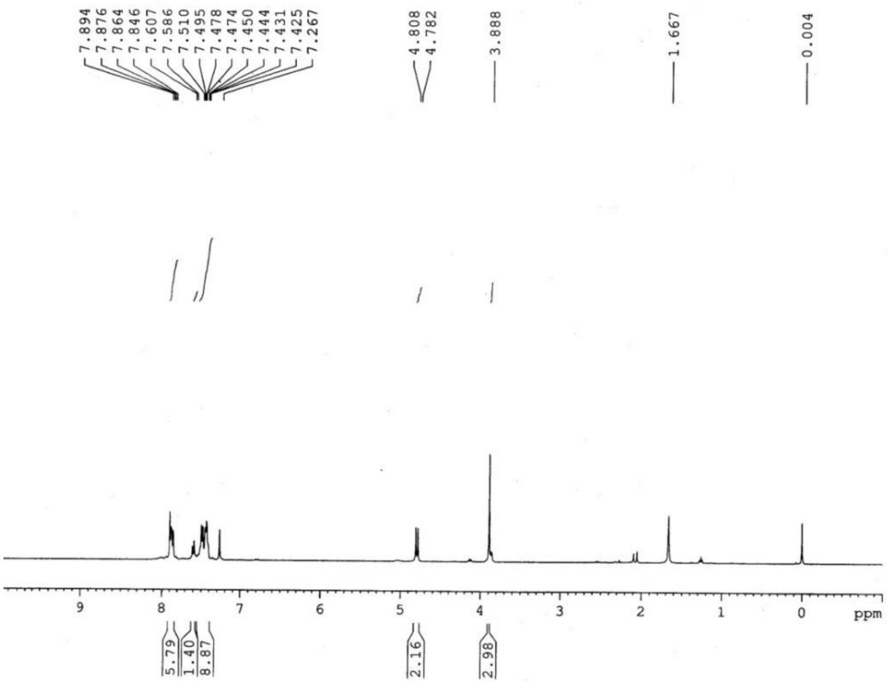

Figure S61: ^{1}H NMR spectrum of compound **4g**.

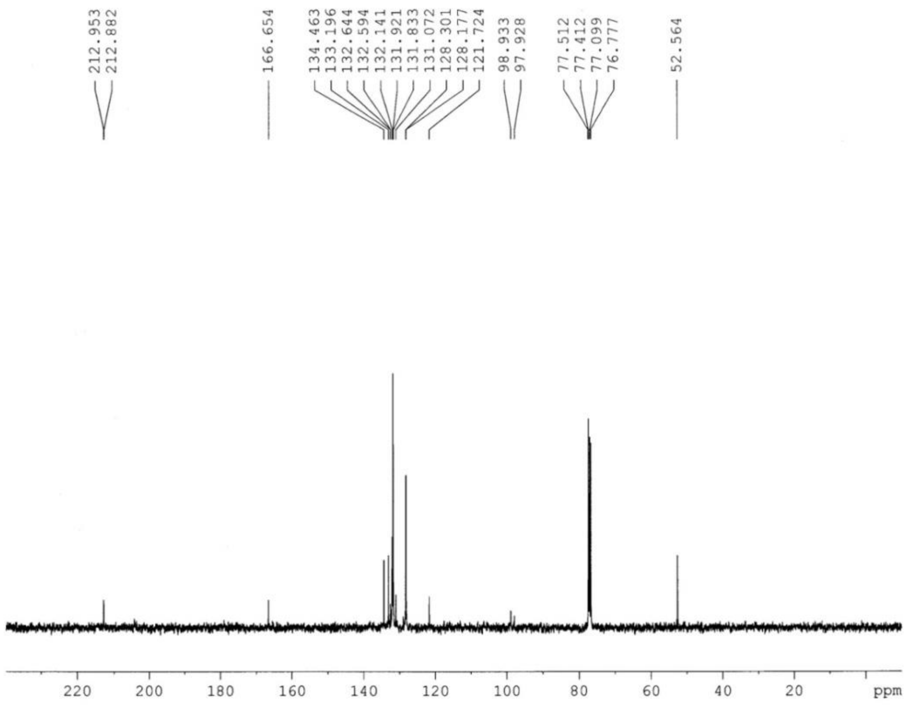

Figure S62: ^{13}C NMR spectrum of compound **4g**.

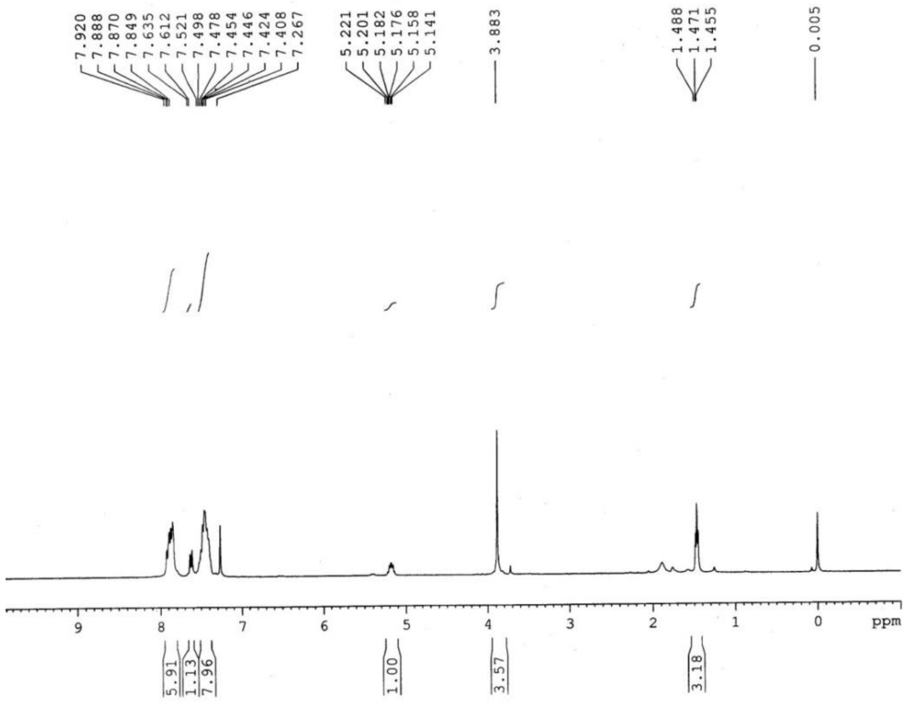

Figure S63: ^{1}H NMR spectrum of compound **4h**.

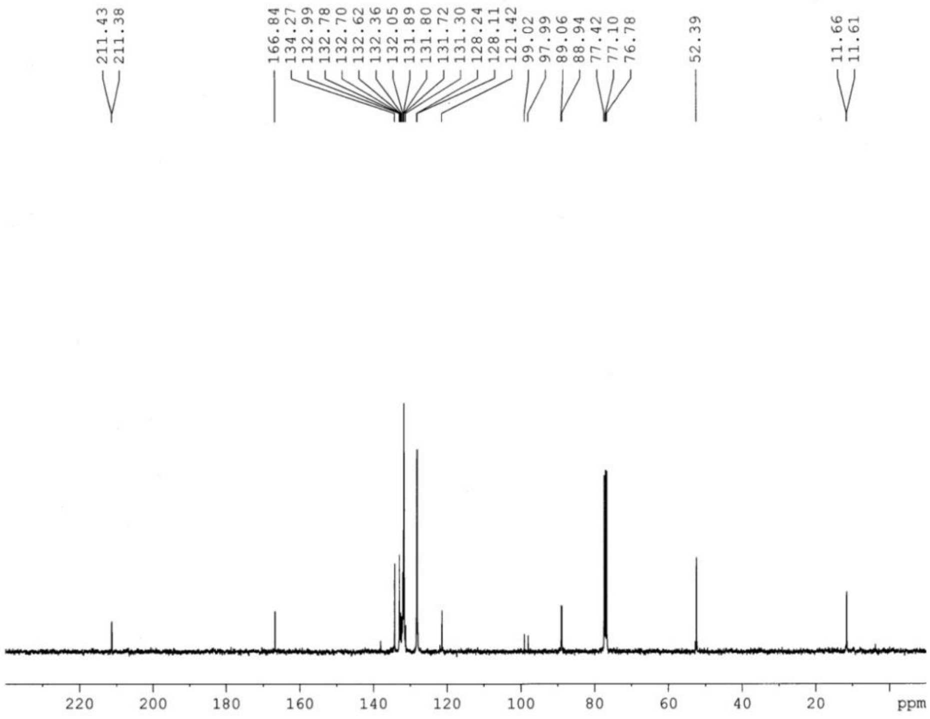

Figure S64: ^{13}C NMR spectrum of compound **4h**.

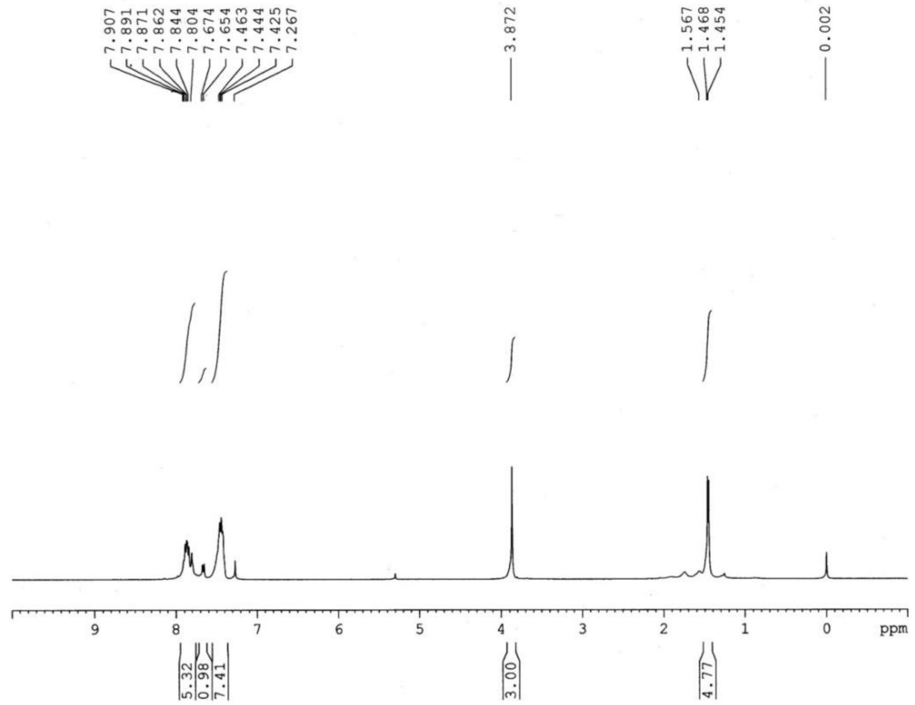

Figure S65: ^{1}H NMR spectrum of compound **4i**.

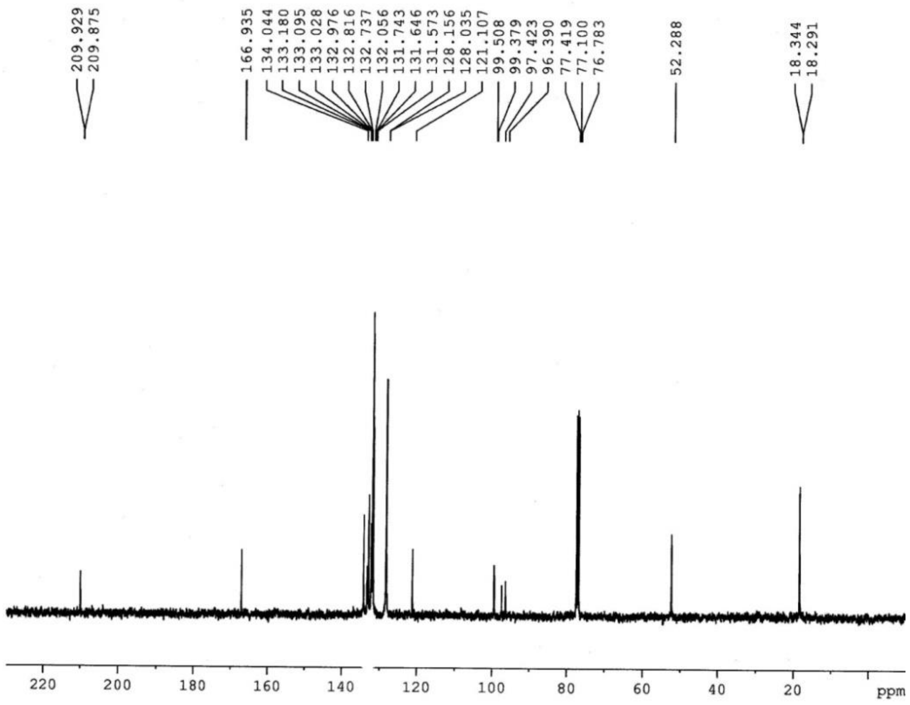

Figure S66: ^{13}C NMR spectrum of compound **4i**.

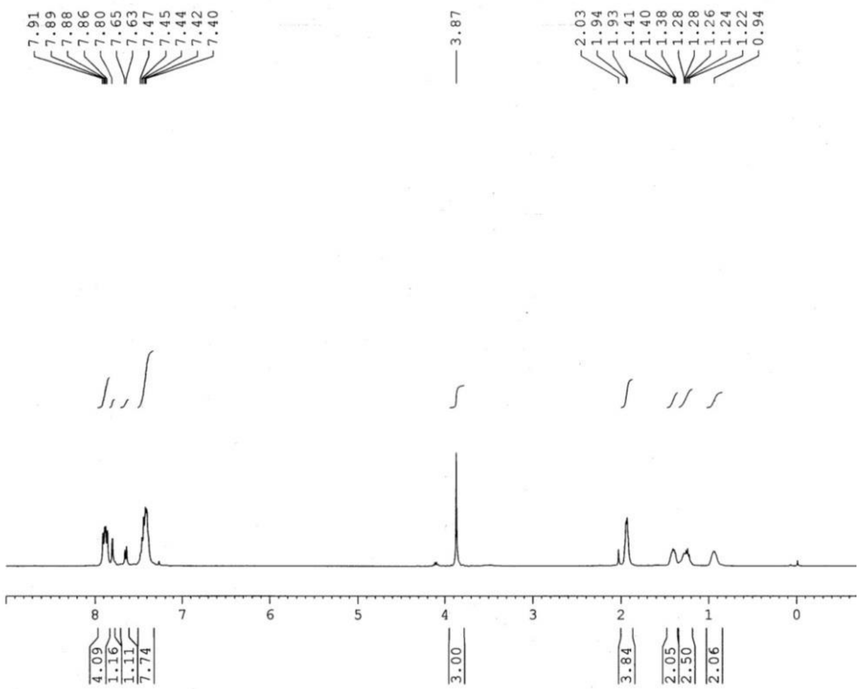

Figure S67: ^{1}H NMR spectrum of compound **4j**.

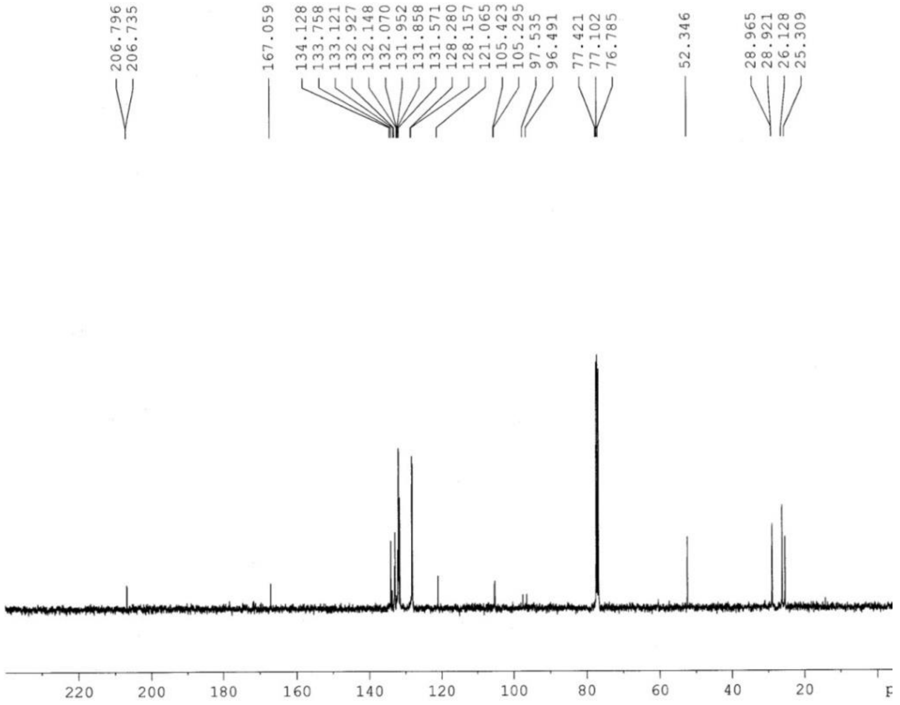

Figure S68: ^{13}C NMR spectrum of compound **4j**.

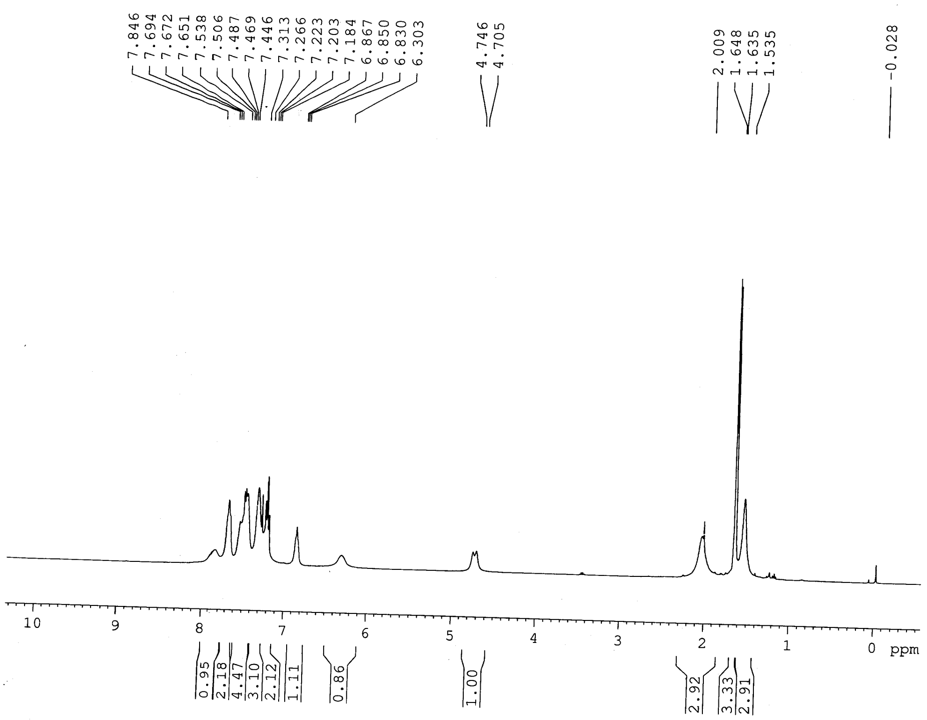

Figure S69: ^{1}H NMR spectrum of compound **5**.

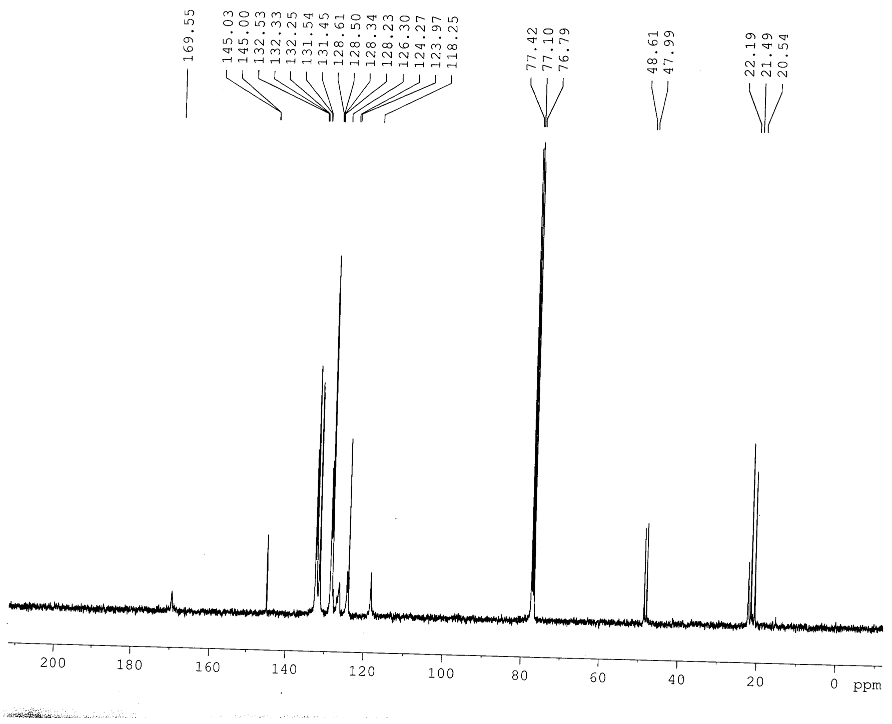

Figure S70: ^{13}C NMR spectrum of compound **5**.

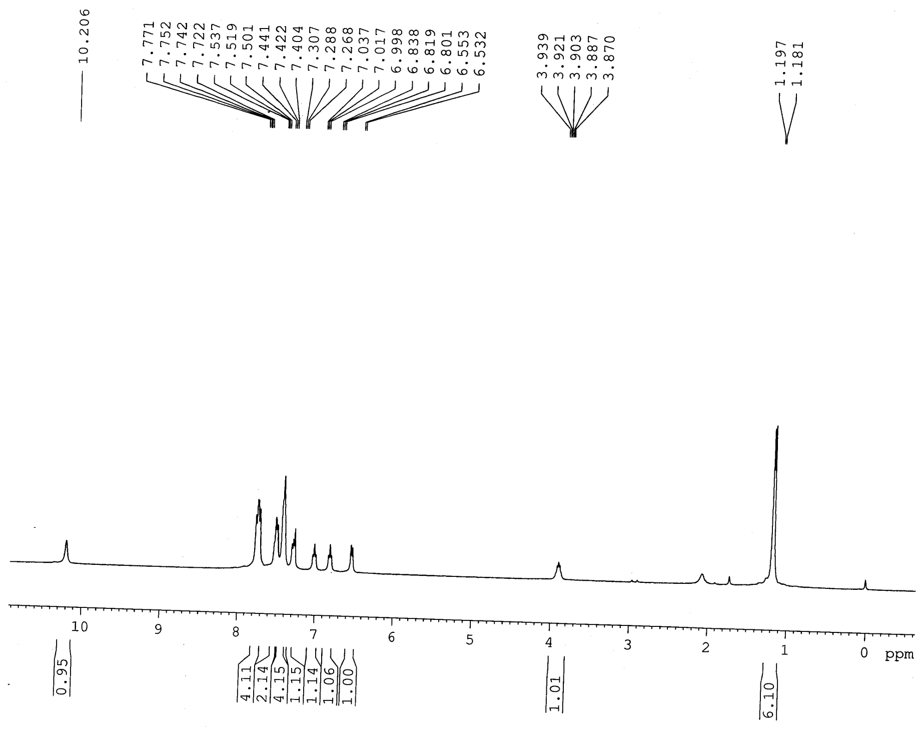

Figure S71: ^{1}H NMR spectrum of compound **6**.

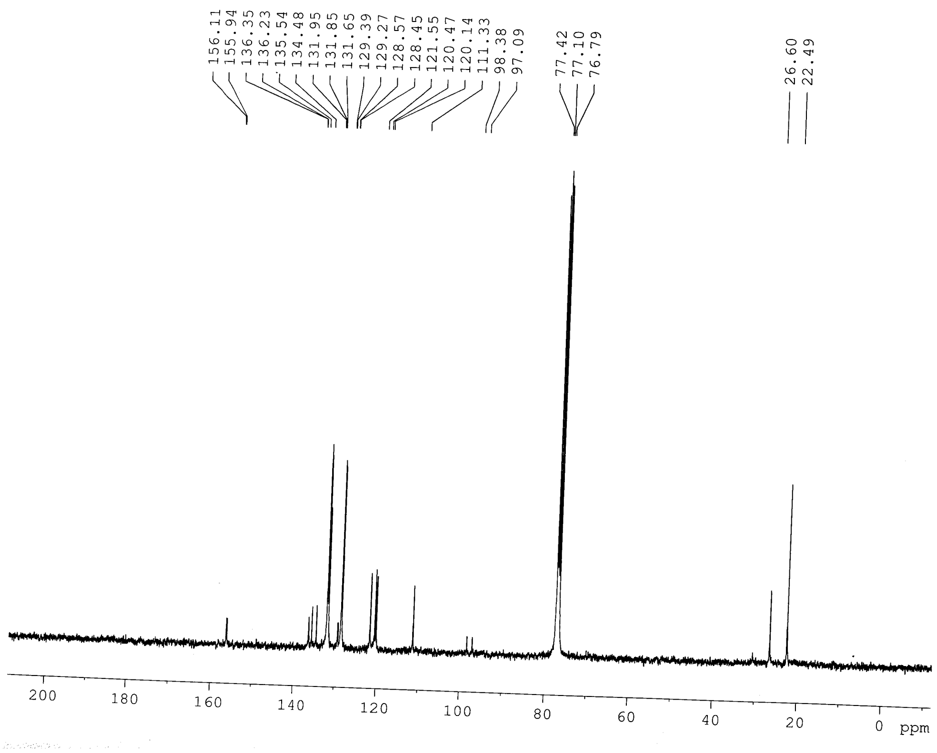

Figure S7: ^{13}C NMR spectrum of compound **6**.

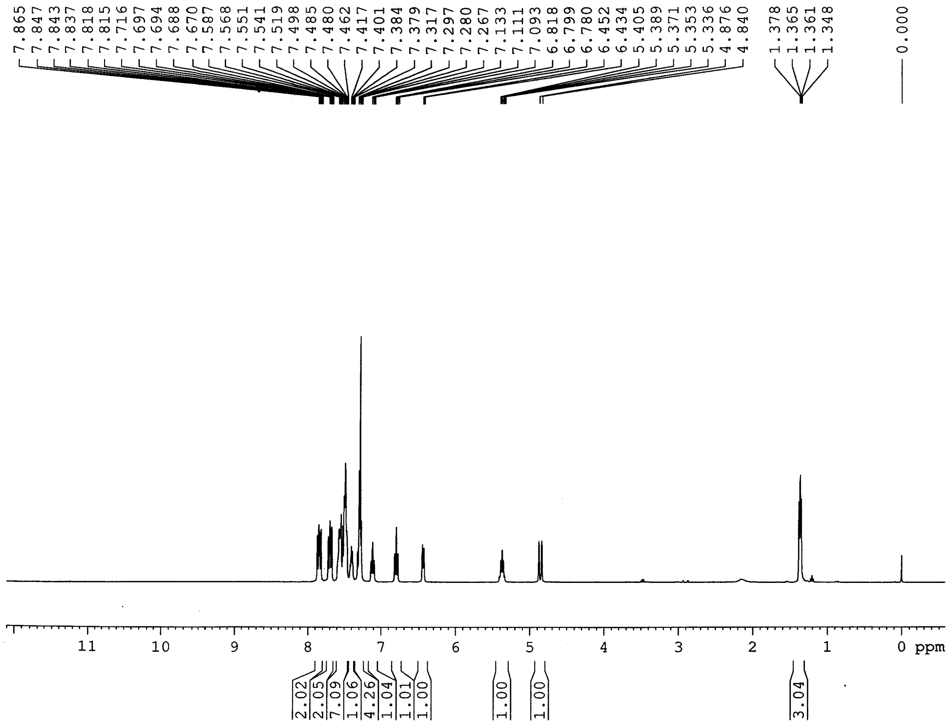

Figure S73: ^{1}H NMR spectrum of compound **7**.

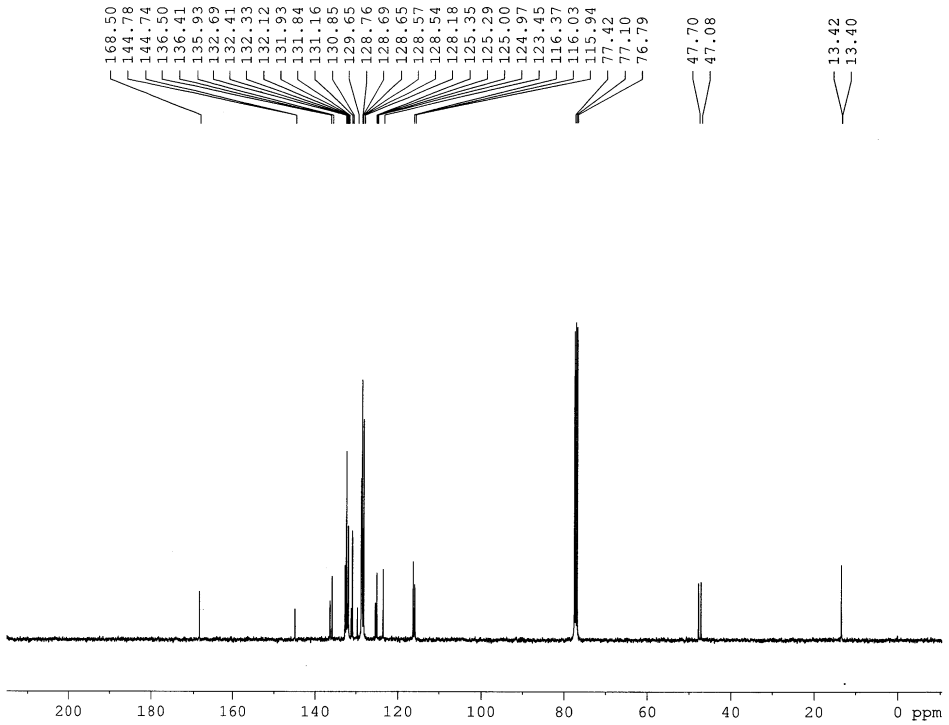

Figure S74: ^{13}C NMR spectrum of compound **7**.

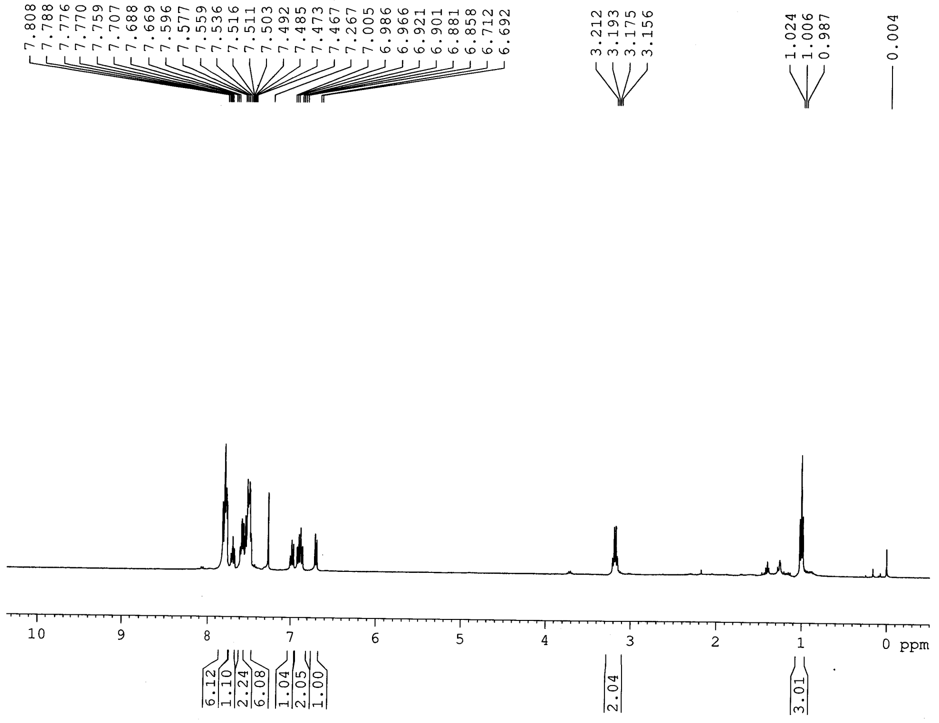

Figure S75: ^{1}H NMR spectrum of compound **8**.

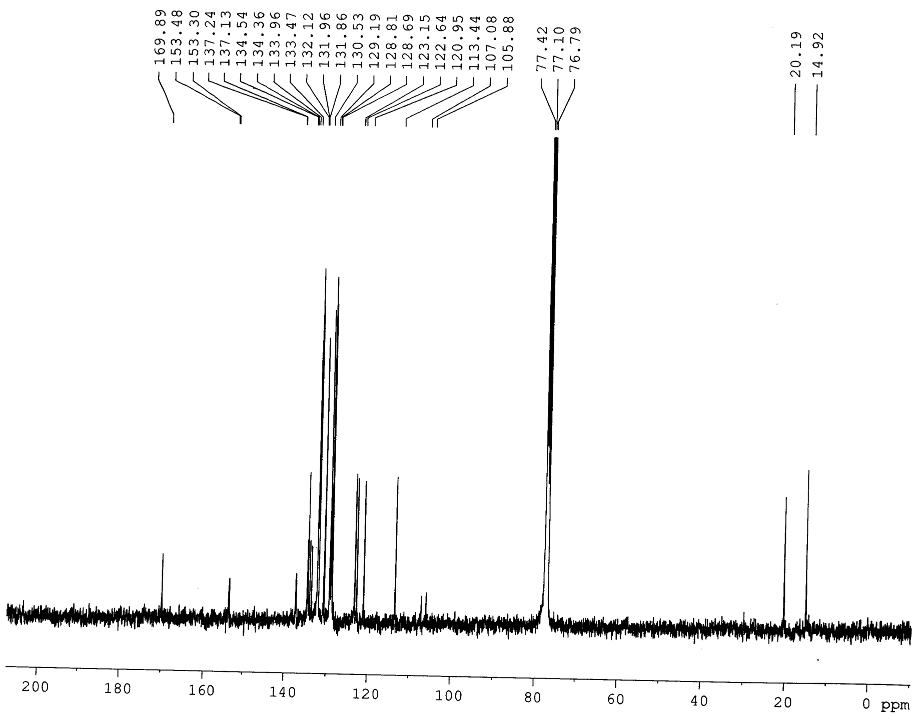

Figure S76: ^{13}C NMR spectrum of compound **8**.

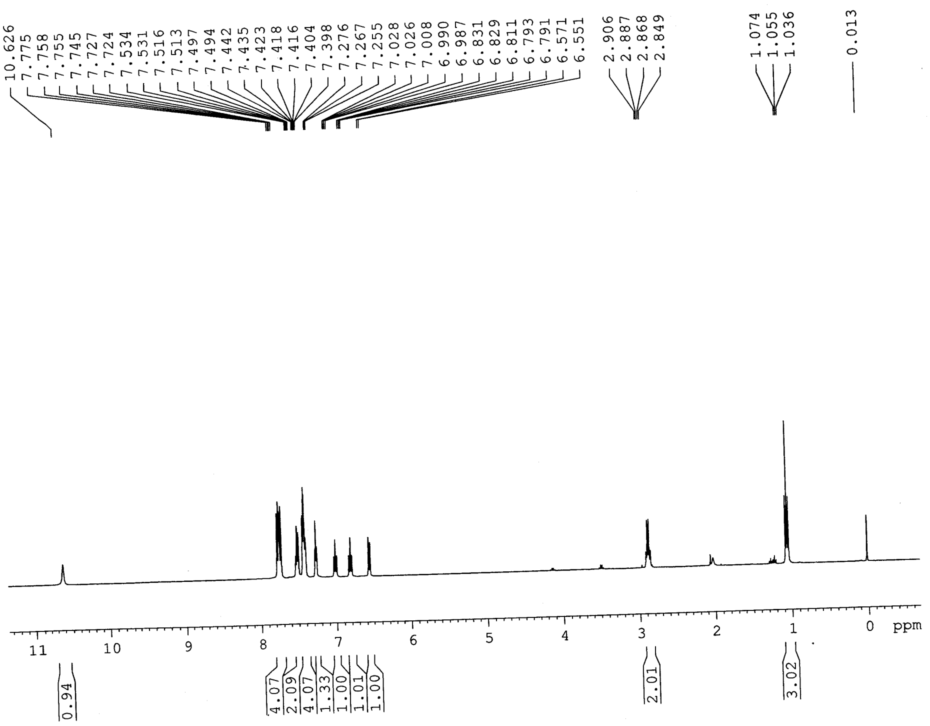

Figure S77: ^{1}H NMR spectrum of compound **9**.

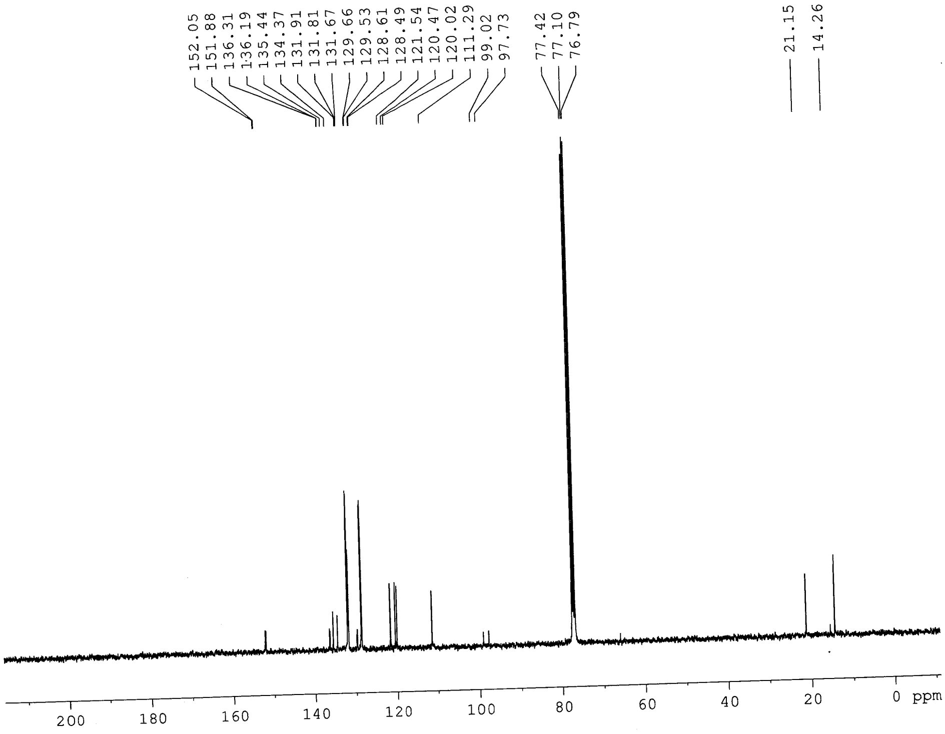

Figure S78: ^{13}C NMR spectrum of compound **9**.

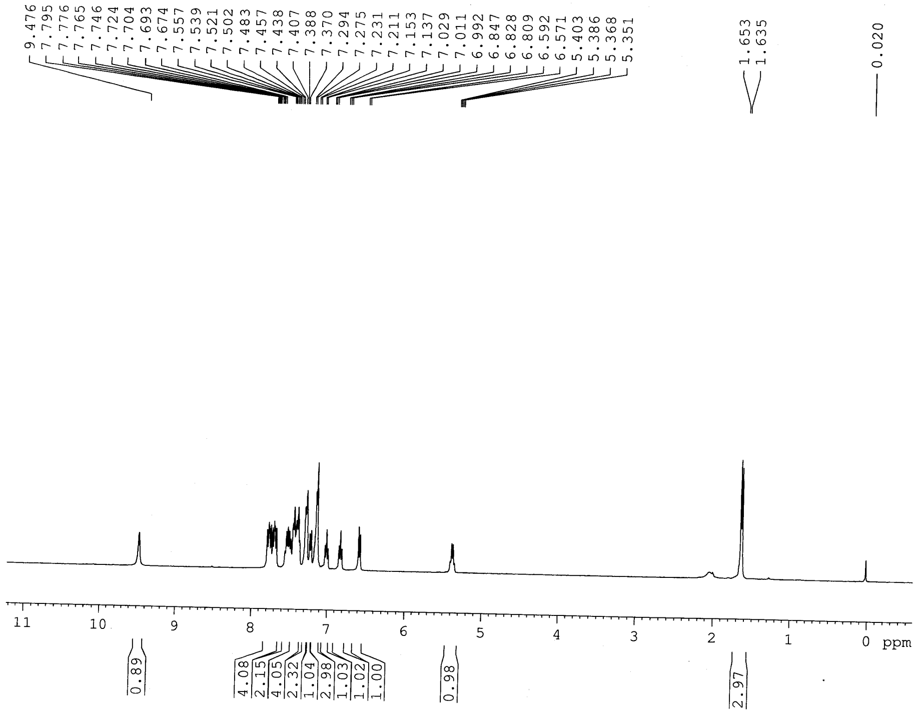

Figure S79: ^{1}H NMR spectrum of compound **10**.

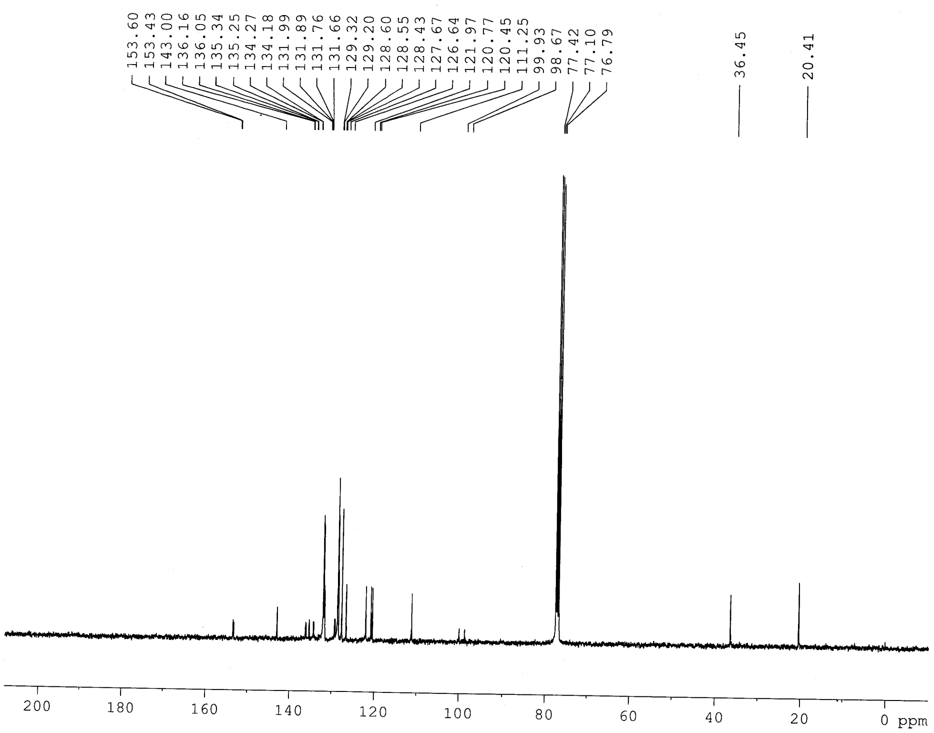

Figure S80: ^{13}C NMR spectrum of compound **10**.

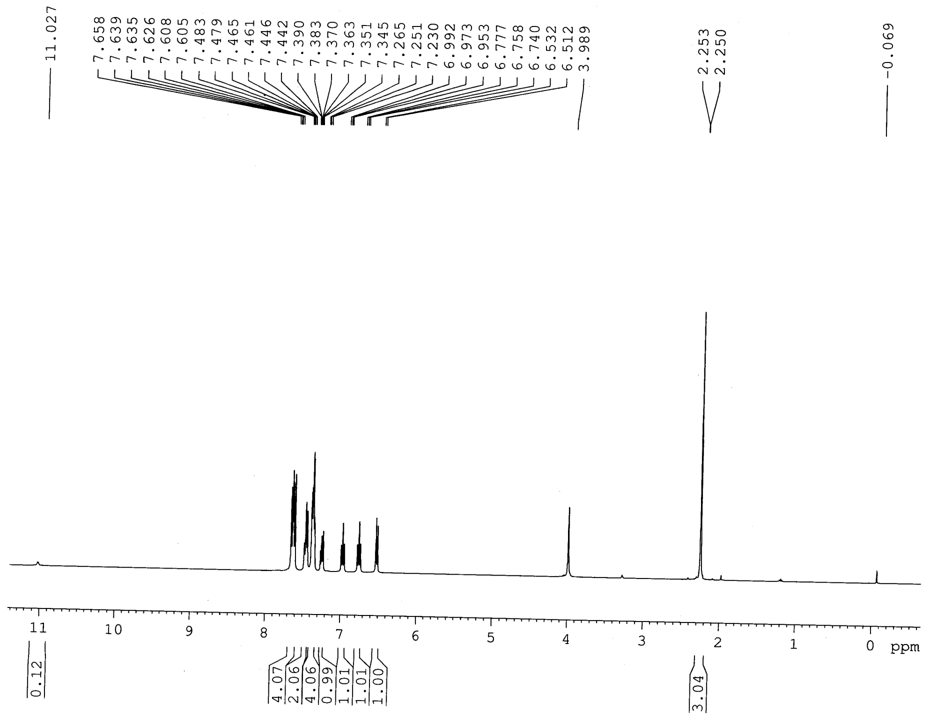

Figure S81: ^{1}H NMR spectrum of compound **11**.

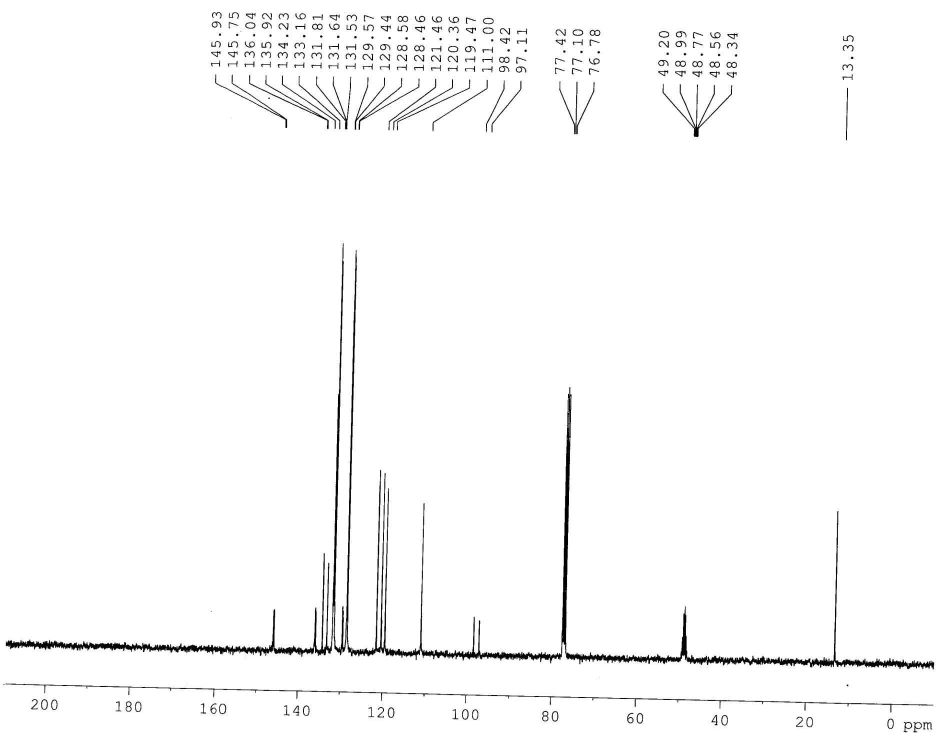

Figure S82: ^{13}C NMR spectrum of compound **11**.

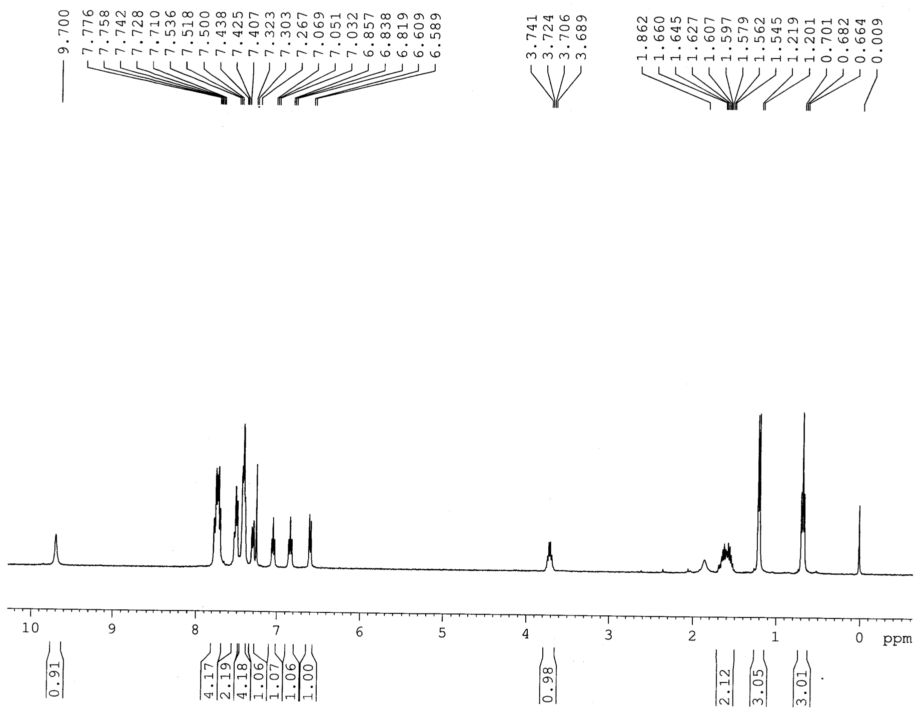

Figure S83: ^{1}H NMR spectrum of compound **12**.

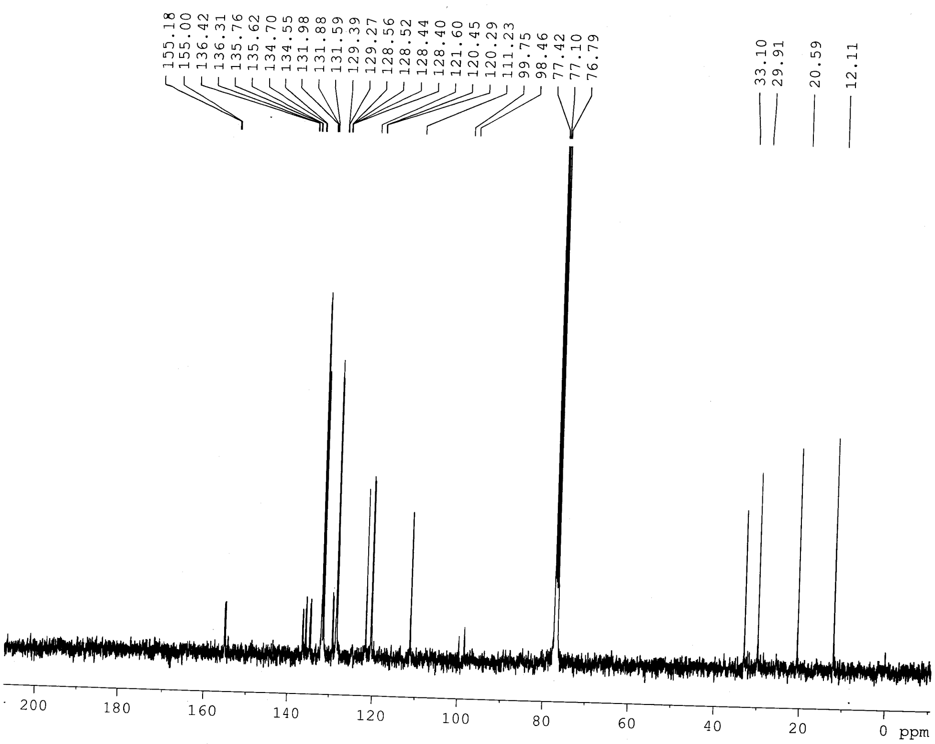

Figure S84: ^{13}C NMR spectrum of compound **12**.

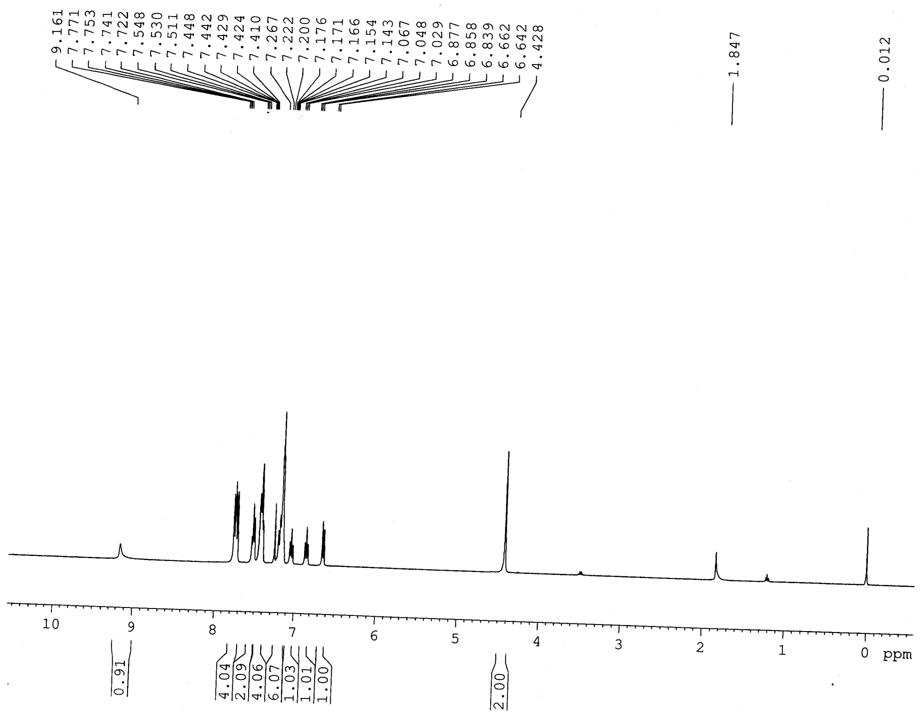

Figure S85: ^{1}H NMR spectrum of compound **13**.

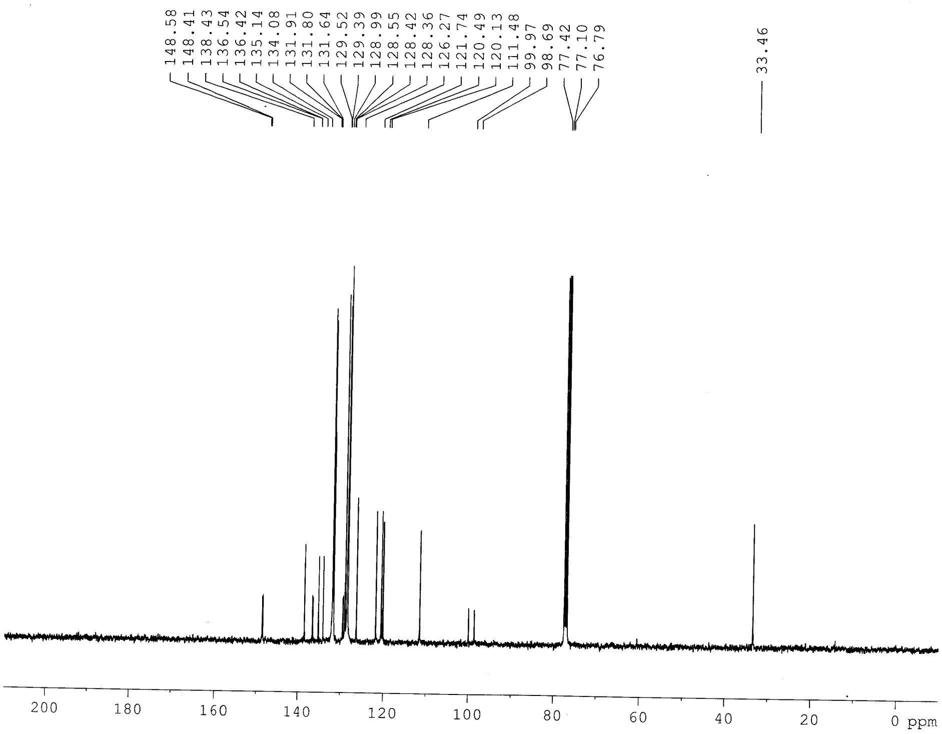

Figure S86: ^{13}C NMR spectrum of compound **13**.

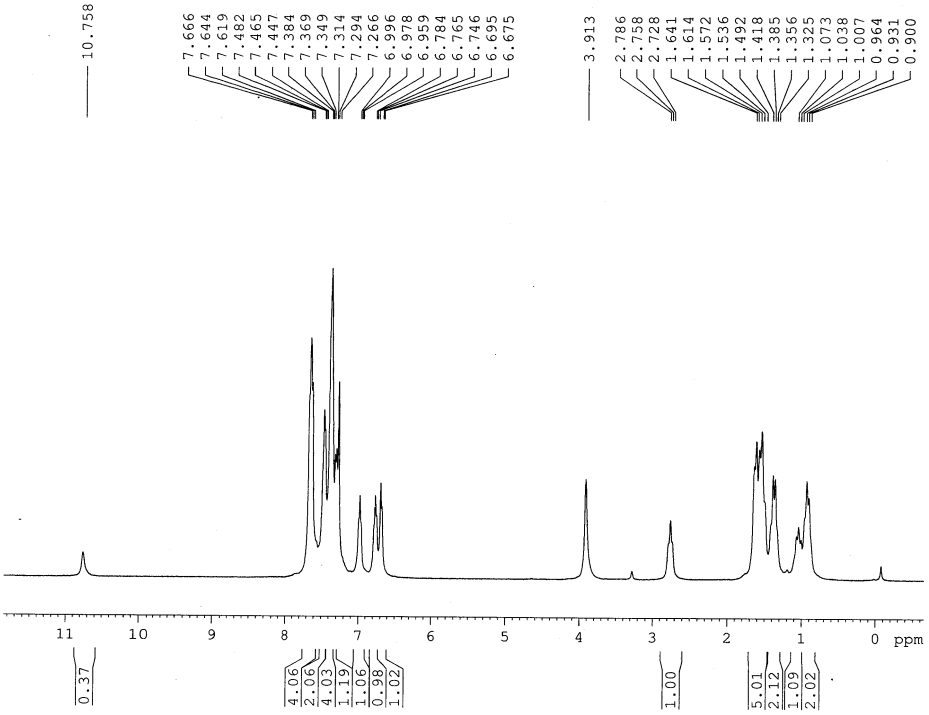

Figure S87: ^{1}H NMR spectrum of compound **14**.

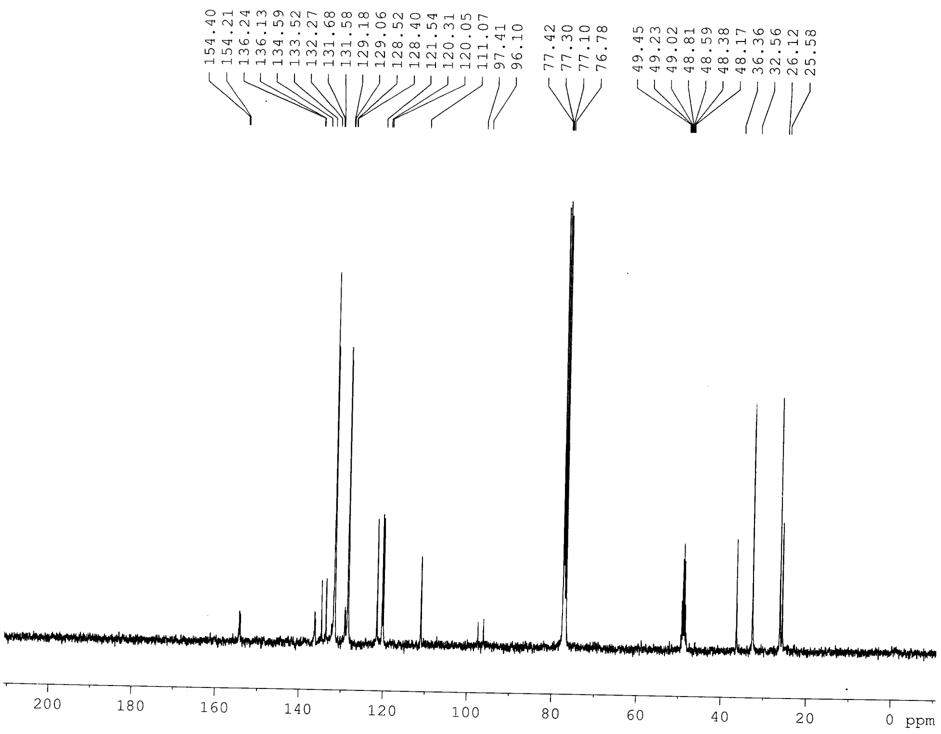

Figure S88: ^{13}C NMR spectrum of compound **14**.

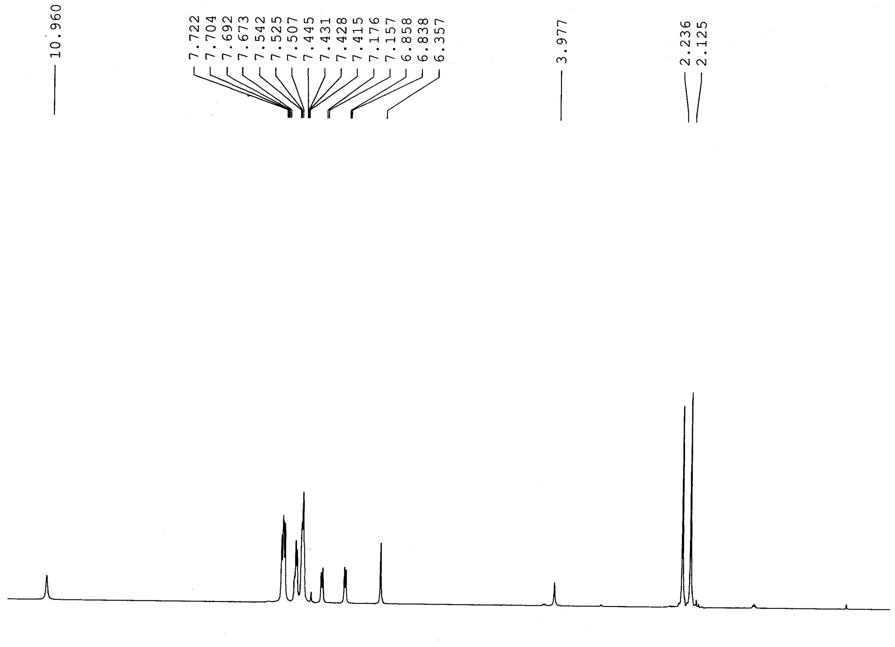

Figure S89: ^{1}H NMR spectrum of compound **15**.

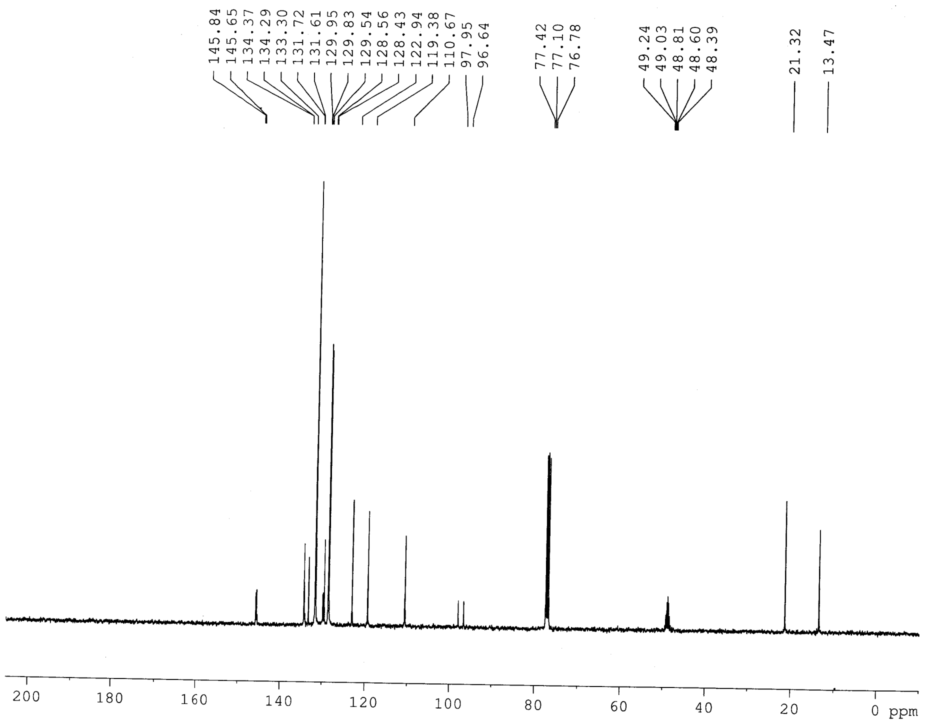

Figure S90: ^{13}C NMR spectrum of compound **15**.

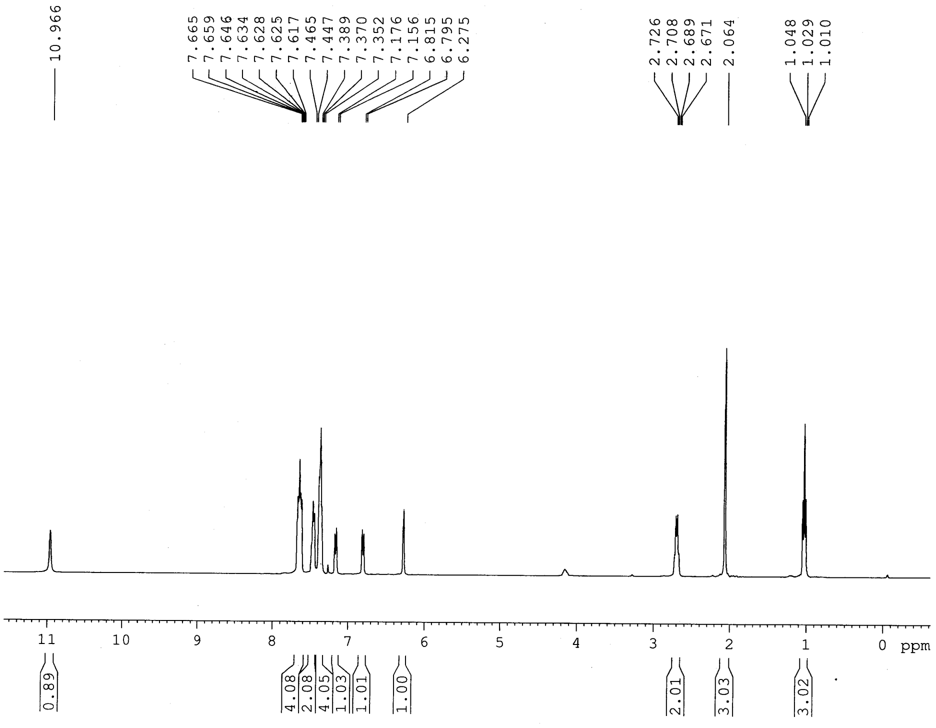

Figure S91: ^{1}H NMR spectrum of compound **16**.

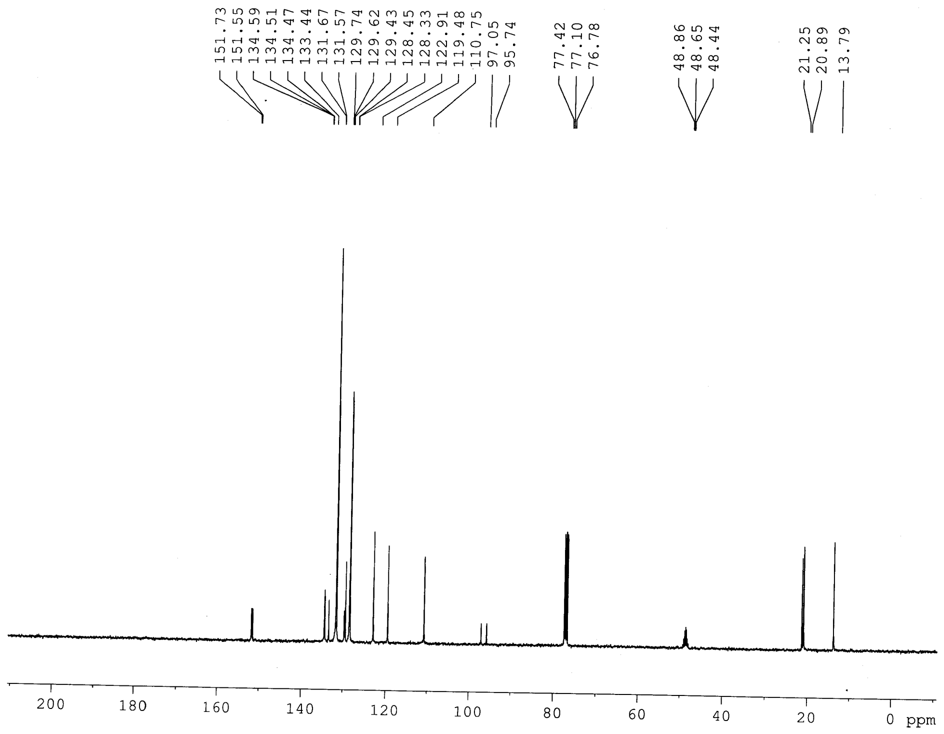

Figure S92: ^{13}C NMR spectrum of compound **16**.

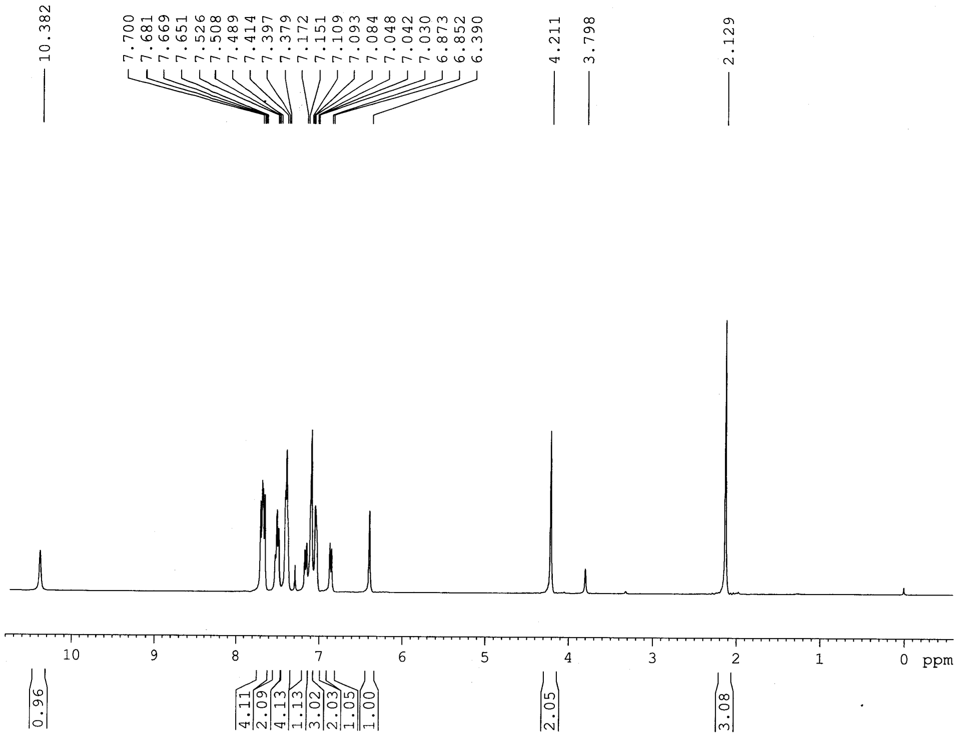

Figure S93: ^{1}H NMR spectrum of compound **17.**

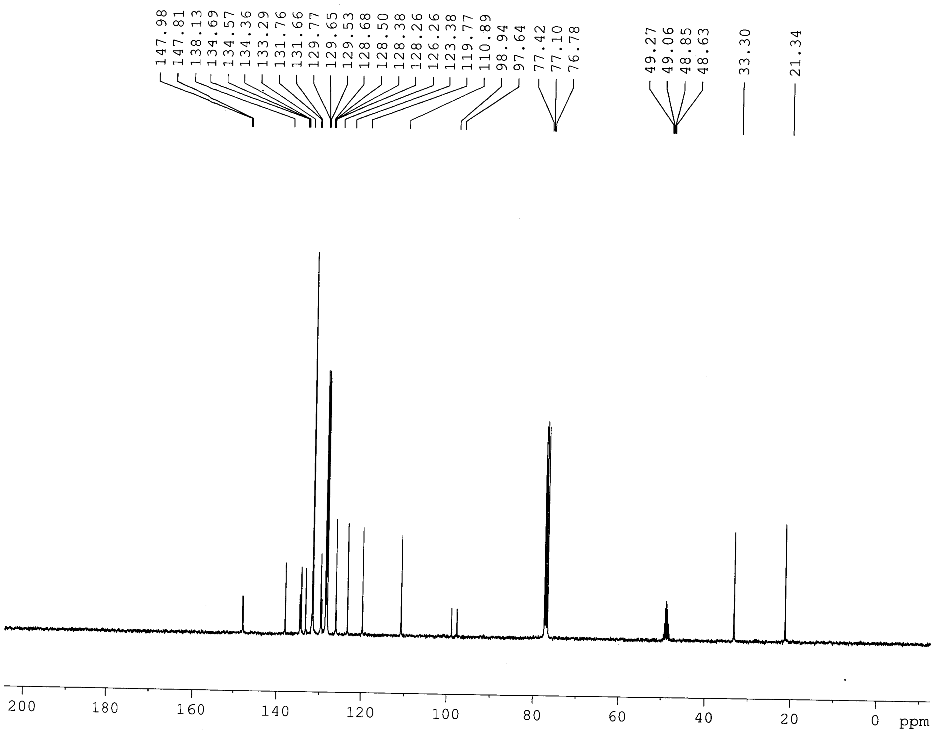

Figure S94: ^{13}C NMR spectrum of compound **17**.

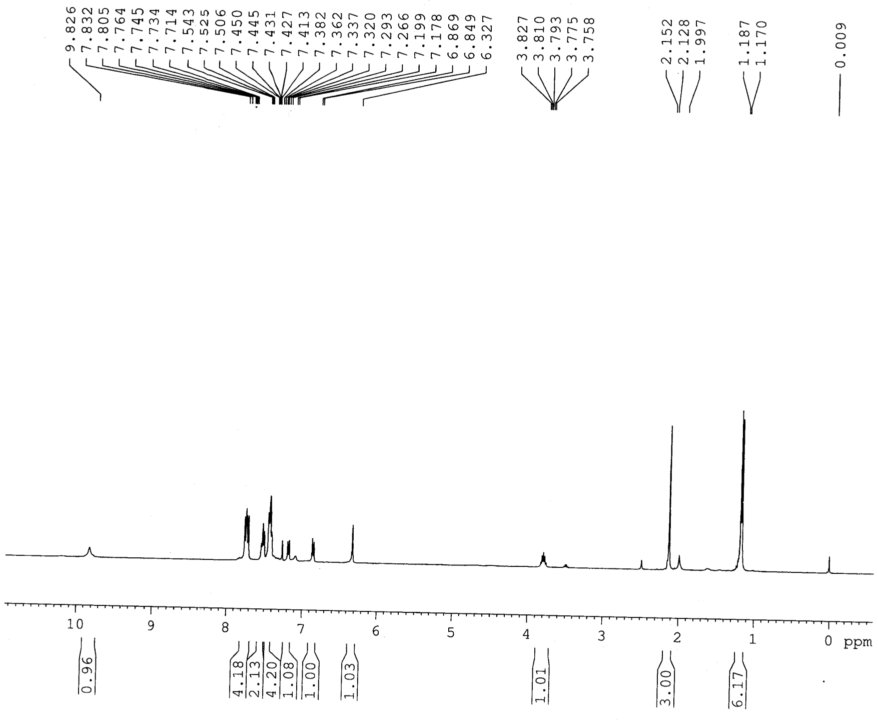

Figure S95: ^{1}H NMR spectrum of compound **18**.

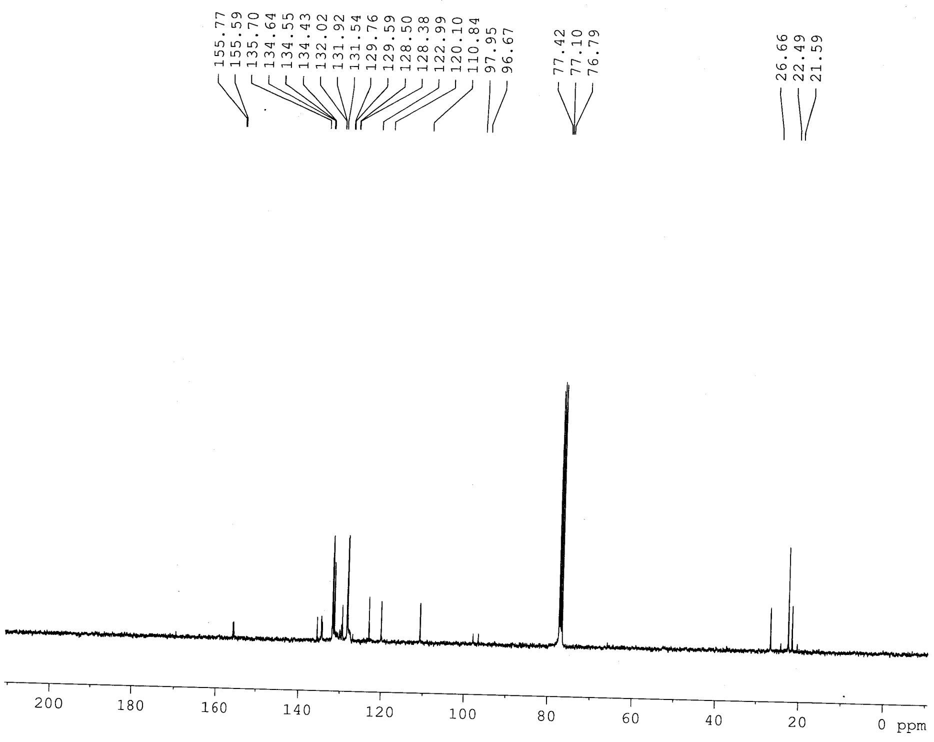

Figure S96: ^{13}C NMR spectrum of compound **18**.

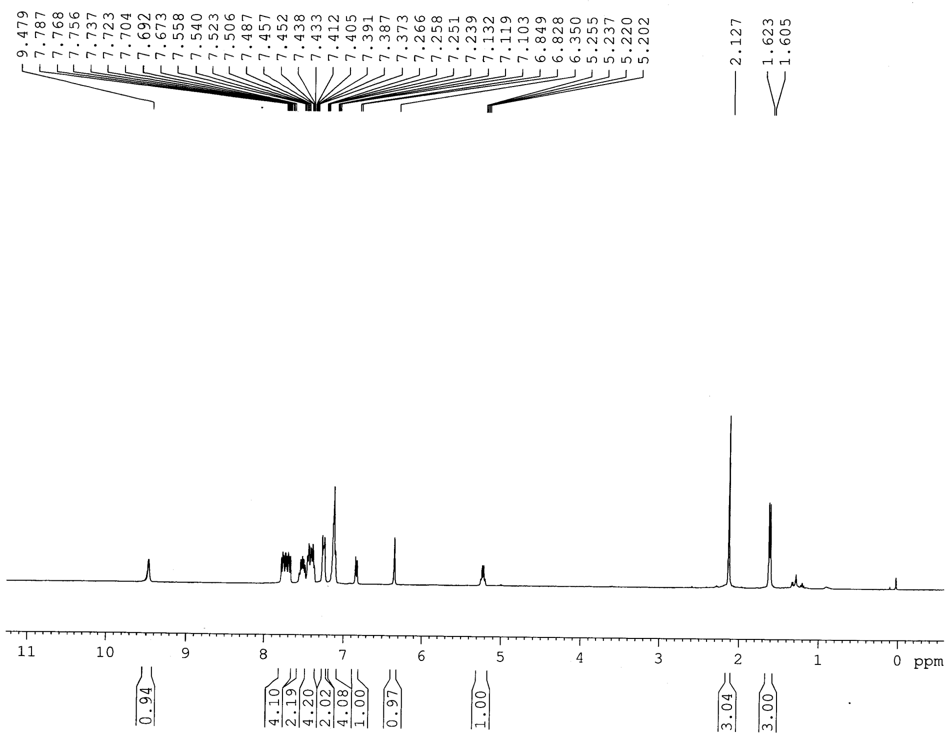

Figure S97: ^{1}H NMR spectrum of compound **19**.

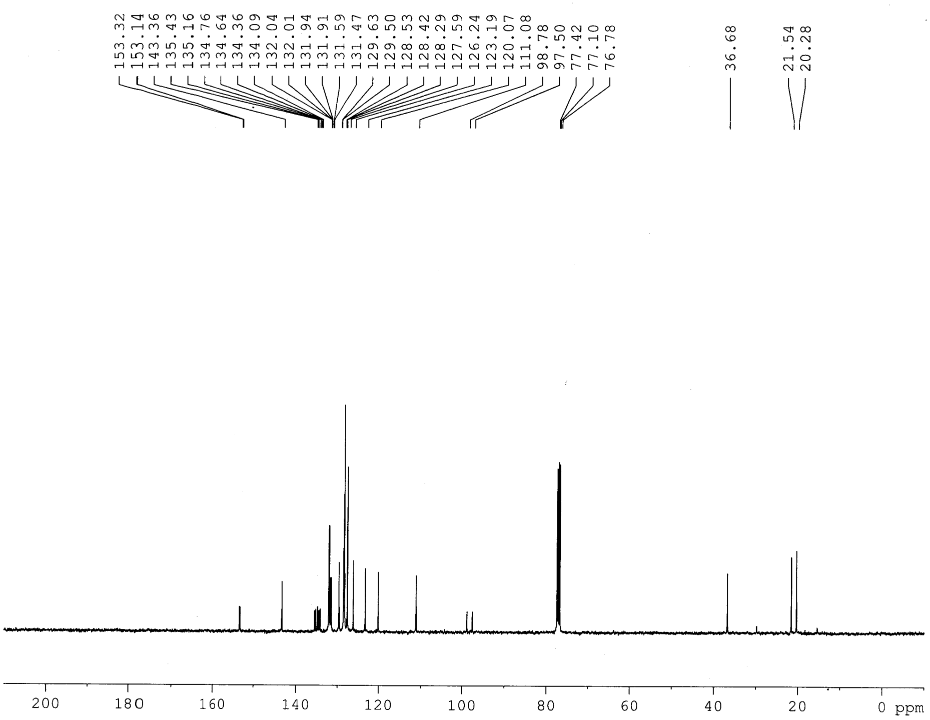

Figure S98: ^{13}C NMR spectrum of compound **19**.

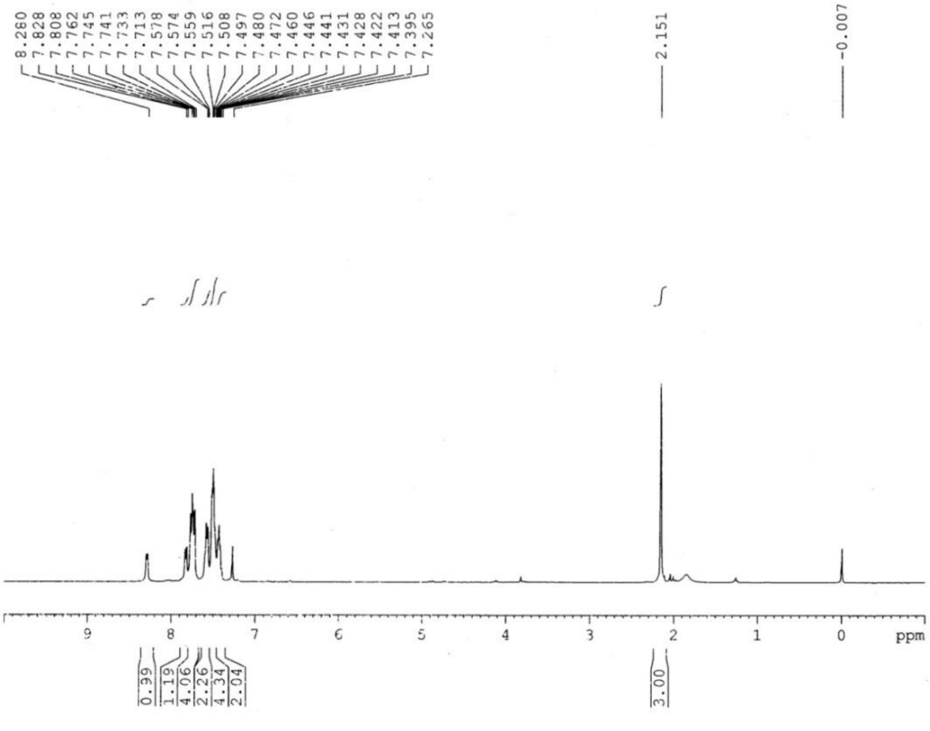

Figure S99: ^{1}H NMR spectrum of compound **20**.

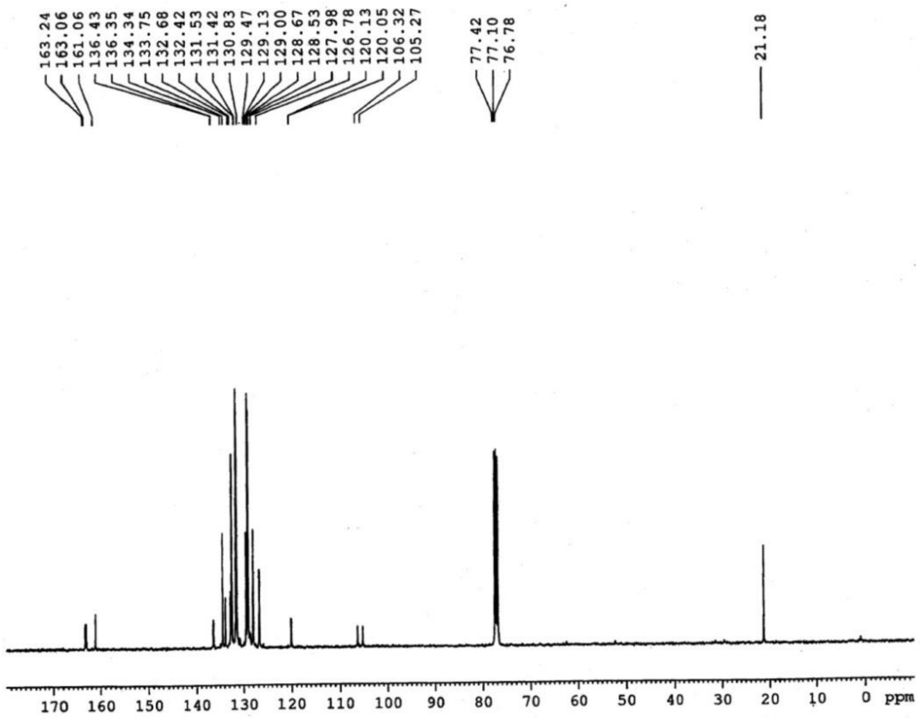

Figure S100: ^{13}C NMR spectrum of compound **20**.

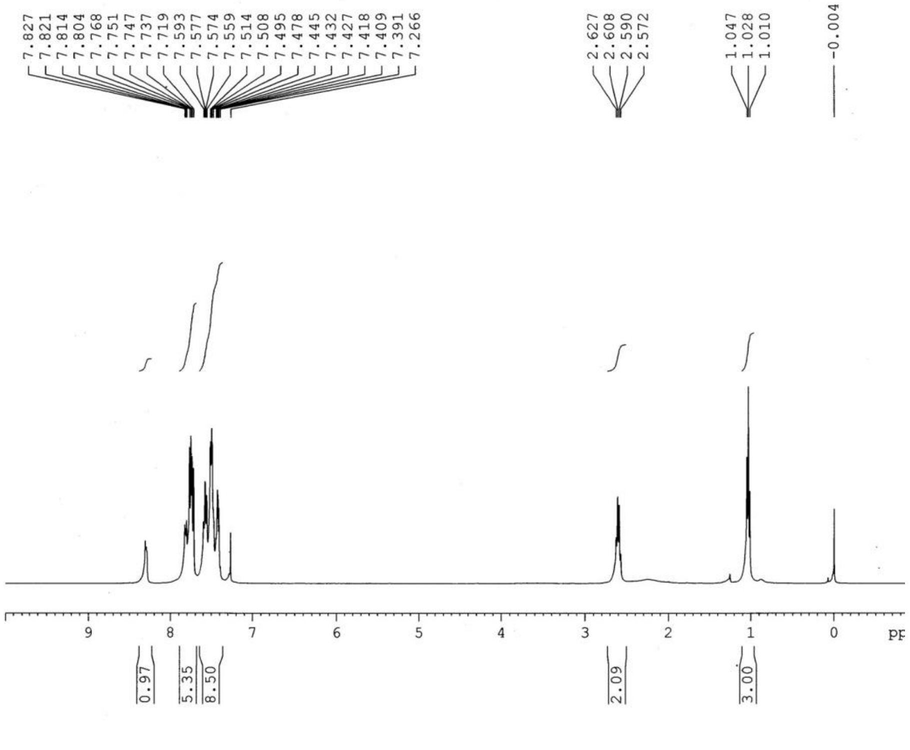

Figure S101: ^{1}H NMR spectrum of compound **21**.

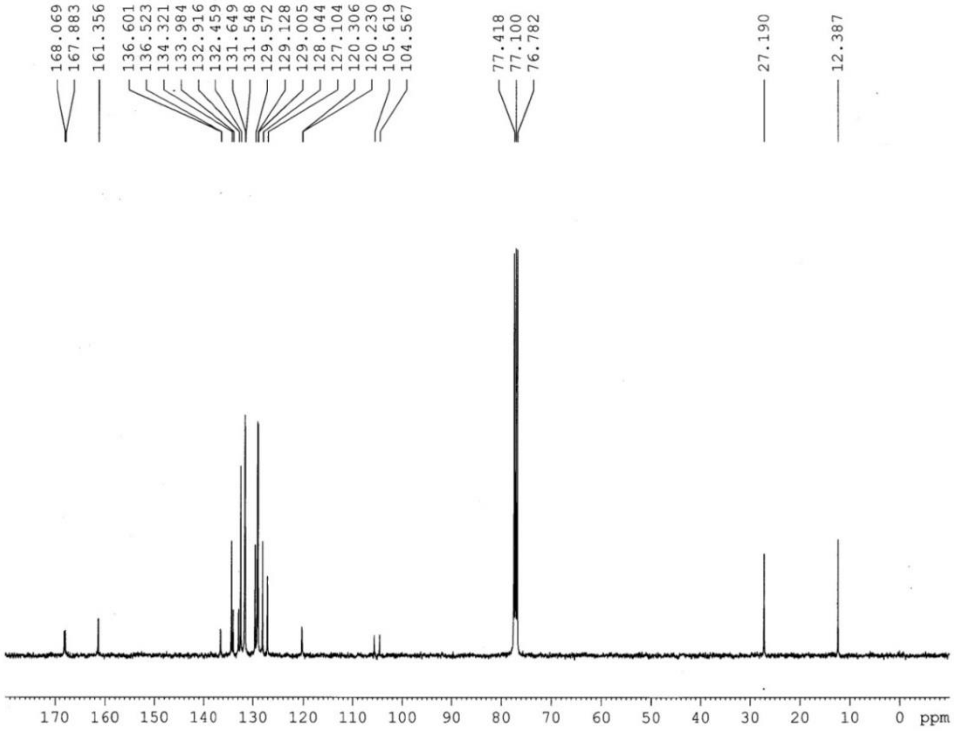

Figure S102: ^{13}C NMR spectrum of compound **21**.

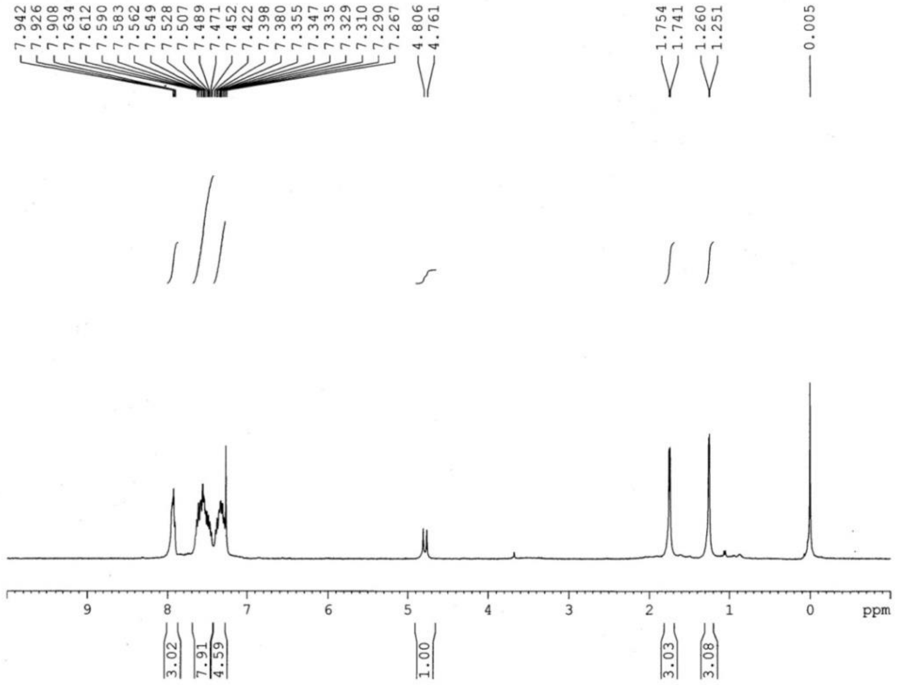

Figure S103: ^{1}H NMR spectrum of compound **22**.

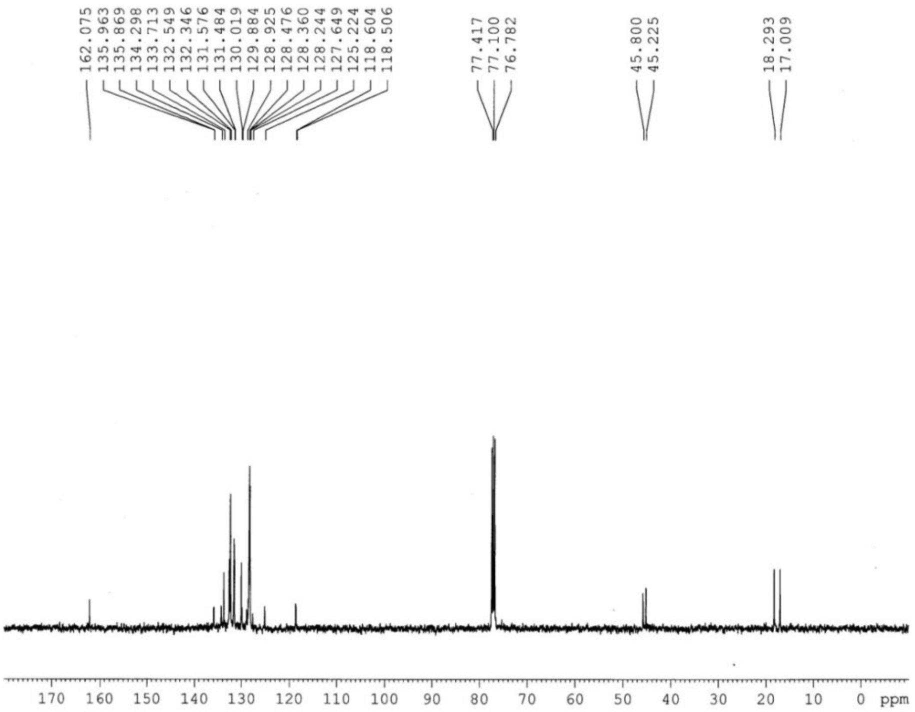

Figure S104: ^{13}C NMR spectrum of compound **22**.

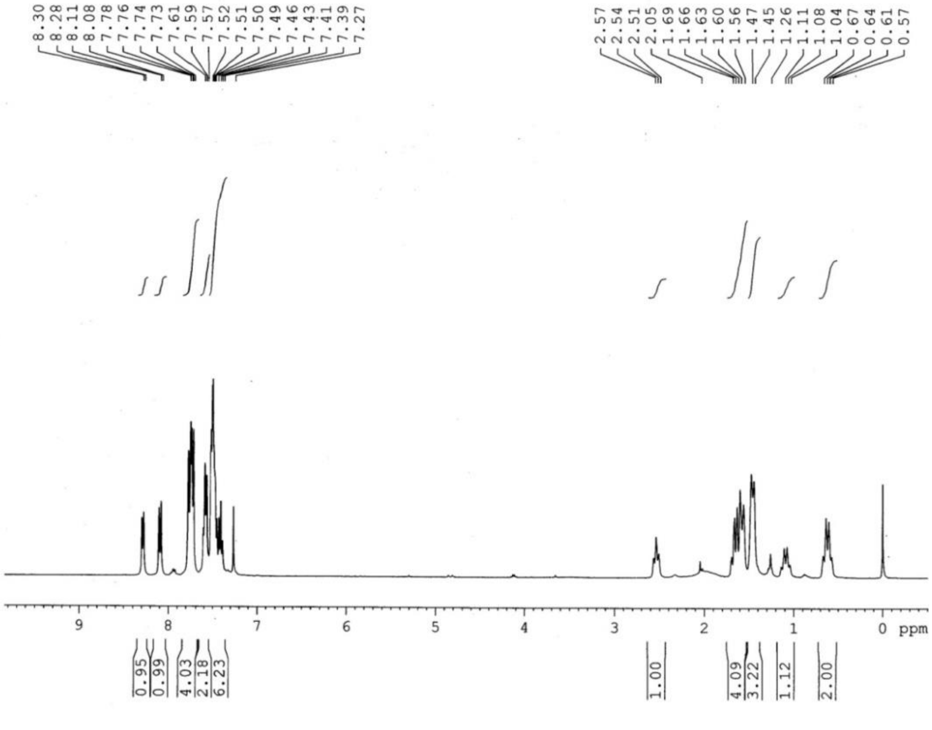

Figure S105: ^{1}H NMR spectrum of compound **23**.

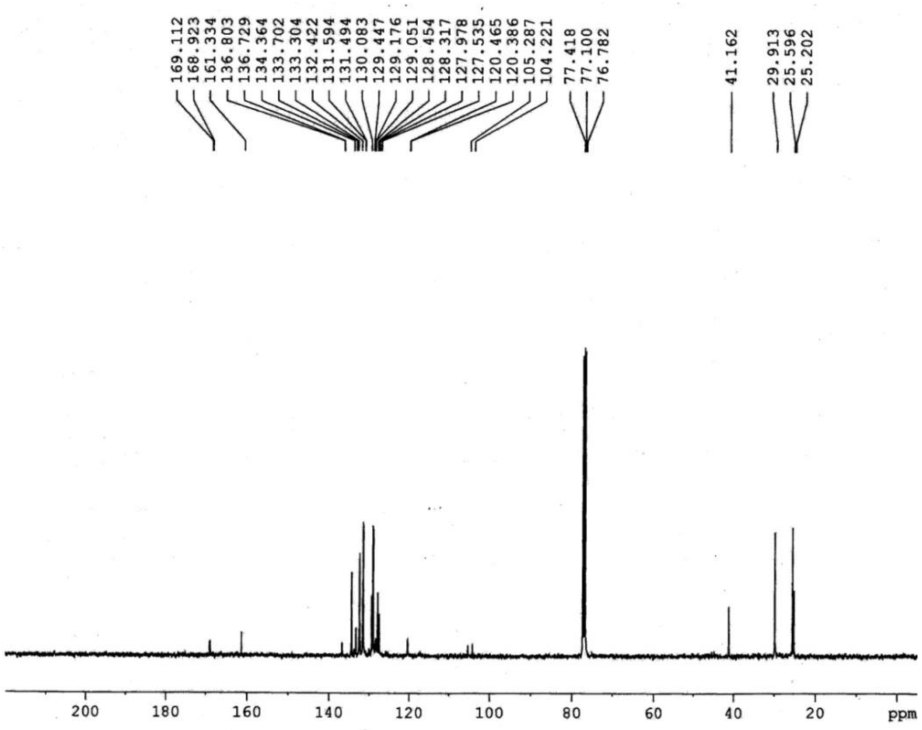

Figure S106: ^{13}C NMR spectrum of compound **23**.

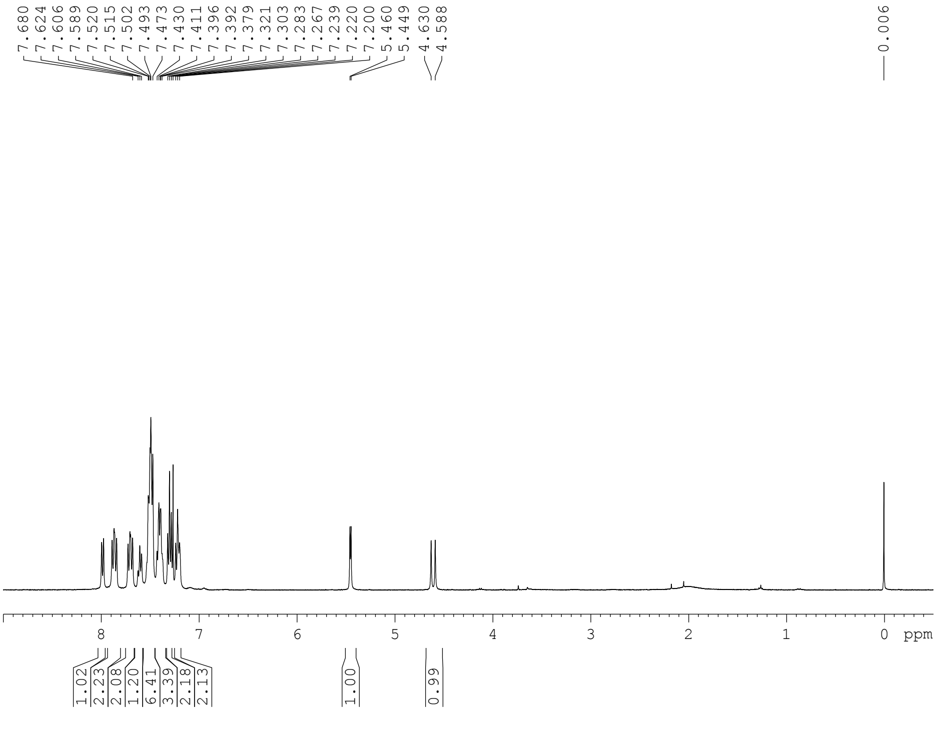

Figure S107: ^{1}H NMR spectrum of compound **24**.

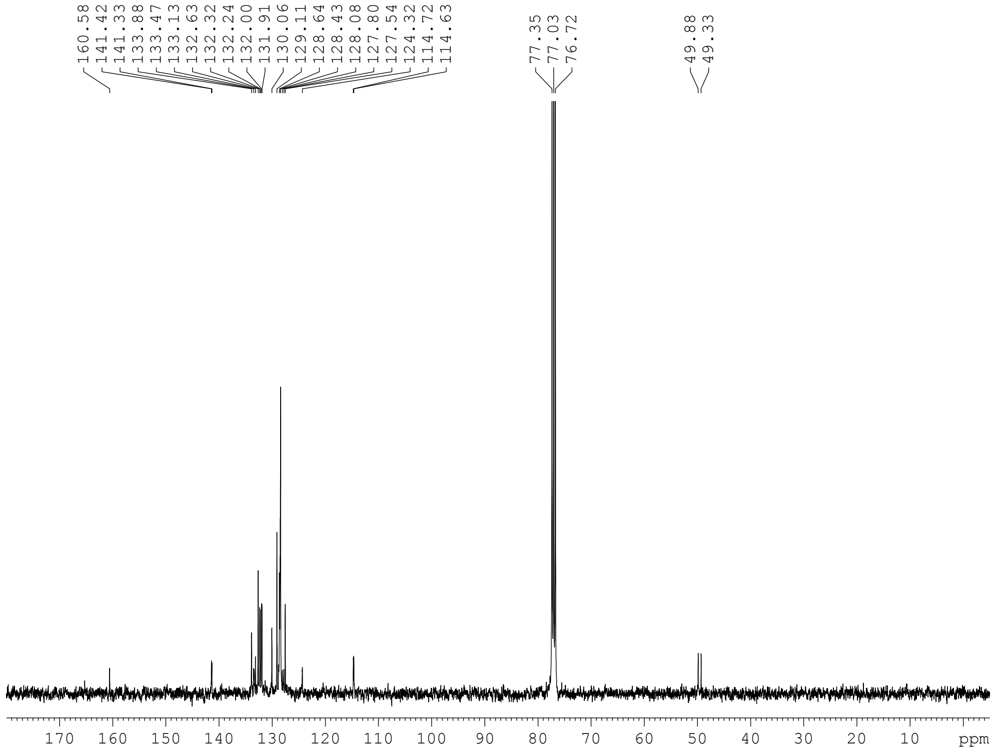

Figure S108: ^{13}C NMR spectrum of compound **24**.

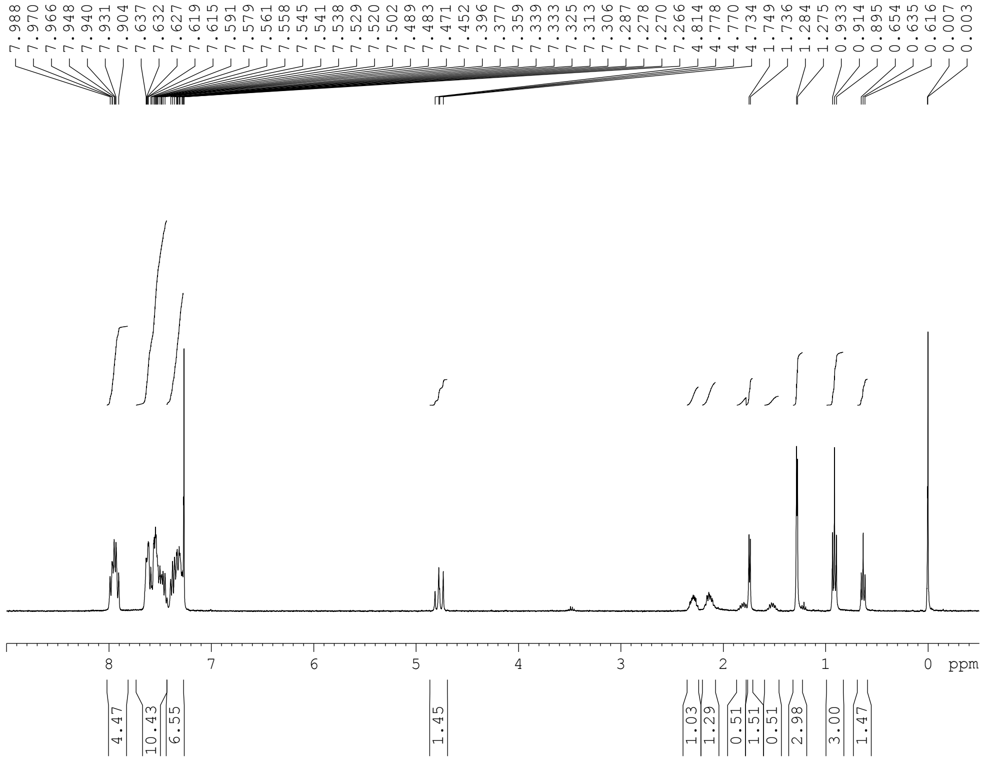

Figure S109: ^{1}H NMR spectrum of compound **251**.

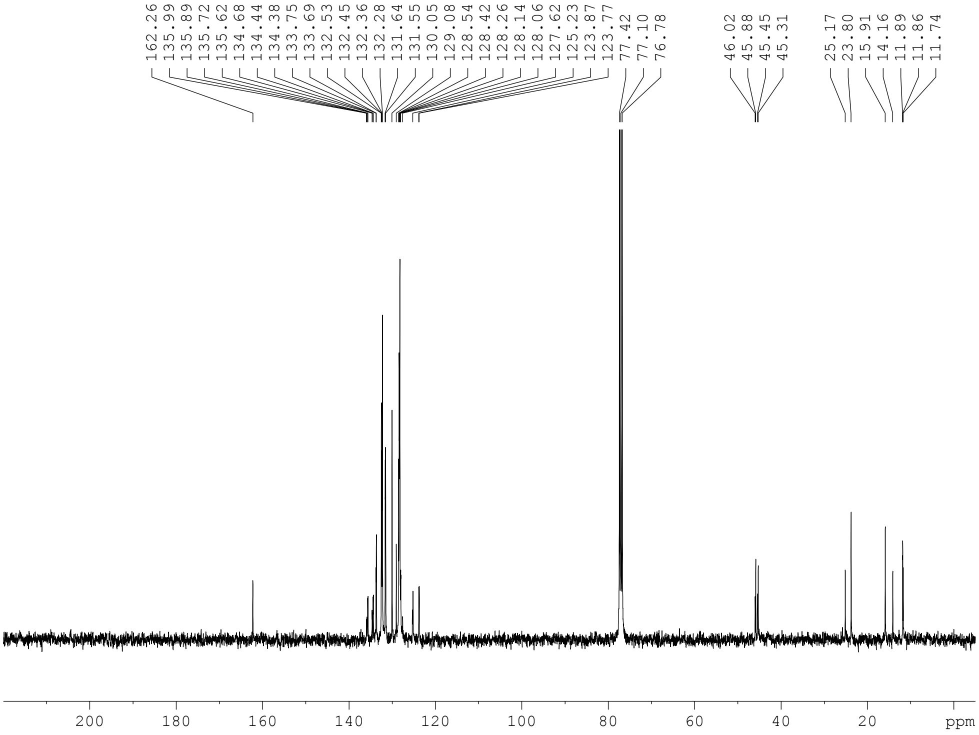

Figure S110: ^{13}C NMR spectrum of compound **25**.

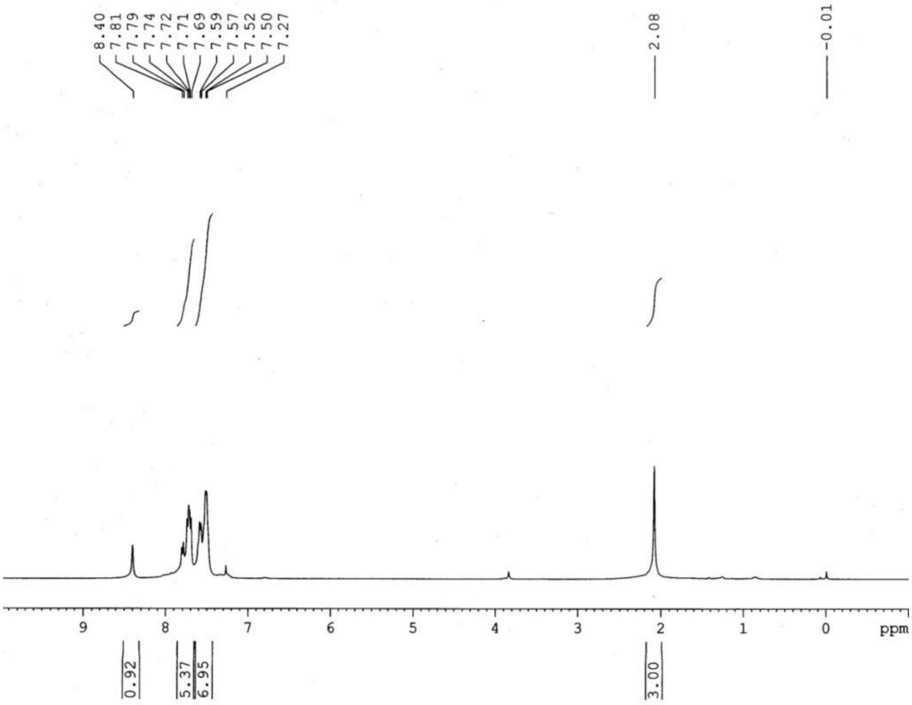

Figure S111: ^{1}H NMR spectrum of compound **26**.

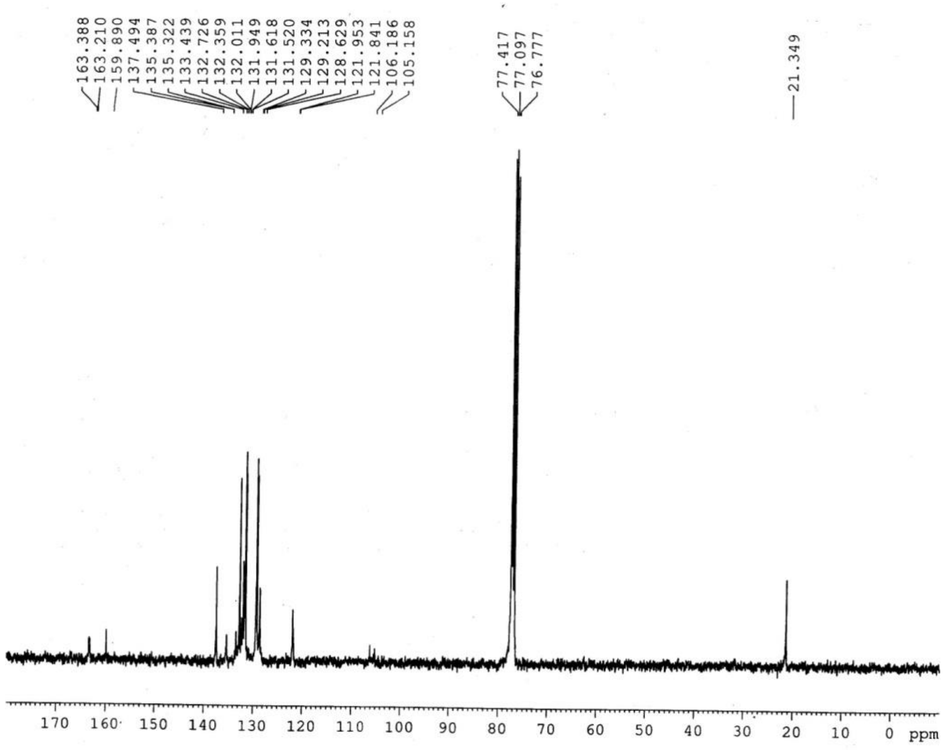

Figure S112: ^{13}C NMR spectrum of compound **26**.

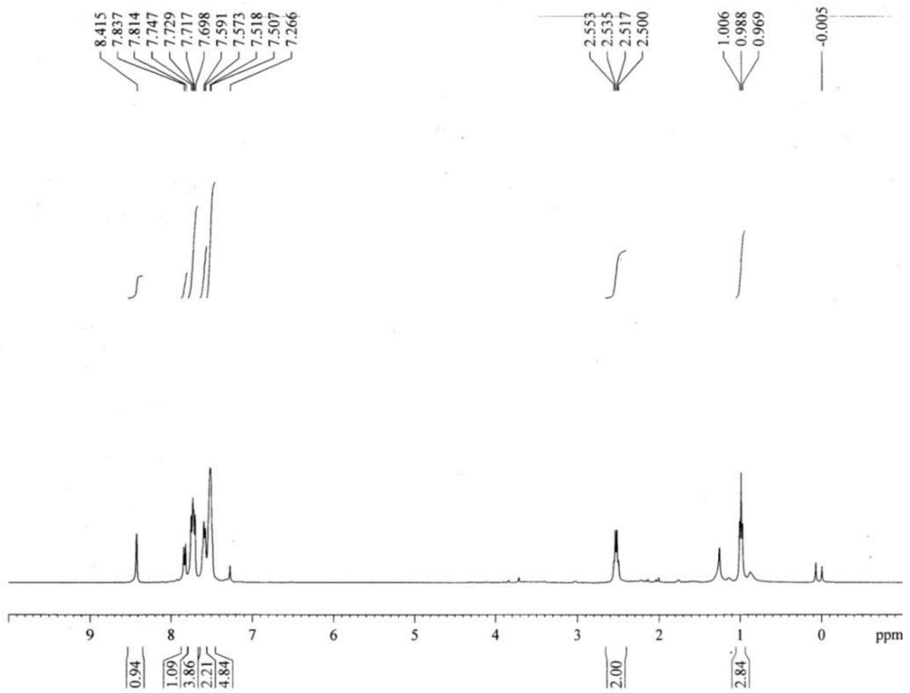

Figure S113: ^{1}H NMR spectrum of compound **27**.

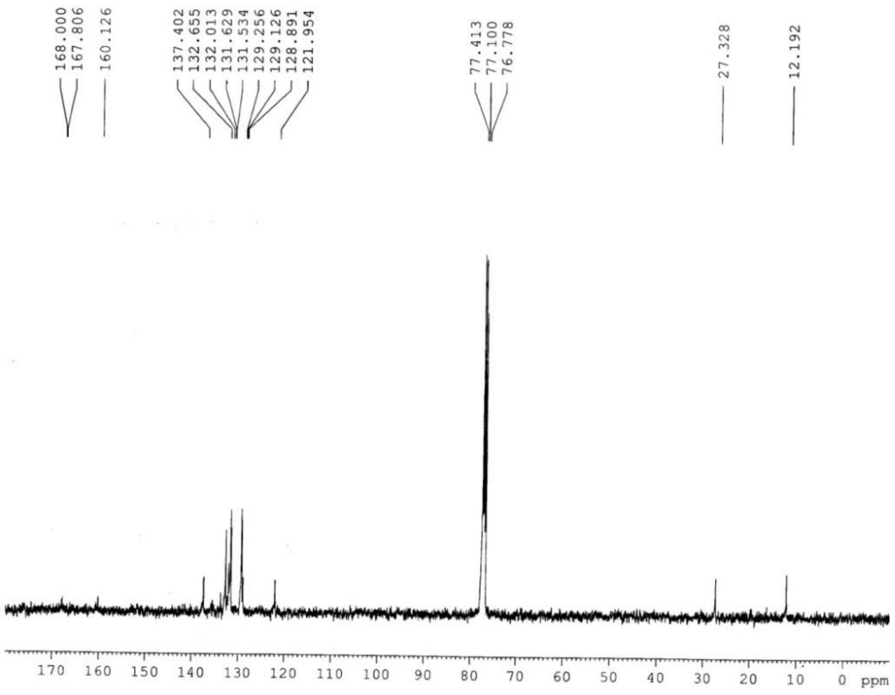

Figure S114: ^{13}C NMR spectrum of compound **27**.

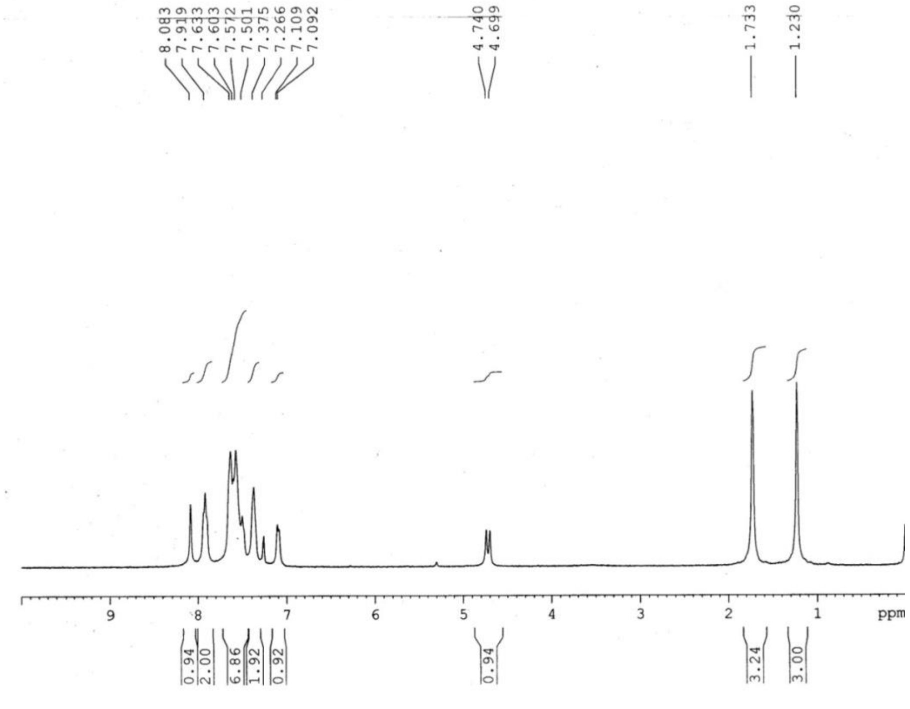

Figure S115: ^{1}H NMR spectrum of compound **28**.

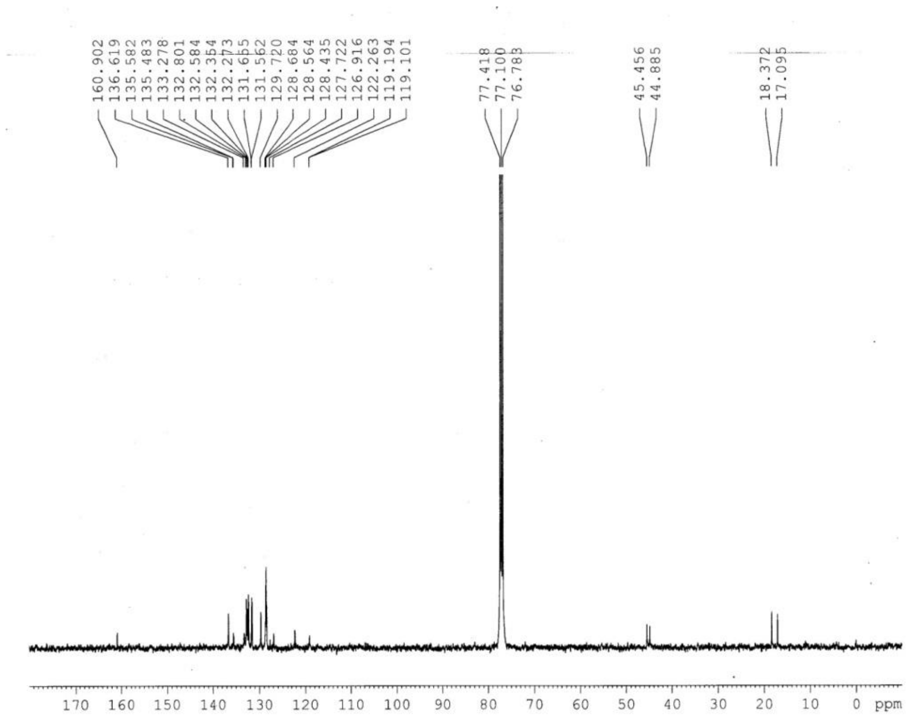

Figure S116: ^{13}C NMR spectrum of compound **28**.

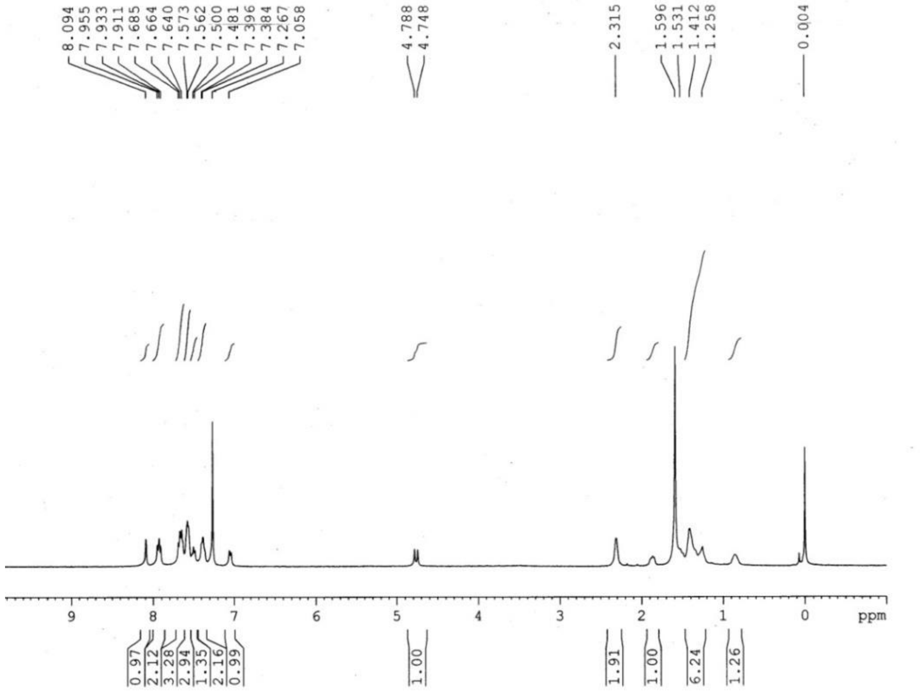

Figure S117: ^{1}H NMR spectrum of compound **29**.

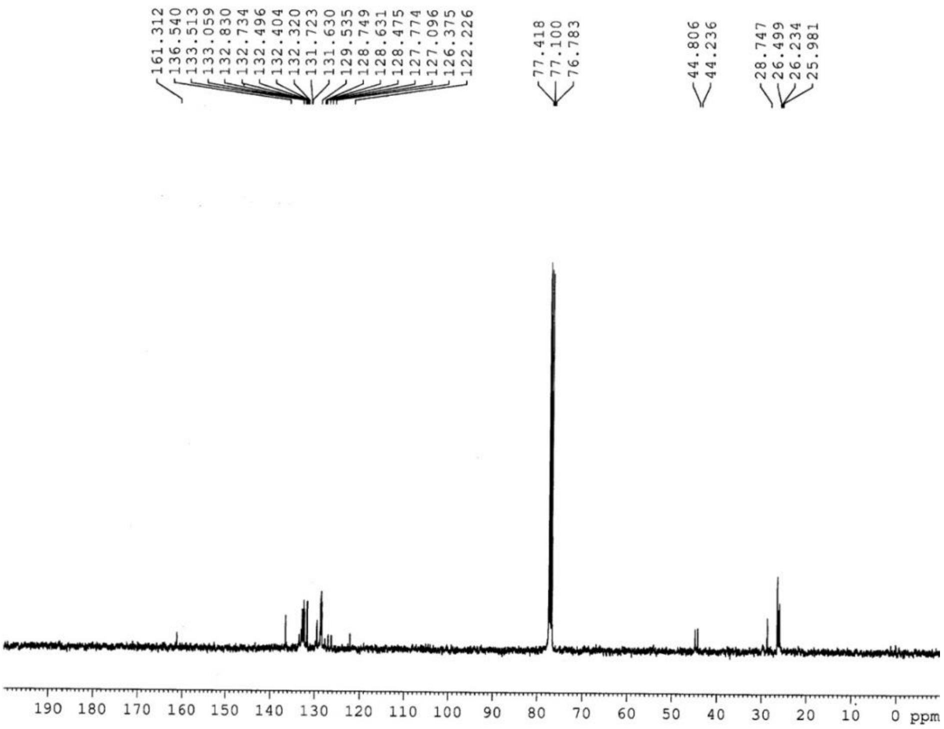

Figure S118: ^{13}C NMR spectrum of compound **29**.

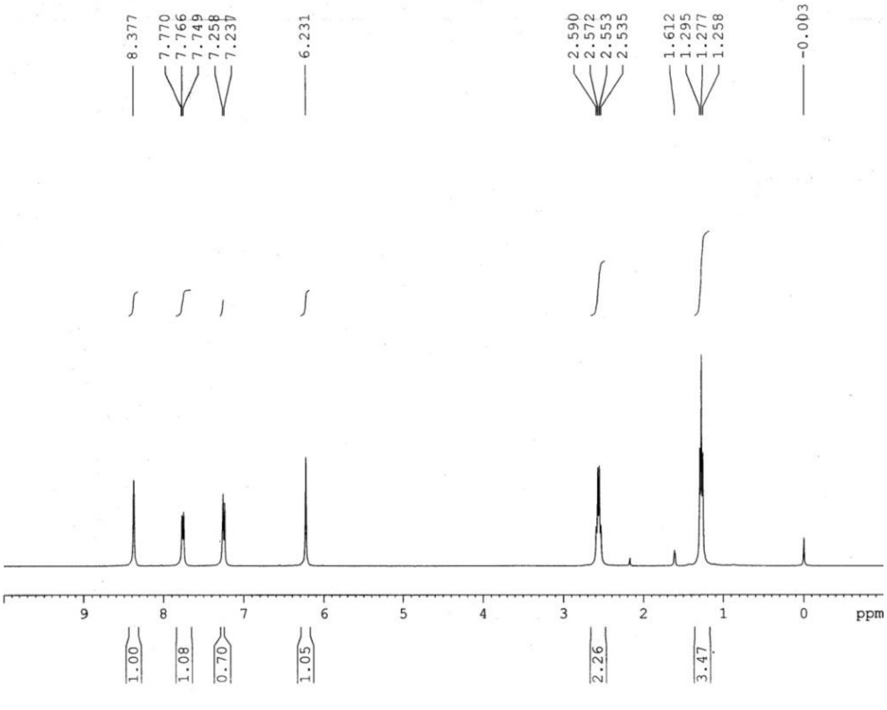

Figure S119: ^{1}H NMR spectrum of compound **33**.

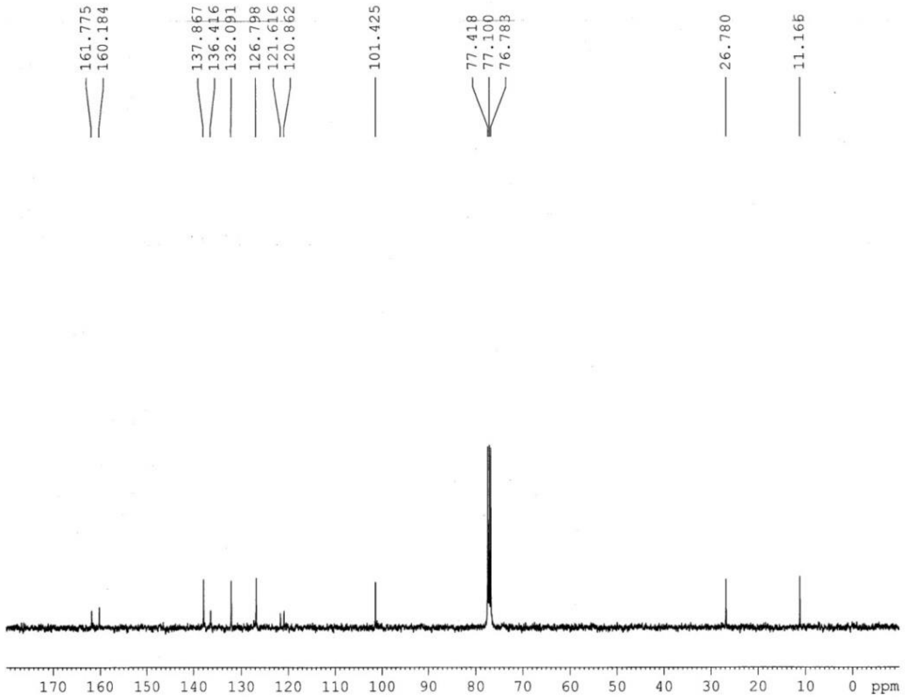

Figure S120: ^{13}C NMR spectrum of compound **33**.

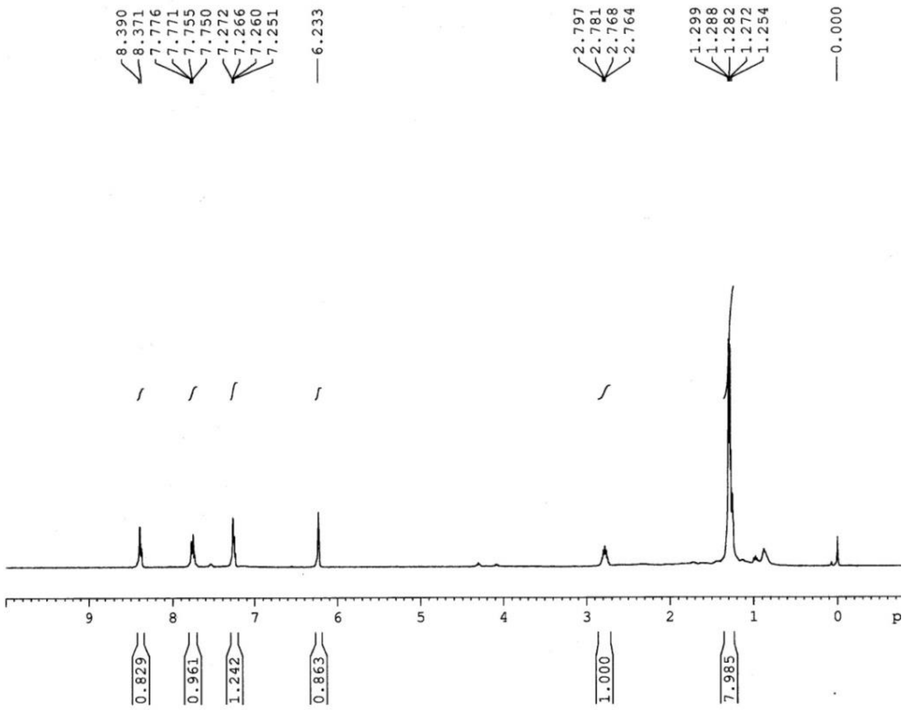

Figure S121: ^{1}H NMR spectrum of compound **34**.

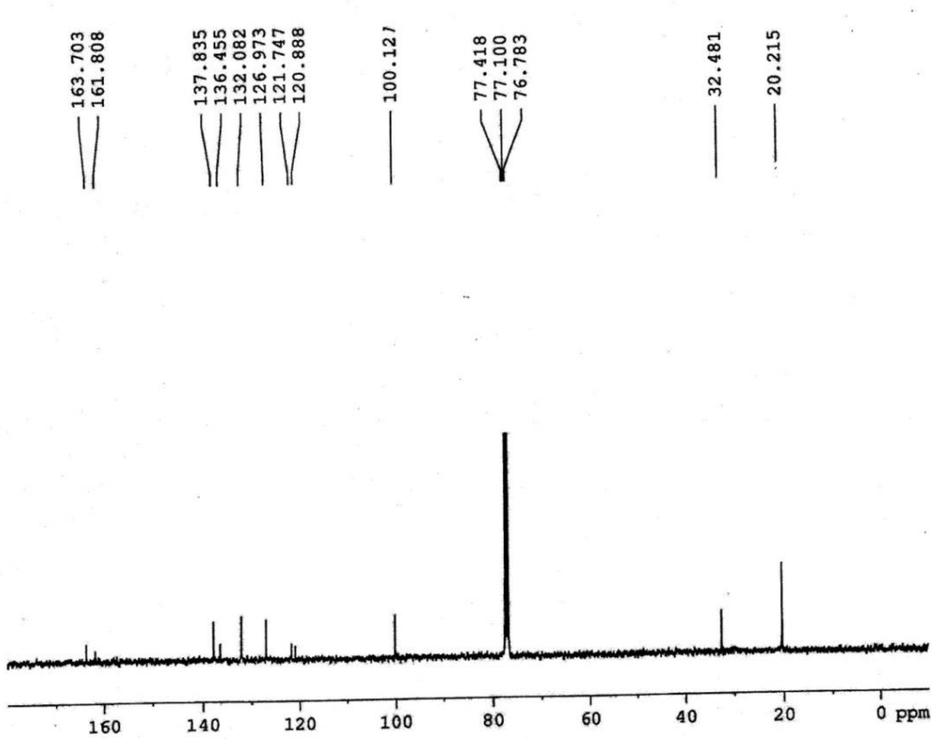

Figure S122: ^{13}C NMR spectrum of compound **34**.

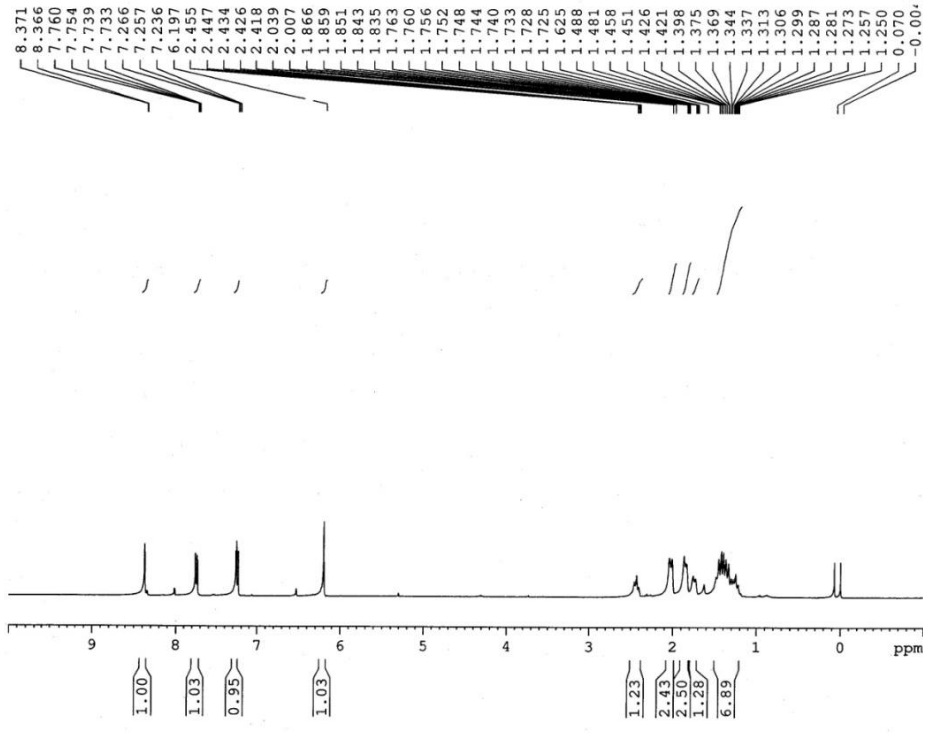

Figure S123: ^{1}H NMR spectrum of compound **35**.

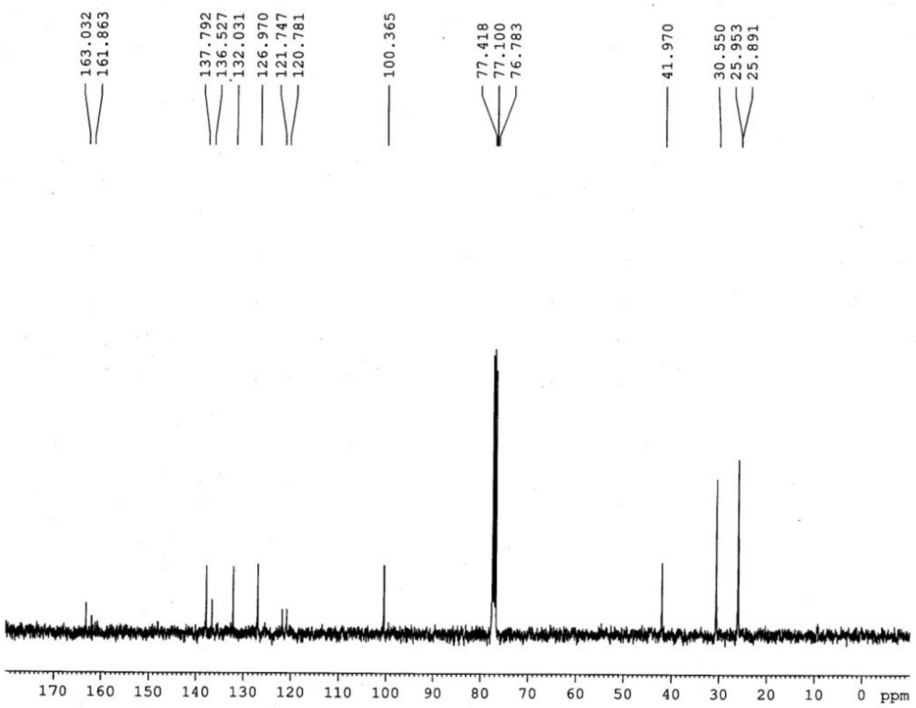

Figure S124: ^{13}C NMR spectrum of compound **35**.

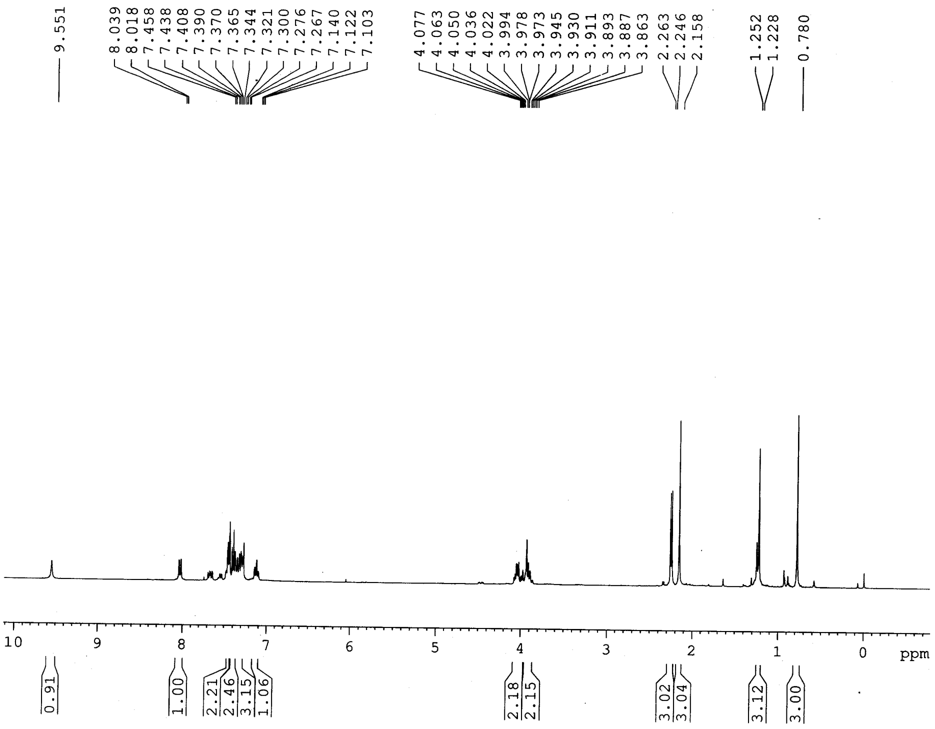

Figure S125: ^{1}H NMR spectrum of compound **A**.

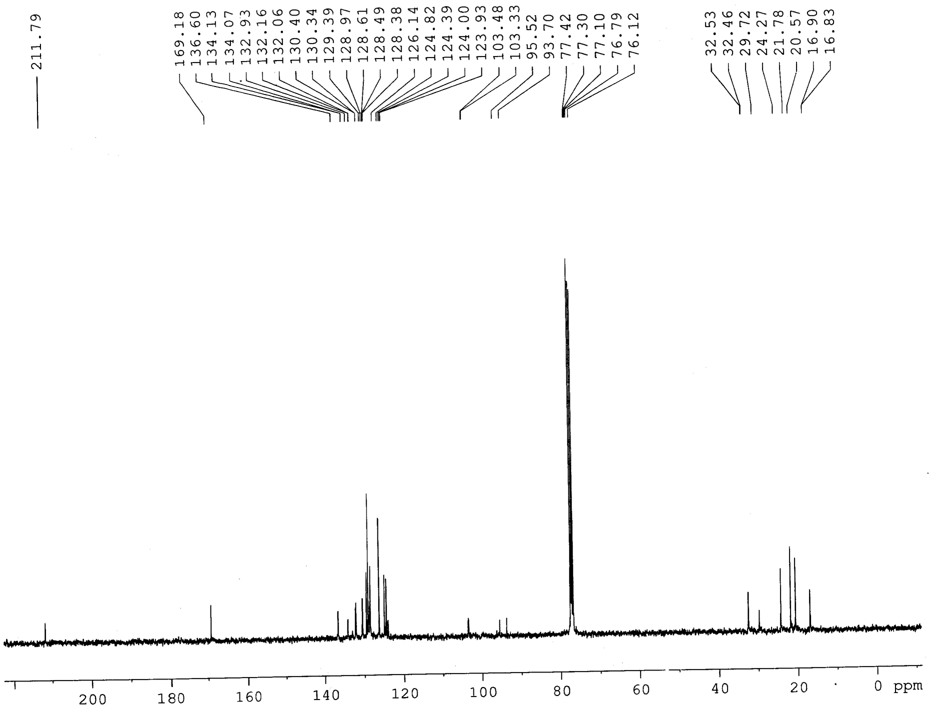

Figure S126: ^{13}C NMR spectrum of compound **A**.

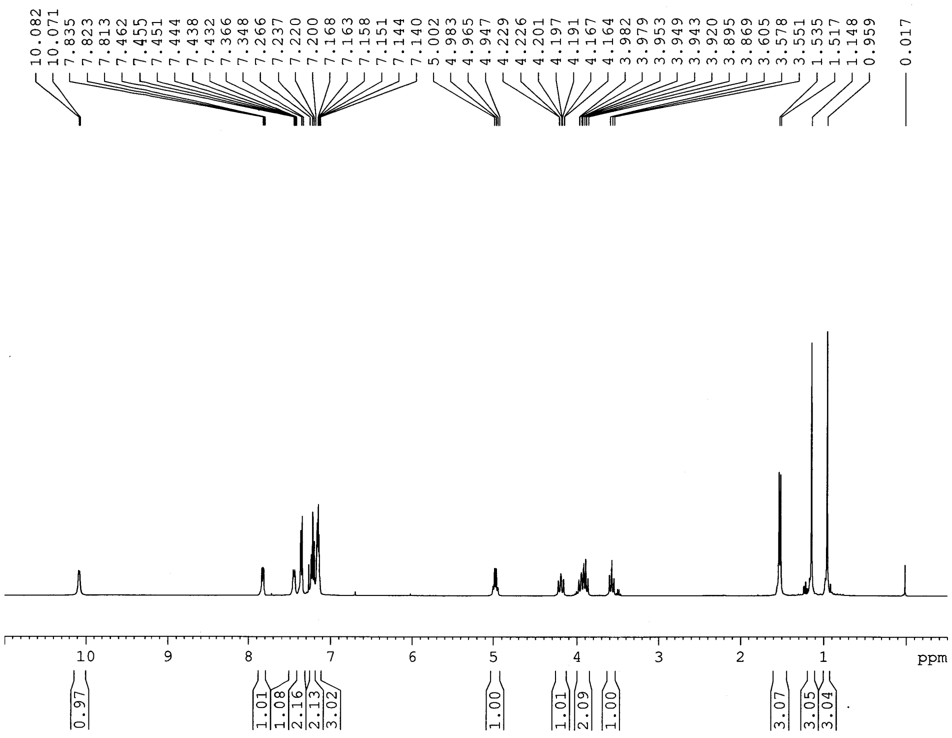

Figure S127: ^{1}H NMR spectrum of compound **B**.

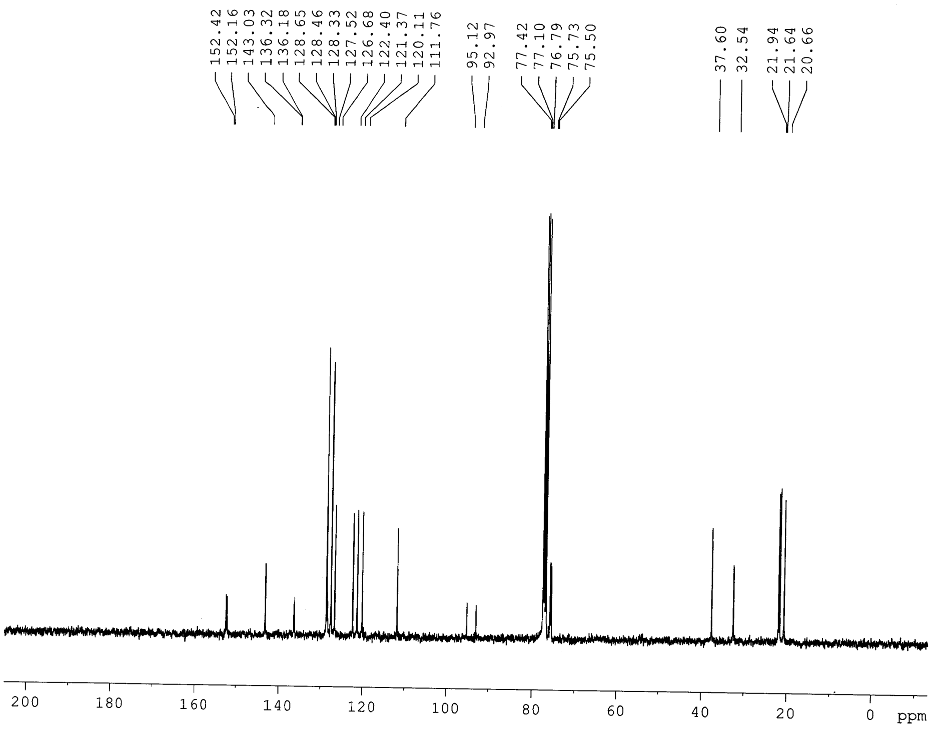

Figure S128: ^{13}C NMR spectrum of compound **B**.
